# Ecological drivers and habitat associations of estuarine bivalves

**DOI:** 10.7717/peerj.1348

**Published:** 2015-11-12

**Authors:** C. Seabird McKeon, Björn G. Tunberg, Cora A. Johnston, Daniel J. Barshis

**Affiliations:** 1 National Museum of Natural History—SMS, Smithsonian Institution, Fort Pierce, FL, USA; 2 Department of Biological and Environmental Sciences, Göteborg University, Kristineberg, Sweden; 3 Department of Entomology, University of Maryland, College Park, MD, USA; 4 Department of Biological Sciences, Old Dominion University, Norfolk, VA, USA

**Keywords:** Bivalve, Estuary, Biodiversity, Water flow, Salinity, Sediment

## Abstract

Community composition of the infaunal bivalve fauna of the St. Lucie Estuary and southern Indian River Lagoon, eastern Florida was sampled quarterly for 10 years as part of a long-term benthic monitoring program. A total of 38,514 bivalves of 137 taxa were collected and identified. We utilized this data, along with sediment samples and environmental measurements gathered concurrently, to assess the community composition, distribution, and ecological drivers of the infaunal bivalves of this estuary system. Salinity had the strongest influence on bivalve assemblage across the 15 sites, superseding the influences of sediment type, water turbidity, temperature and other environmental parameters. The greatest diversity was found in higher salinity euhaline sites, while the greatest abundance of individual bivalves was found in medium salinity mixohaline sites, the lowest diversity and abundances were found in the low salinity oligohaline sites, demonstrating a strong positive association between salinity and diversity/abundance. Water management decisions for the estuary should incorporate understanding of the role of salinity on bivalve diversity, abundance, and ecosystem function.

## Introduction

Estuaries are among the world’s most commercially important environments (Costanza et al., 1997). Bivalves not only constitute a major fisheries resource (Gosling, 2003), but also serve important roles in the ecology of soft-bottom estuarine communities by influencing species composition (Newell, 2004; Vaughn & Hakenkamp, 2001), sediment characteristics (Rhoads & Young, 1970), and the filtration of near-surface waters (Kiørboe & Møhlenberg, 1981). Less understood, however, are the patterns of spatial and temporal distribution within bivalve communities, and the functional diversity of these groups (Vaughn, Spooner & Galbraith, 2007). Estuarine and coastal soft-bottom communities are some of the ecosystems most likely to be impacted by human activity (Lotze et al., 2006), thus bivalves have also become important biomarkers of anthropogenic impact (Montagna & Kalke, 1995).

There are numerous potential ecological drivers of bivalve distributions; salinity (Wells, 1961), sediment composition (Rhoads & Young, 1970), temperature (McMahon, 1979), water flow (Dettman et al., 2004), larval transport (Wood & Hargis, 1971), and chemical pollutants (Doherty & Cherry, 1988) have previously been linked to bivalve occurrence, abundance, and growth. Recently, in the context of shell calcification, other potential factors that may influence bivalve distributions have been highlighted as indicators of changing marine conditions, such as pH and conductivity (Welladsen, Southgate & Heimann, 2010). Understanding how bivalve communities associate with an array of potential environmental gradients will help to identify indicator species and ecological groups linked to particular conditions—whether as a sign of health, recovery from disturbance, or the need for mitigation.

To examine the distribution of bivalves across a series of environmental gradients, our research focused on the St. Lucie Estuary and southern Indian River Lagoon (SLE-IRL). Located in southeastern Florida, the system is a part of one of the largest and most diverse estuarine systems in North America (Gilmore, 1995). As a part of land-use changes and wetland conversion to agricultural fields, freshwater outflow from Lake Okeechobee is diverted into the St. Lucie Estuary and nearby waterways in periodic releases. These releases have been implicated as major disturbances to the estuarine and saltwater fauna of the region (Gunter & Hall, 1963), though saline influences are thought to be an effect of the channelization of the St. Lucie Inlet (International-Americas, 1988). Land-use changes have also promoted changes in sediment types, particularly the proliferation of ‘muck’, a fine grained sediment rich in organic content (Trefry, 1996a; Trefry, 1996b) that covers the bottom of estuary channels in layers as deep or deeper than 1 m (Riegl, Foster & Foster, 2009). The molluscan fauna of the region was previously described in detailed work by Mikkelsen, Mikkelsen & Karlen (1995).

Studies of bivalve response to environmental change in the SLE-SIRL have been limited; prior studies have focused on economically important oysters and hard clams (Busby, 1986; Wilson et al., 2005). Given the diverse assemblage of bivalves found in the region, and continued concern and interest in understanding the role of anthropogenic change to the estuary, we examined the entire benthic infaunal assemblage of bivalve molluscs (hereafter ‘bivalves’) in the southern Indian River Lagoon and St. Lucie Estuary over a 9 year period to elucidate patterns of distribution and environmental drivers.

## Methods

The benthic monitoring project conducted by the Marine Ecology Laboratory at the Smithsonian Marine Station as a part of Everglades restoration activities (http://www.evergladesrestoration.gov) generated long-term data on the infaunal bivalves of the IRL-SLE, covering the years 2005–2014, sampled quarterly. We analyzed these data using non-parametric statistics to examine patterns of occurrence, community composition, distribution, and ecological drivers.

**Figure 1:**
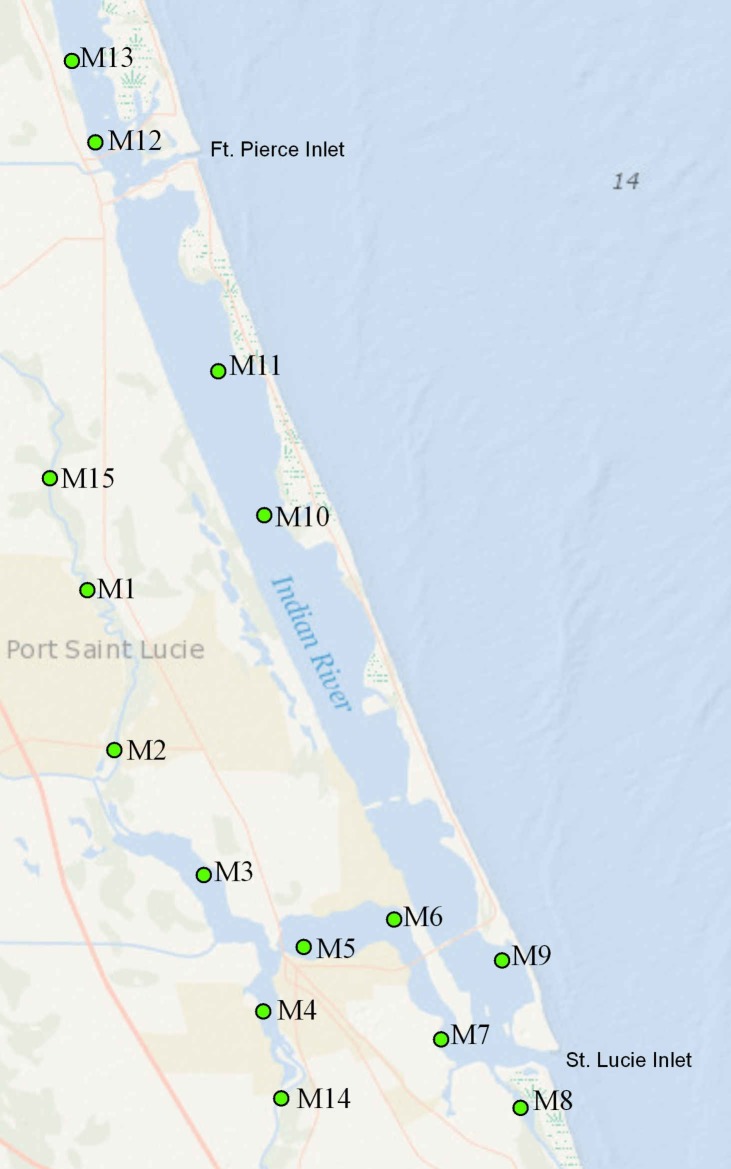
Map of the study area. Numbered points indicate sampling sites; “C” labels indicate canals.

The SLE-SIRL benthic sampling program is a fixed site monitoring effort directed at identifying trends in benthic condition and biodiversity. Sampling stations are situated outside of maintained channels, not on sandbars or spoil areas. Each station is submerged in at least 1 m of water at low tide, and less than 2 m of water at mean high tide. Sampling is conducted at 15 sites (Fig. 1). Each site is visited 4 times per year—in January and April (dry season) and July and October (wet season). These sites span all salinity regimes within the St. Lucie Estuary and the southern Indian River Lagoon (SLE-IRL) including Euhaline (30–40 parts per thousand), Mixohaline (5–30 ppt) and Oligohaline (0.5–5.0 ppt). The sites cover the watershed in such a way that benthic responses to hydrologic events stemming from the system’s tributaries can be detected and analyzed. Samples consist of 3 ponar grabs for fauna, a series of water metrics, and two cores for sediment characteristics from each site.

Infaunal invertebrates are collected utilizing a 0.02-m^2^ Petit Ponar grab (3 replicates per site). After being extracted through a sieve with 0.5-mm mesh size in the field, the samples are immediately preserved in a solution of 4–7% buffered formalin, diluted in sea water, and stained with Rose Bengal. Infaunal samples are stored in the buffered formalin solution for at least 1 week, then transferred to 70% ethanol and sorted by means of a stereo microscope (×6 magnification). All specimens are identified to the lowest possible taxon, and the number of individuals of each taxon is calculated.

Environmental variables including surface and bottom water temperature, conductivity, dissolved oxygen, pH, and salinity are measured at each site on each sampling occasion (YSI 556, and YSI ‘Professional Plus’ instruments). The turbidity is measured with both a Secchi disk (limnological), and a Hach 2100P Turbidimeter. The time of day and weather conditions are recorded at each sampling site on each occasion. This data is maintained at the Smithsonian Marine Station.

Bottom substrate samples for sediment analyses are collected by means of an Ogeechee corer. Two replicate cores are taken at each site, independently of Ponar grabs. The sediment from the cylinders is further divided into subsamples from two substrate depths (0–2 cm and 2–5 cm). The color of the sediment at ca 5-cm substrate depth is recorded. A general sediment type (sandy, silty, clay) on the basis of appearance and texture, and the absence/presence of hydrogen sulfide (H_2_S) odor are also determined. Sediment samples are frozen until processed in the lab. The frozen sediment samples are placed individually in pre-weighed aluminum containers. After weighing, these samples are placed individually in a drying oven (80 °C) until the sample has reached constant weight (usually about one week). When they have reached room temperature, the samples are weighed again and transferred to a muffle furnace (500 °C) for 5 h. After weighing (procedure described above), these samples are discarded. Water content, and percent loss on ignition (a coarse measure of organic content in the sediment) are calculated from these measurements. Separate sediment core sampling at a substrate depth of 0–5 cm are conducted at every site once per year for granulometric analyses following ICES standards, utilizing geological sieves of 4,000 µm, 2,000 µm, 500 µm, 250 µm, 125 µm, and 63 µm sizes in a Meinzer II Sieve shaker. Grain size distributions were calculated from geological sieves (Folk, 1980).

## Analysis

Annual sediment analysis was conducted using the program Gradistat (Blott & Pye, 2001). We utilized ‘geometric mean particle size’, along with ‘Percent Loss on Ignition’ at two different sediment depths, as descriptive environmental variables of sediment characteristics in total community analysis. Sediment types were defined using the modified Folk and Ward method supported by Blott & Pye (2001). More complete descriptions of the annual sediment samples are presented in Table 2.

Environmental variables were normalized, and evaluated using a Euclidian distance matrix in PRIMER- 6 (Clarke & Warwick, 2001), avoiding the application of Bray-Curtis measures to non-community data (Clarke & Gorley, 2006). Draftsman plots and Principle Components Analysis (PCA) were utilized to evaluate orthogonality and colinearity amongst environmental variables. Groups of variables found to be colinear were reduced to a single representative for graphical presentation.

Biological data was analyzed using the multivariate statistics package PRIMER-6 and the statistical programming environment R (Team, 2006). Only samples which contained at least one bivalve were included in the analysis (*n* = 1,029 grabs). Datapoints missing critical environmental measures (such as sediment) were excluded. The three grab subsamples were averaged within a single sampling date per site (*n* = 343). In some analyses and visualizations (such as nMDS), all samples for a single site were averaged. Diversity analyses included Species Richness, Shannon, and Simpson indices (Table 1). As a means of reducing zero-inflated skew, the bivalve data were square-root transformed and initially analyzed using a Bray-Curtis distance matrix for comparison within the dataset. Similarities among the bivalve communities at the 15 sites were visualized using nonmetric multidimensional scaling and measured using cluster analyses. Paired measures of similarity among the sites were tested with a Permutational MANOVA. Bivalve community composition and the dominance of individual species were compared between sites within the IRL-SLE using the SIMPER program (Clarke & Warwick, 2001).

**Table 1:** Diversity analysis of bivalves surveyed in the Indian River Lagoon and St. Lucie River.

Site	*S*	*N*	*d*	*J*′	*H*′ (loge)	1-Lambda′
M01	8	7.26	3.53	0.46	0.95	0.54
M02	24	165.21	4.50	0.35	1.11	0.59
M03	5	18.14	1.38	0.41	0.66	0.45
M04	8	35.99	1.95	0.05	0.10	0.03
M05	6	4.68	3.24	0.18	0.32	0.16
M06	30	10.46	12.35	0.52	1.77	0.74
M07	67	38.58	18.07	0.64	2.68	0.90
M08	55	29.73	15.92	0.68	2.74	0.93
M09	60	18.25	20.31	0.68	2.77	0.95
M10	32	19.39	10.46	0.73	2.52	0.94
M11	38	13.72	14.13	0.71	2.57	0.96
M12	59	47.14	15.05	0.60	2.45	0.89
M13	52	7.89	24.69	0.81	3.19	1.08
M14	15	91.96	3.10	0.32	0.87	0.39
M15	6	2.33	5.90	0.58	1.04	1.01

**Notes.**

*S* Total species *N* Average number of individuals *d* Species Richness (Margalef) *J*′ Pielou’s evenness *H*′ Shannon 1-Lambda′ Simpson

Links between the patterns of abundance of bivalve species in the faunal samples and environmental variables were sought utilizing the BEST routine in PRIMER. Permutation tests (999) allowed for the determination of statistical significance at the 1% level.

## Results

**Figure 2:**
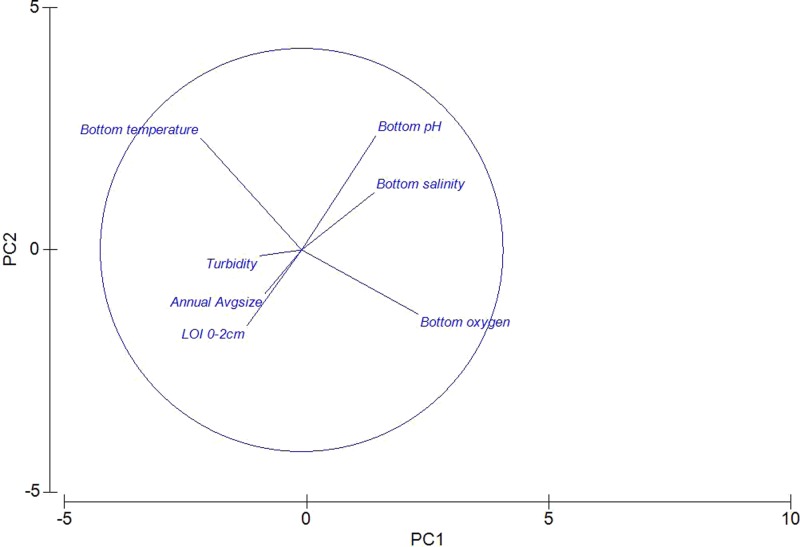
Principle components analysis of environmental measures taken across 15 sites in the Indian River Lagoon and St. Lucie River.

A Principle Components Analysis (PCA) was used to visualize the environmental data (Fig. 2). Colinearity of the same environmental variables at different depths (i.e., Surface O_2_, and Benthic O_2_) justified the removal of surface readings (data not shown). As expected, bottom temperature and bottom oxygen are nearly orthogonal, with oxygen declining with increased temperature. Sediment characteristics were grouped, with “Percent Loss on Ignition” and Sediment Geometric Average grain size roughly co-linear. Also visible is a negative relationship between declining pH, and increasing Loss on Ignition.

The bivalve dataset was composed of 38,514 individuals of 137 taxa. Bivalve richness ranged from 5 to 67 taxa per sample (at sites M7 and M3, respectively). Excluding samples where no bivalves occurred, average abundance ranged from 2.33 (sd = 2.7) to 165.21 (sd = 337.29) individuals per grab (at sites M15 and M2, respectively).

Permutational MANOVA (999 permutations, site as random factor) reveals that bivalve groups differed at each of the 15 sites (pseudo-F = 15.146, *P* = 0.001). Pair-wise comparisons within the Permutational MANOVA are suggestive that all groups differ from one another (*P* < 0.05), with the exception of sites M3 and M4, which did not significantly differ (*P* = 0.234).

**Figure 3:**
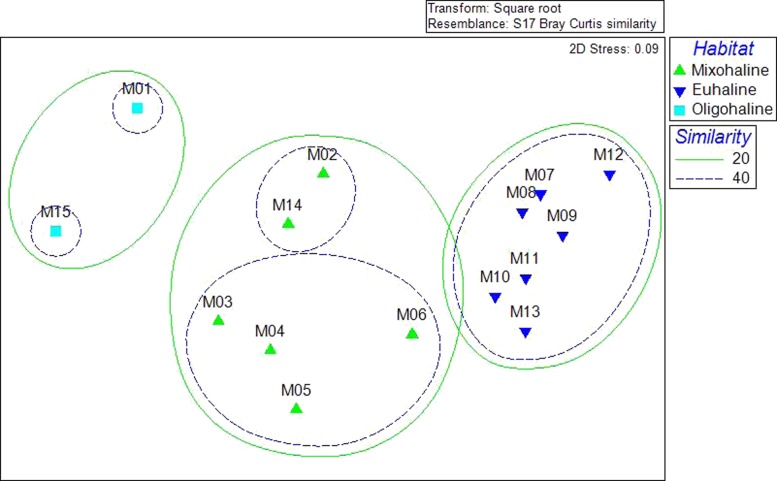
Cluster analysis of community resemblance, as nMDS overlay. The three clusters visible at the 20% level correspond to the average salinities: M1 and M15 are oligohaline (almost freshwater), M2–M6, M14 are mixohaline (brackish), M7–M13 are euhaline (saltwater).

A Bray-Curtis matrix of square root transformed abundance data was used in non-metric Multi Dimensional Scaling and related Cluster analysis to visualize patterns of similarity within the bivalve community. Clustering demonstrated statistically significant grouping at the 20, 40, and 60% similarity levels. Sites M1 and M15, grouped together at the 20% level. Sites M2, M14, M3, M4, M5, and M6, also grouped at 20%, with 40% similarity of two groupings: M2 and M14, and M3–M6. Sites (M7–M13) grouped at 40% similarity (Fig. 3). The three groupings of the community data at the 20% level correspond with salinity habitat descriptions. Sites M1 and M5 are oligohaline, with average salinities less than 5 ppt. Sites M2–M6 and M14 are mixohaline, while M7–M13 are euhaline.

Heterogeneity of Dispersion, here used as a measure of Beta Diversity following Anderson, Ellingsen & McArdle (2006), was calculated for the sites (alpha = 0.05). The highest values were found for site M13 (49.618), the lowest for site M3 (29.688). Beta Diversity was also calculated for the three habitat types established in Cluster Analysis (oligohaline, mixohaline, euhaline). Euhaline habitats in the SLE-IRL had the lowest heterogeneity (42.785, se = 0.8237), followed by mixohaline (48.075, se = 1.2357), and oligohaline (52.437, se = 2.8626).

The search for linkages between environmental parameters and the abundance data of the bivalve fauna was performed with the BEST procedure (Clarke & Warwick, 2001). Bottom salinity was the best descriptor of community composition, with a correlation between the Bray-Curtis faunal abundance matrix and the Euclidian matrix of environmental variables describing 51% of the pattern (BEST match permutation test, *p* < 0.001). Increasing the number of environmental variables contained in the model did not improve the correlation, with the strongest alternative descriptors being a two-variable combination of bottom salinity and bottom pH, capable of describing 34% of the combined matrices.

**Figure 4:**
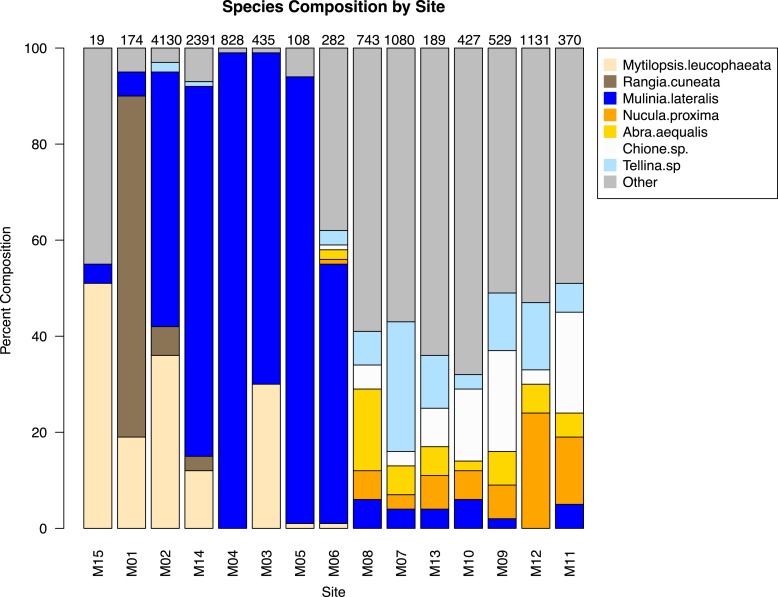
Species composition by site, arranged in order of salinity (increasing to the right). Dominant species are represented as individual colors, all other bivalve species together are coded as grey. Numbers at the top of each bar are the total number of individuals (subsamples averaged) at each site represented in the analysis.

**Figure 5:**
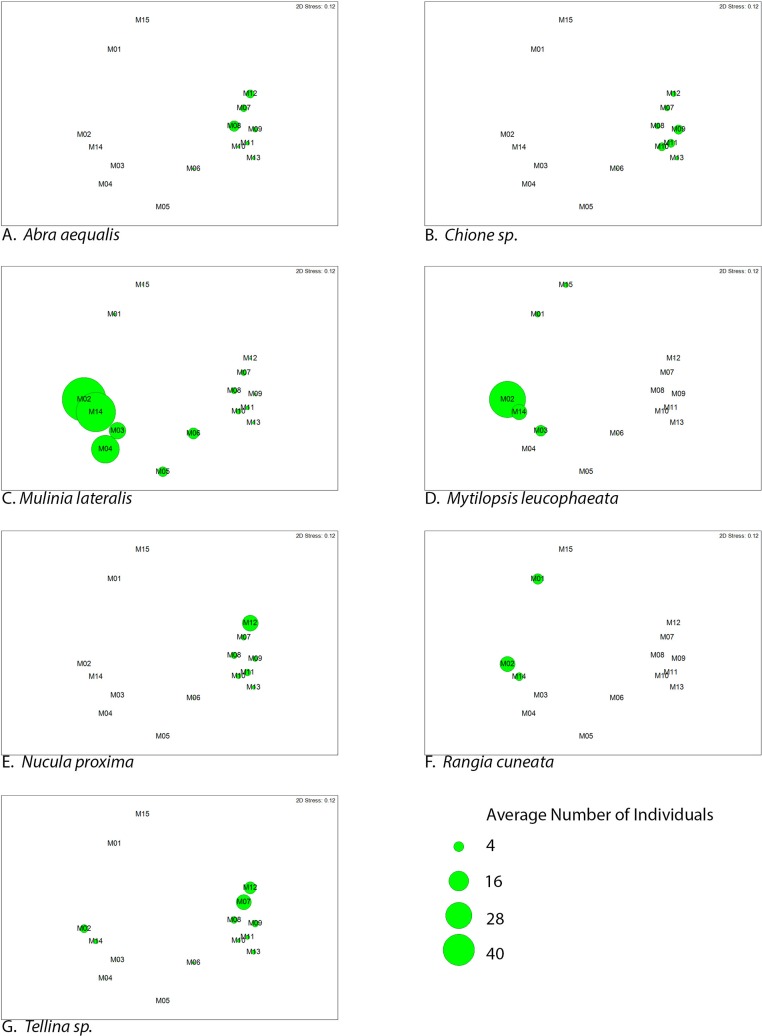
Average abundance (number of individuals) of dominant bivalve species represented as bubble-map overlay on nMDS plot of sample sites within the SLE-IRL. nMDS plot prepared with Bray-Curtis similarity matrix. Larger bubbles indicate higher abundance. Location of sites within the nMDS roughly corresponds with geographic locale, St. Lucie River Estuary to the left, Indian River Lagoon to the right.

The similarity percentages routine ‘SIMPER’ was used to examine the percent contribution of individual species to the community composition of individual sites (Dataset S1). Species that composed 20% or greater of the individuals found at any site were considered dominant taxa. Seven taxa fit this criterion: *Rangia cuneata*, *Mytilopsis leucophaeata*, *Mulinia lateralis*, *Tellina* sp., *Abra aequalis*, *Chione* sp., *Nucula proxima* (Fig. 4). Shifts in the abundance and, therefore, assemblage of these key species is evident along a gradient of salinity (Fig. 5). *Mulinia lateralis* for example, composes a large portion of the total bivalve abundance at mixohaline sites M2–M6 + M14, and quickly decreases in euhaline sites M7–M13 (Fig. 4).

## Discussion

In accordance with Wells (1961) and other classic studies, we found that patterns of bivalve community richness corresponded most strongly to bottom salinity. Other environmental measures related to sediment type and condition and to water pH, turbidity, and temperature—which play roles in bivalve community structure in other areas—did not play a major role in the structuring of the bivalve community in the IRL. Total abundance, however, was not tied to salinity, but instead was dominated by a few ‘opportunistic’ species capable of maximizing the resources available at sites that appear to be challenging for a more diverse community.

Two groupings are apparent within the bivalve community. Of our dominant taxa, *Mulinia lateralis, Mytilopsis leucophaeta*, and *Rangia cuneata* cluster within the St. Lucie River Estuary, while *Tellina* sp., *Abra aequalis*, *Chione* sp. and *Nucula proxima* are more frequently found within the Indian River Lagoon (Fig. 4). The division between these groups most clearly corresponds to a shift in salinity, but taxon-specific traits likely contribute to associations within each group.

All of the dominant species in our study have planktonic larvae, including *Rangia cuneata,* which has the greatest penetration upstream. Larval feeding behavior for these taxa is largely planktotrophic, or unknown, with only *Nucula proxima* having lecithotropic larvae (Mikkelsen, Mikkelsen & Karlen, 1995). Adult size may also be factor in the distribution of bivalves in the IRL-SLE, with larger, heavy shelled taxa such as *Chione* found almost exclusively in the lower reaches of the system, while smaller lighter shelled species such as *R. cuneata* and *Mulinia lateralis* were competent and abundant in the more loosely aggregated sediment types of the middle reaches of the estuary.

Suspension feeders were found throughout our study area, but the three deposit feeders (*Abra aequalis, Tellina* sp., *Nucula proxima*; Mikkelsen, Mikkelsen & Karlen, 1995) were largely restricted to the most saline sites, suggesting that the lower salinity mixohaline sites are entirely lacking this guild of bivalves. This is particularly interesting in that benthic suspension feeders and deposit feeders generally do not occur at the same sites (Dame, 2012), but in this dataset, deposit feeders are limited to sites shared with suspension feeders. This may also explain the higher diversity at high salinity sites. The composition of suspended sediments may impact the feeding ecology of benthic organisms through variation in the ability to separate organic and inorganic particles (Galimany, Ramon & Ibarrola, 2011), or ecological impacts of turbidity (Lunt & Smee, 2014), when at levels above those observed in the study area.

Sites with the greatest freshwater influence (predominantly within SLE) had exceptionally low diversity/richness. The Oligohaline site M1 had a single dominant taxon- *Rangia cuneata.* Of the ‘native’ bivalve fauna, *R. cuneata* has the greatest tolerance for low salinity environments (Henry & Mangum, 1980). *R. cuneata* was believed extirpated from the Atlantic coast, before a recent expansion (Hopkins & Andrews, 1970). Invasive freshwater clams *Corbicula flaminea*, were also detected at site M14, our site closest to the freshwater outflowing from Lake Okeechobee via the C44 canal (Fig. 1). Total numbers were low (*n* = 264), but composed up to 43% of the limited bivalve samples from this area at some times.

*Mulinia lateralis* had the most variable occurrence—spanning many conditions—of any species studied here. Previous work on *M. lateralis* has supported the idea that while the adults are capable of withstanding great variability in environment, the larvae are less robust to salinity change (Lough, 1974). *M. lateralis* was also among the ‘r selected’ species found to dominate post-catastrophic benthic invertebrate populations in Tampa Bay (Santos & Simon, 1980). The apparent dietary preference of this species for suspended bacterial particles (Chalermwat, Jacobson & Lutz, 1991) corresponds well with the “poor water quality” (i.e., aqueous muck, low bottom oxygen content, etc.) observed at sites where it is most abundant. This suggests that the tremendous variability seen in this species’ occurrence could also be a result of salinity ‘windows of opportunity’ for the larvae to colonize polyhaline sites out of the range of more stenohaline competitors, and a tolerance for “muck” at poor water quality sites.

*Mulinia lateralis* was the single most abundant bivalve taxon, with an average of 15.79 individuals per sample (range = 0–1,885), and a total of 16,251 individuals included in this study. *M. lateralis* reach peak abundance at site M2, with a much reduced presence in areas of greater bivalve diversity (sites M7–M13) (Fig. 4), which likely indicates the dynamics of an opportunist that reaches greater densities when released from competition from other bivalve species (Chalermwat, Jacobson & Lutz, 1991). Thus, future research could reveal interesting effects of direct or indirect interactions within the bivalve communities.

Among the species associated with the saltier IRL, *Tellina* sp. apparently avoid the sites with the most total environmental variability (M3–M5), but do appear at M2 and M14, which are mixohaline (Fig. 5). The sites with the most abundant *Tellina* sp. shared variable salinity, but fine-grained sand, suggesting that sediment drives the distribution of this particular taxon (Table 2).

**Table 2:** Granulometric analysis of sediment distribution and classification arranged by sampling site.

	%4 mm	%2 mm	%500 μm	%250 μm	%125 μm	%63 μm	%<63 μm		
M1	0.15	0.15	0.73	0.97	55.45	41.53	1.02		
		0.30		1.70		96.98	1.02	100.00	FINE SAND
M2	14.62	2.43	5.00	3.69	30.94	38.25	5.06		
		17.05		8.69		69.20	5.06	100.00	COARSE SANDY FINE SAND (W. GRAVEL)
M3	1.66	9.61	8.13	9.94	21.15	14.42	35.09		
		11.27		18.06		35.57	35.09	100.00	SAND FINE/COARSE SAND/CLAY (W. GRAVEL)
M4	5.19	5.10	3.13	4.04	12.26	15.65	54.62		
		10.29		7.17		27.91	54.62	100.00	FINE SANDY CLAY (W. GRAVEL)
M5	1.50	4.38	5.95	5.69	14.87	13.43	54.18		
		5.88		11.64		28.30	54.18	100.00	FINE SANDY CLAY
M6	0.58	1.80	13.00	29.31	15.14	14.24	25.93		
		2.38		42.31		29.38	25.93	100.00	SAND FINE/COARSE SAND/CLAY
M7	0.01	0.07	0.45	5.69	21.82	63.65	8.32		
		0.08		6.14		85.47	8.32	100.00	FINE SAND
M8	0.04	0.23	1.74	17.54	30.31	41.12	9.02		
		0.27		19.28		71.43	9.02	100.00	COARSE SANDY FINE SAND
M9	0.38	0.32	5.37	44.63	31.28	13.26	4.76		
		0.70		50.00		44.54	4.76	100.00	CLAYEY COARSE SAND
M10	1.27	0.12	0.36	1.10	21.64	68.95	6.56		
		1.39		1.46		90.58	6.56	100.00	FINE SAND
M11	0.20	0.07	0.33	1.05	17.23	75.41	5.70		
		0.27		1.38		92.64	5.70	100.00	FINE SAND
M12	0.07	0.31	4.72	43.88	27.16	18.62	5.25		
		0.37		48.60		45.78	5.25	100.00	CLAYEY COARSE SAND
M13	1.86	1.02	4.66	38.93	38.00	9.58	5.95		
		2.88		43.59		47.57	5.95	100.00	COARSE SANDY FINE SAND
M14	0.63	0.93	1.30	1.74	34.38	54.43	6.59		
		1.56		3.04		88.81	6.59	100.00	FINE SAND
M15	0.20	0.61	2.36	8.28	34.56	30.78	23.22		
		0.80		10.63		65.34	23.22	100.00	CLAYEY FINE SAND

Other ecological factors not included in this study may impact the abundance and distribution of estuarine bivalves and contribute to the patterns observed. Seasonal estuarine waterfowl such as Lesser Scaup (*Aythya affinis*) are active predators on bivalve molluscs (Badzinski & Petrie, 2006), as are locally important fishes such as Black Drum (*Pogonias cromis*) (Brown et al., 2008). Crabs of several families are important modifiers of bivalve communities in southeastern estuaries (Johnson, Grabowski & Smee, 2014). Interactions between predation and physical parameters such as turbidity (Lunt & Smee, 2014) may also have large scale impacts on community structures that are not attributable in the data presented here.

## Conclusion

Throughout all analysis, salinity regime was the dominant influencing factor. Salinity changes within the IRL-SLE have a long history with human use of the area (Trefry, 1996a), coming from the hardening of inlets, increases in water-impermeable surfaces in the watershed, and direct releases of water-storage from Lake Okeechobee (Kim et al., 2002; Sime, 2005). The fluctuation of salinity within the study area is likely to have large effects on the salinity-driven benthic community documented here. Temporal patterns in salinity effects will be described in a forthcoming manuscript. The role of altered freshwater flow regimes and salinity in altering the ecological characteristics of individual bivalve species is well documented from estuaries around the world, including the Colorado River Delta (Schone et al., 2003), and has been one of several factors considered in the reduction of seagrass in the Indian River Lagoon (Fletcher & Fletcher, 1995).

The results of this study, when viewed with the importance of bivalves as predictors of environmental change, determinants of water quality, and ecosystem engineers, inform decision making for the region. One result is a demonstration of the primacy of salinity in the development of bivalve community structure in the area. The manipulation of salinity in this ecosystem will have predictable, detectable consequences on the bivalve community. The abundance and apparent proliferation of muck sediments in the southern IRL and St. Lucie River Estuary has led to management efforts to curb their spread (Zhang et al., 2003). Our long-term invertebrate sampling reveals that while muck sediments may be a limitation for some species, the overall importance of these sediments to bivalve community structure in the IRL-SLE is minor in comparison to salinity. Management strategies for sediment composition and other issues affecting the IRL-SLE need to be paired with salinity control, as one without the other will likely be ineffective in shifting community diversity and overall lagoon health.

## Supplemental Information

10.7717/peerj.1348/supp-1 Supplemental Information 1 Simplified dataset

10.7717/peerj.1348/supp-2 Supplemental Information 2 Table of bivalve taxa and average abundances in a sample

10.7717/peerj.1348/supp-3 Dataset S1 Similarity Percentages—species contributions
