# Supplementary material for: Ecological drivers and habitat associations of estuarine bivalves
Source: PeerJ. 2015 Nov 12;3:e1348. doi: 10.7717/peerj.1348 (PMC4647571; doi:10.7717/peerj.1348)
Supplement: Dataset S1 [file peerj-03-1348-s003.rtf]

SIMPER
Similarity Percentages - species contributions

One-Way Analysis

Data worksheet
Name: Data7
Data type: Abundance
Sample selection: All
Variable selection: All

Parameters
Resemblance: S17 Bray Curtis similarity
Cut off for low contributions: 90.00%

Factor Groups
Sample	Site
M02Apr2007	M02
M02Jul2007	M02
M02Oct2007	M02
M02Jan2008	M02
M02Apr2008	M02
M02Jul2008	M02
M02Oct2008	M02
M02Jan2009	M02
M02Apr2009	M02
M02Jul2009	M02
M02Sept2009	M02
M02Jan2010	M02
M02Apr2010	M02
M02Jul2010	M02
M02Oct2010	M02
M02Jan2011	M02
M02Apr2011	M02
M02Jul2011	M02
M02Oct2011	M02
M02Jan2012	M02
M02Apr2012	M02
M02Jan2013	M02
M02Apr2013	M02
M02Jul2013	M02
M02Oct2013	M02
M03Apr2007	M03
M03Jul2007	M03
M03Jan2008	M03
M03Apr2008	M03
M03Jul2008	M03
M03Oct2008	M03
M03Jan2009	M03
M03Apr2009	M03
M03Jul2009	M03
M03Sept2009	M03
M03Jan2010	M03
M03Apr2010	M03
M03Jul2010	M03
M03Jan2011	M03
M03Apr2011	M03
M03Jul2011	M03
M03Oct2011	M03
M03Apr2012	M03
M03Jan2013	M03
M03Apr2013	M03
M03Jul2013	M03
M03Jan2014	M03
M03Apr2014	M03
M03Jul2014	M03
M04Apr2007	M04
M04Jul2007	M04
M04Apr2008	M04
M04Jul2008	M04
M04Oct2008	M04
M04Jan2009	M04
M04Apr2009	M04
M04Jul2009	M04
M04Apr2010	M04
M04Oct2010	M04
M04Jan2011	M04
M04Apr2011	M04
M04Jul2011	M04
M04Oct2011	M04
M04Jan2012	M04
M04Apr2012	M04
M04Jan2013	M04
M04Apr2013	M04
M04Jul2013	M04
M04Oct2013	M04
M04Jan2014	M04
M04Apr2014	M04
M04Jul2014	M04
M05Apr2007	M05
M05Jul2007	M05
M05Oct2007	M05
M05Jan2008	M05
M05Apr2008	M05
M05Jul2008	M05
M05Jan2009	M05
M05Apr2009	M05
M05Jul2009	M05
M05Sept2009	M05
M05Jan2010	M05
M05Apr2010	M05
M05Jul2010	M05
M05Jan2011	M05
M05Apr2011	M05
M05Jul2011	M05
M05Oct2011	M05
M05Jan2012	M05
M05Apr2012	M05
M05Oct2013	M05
M05Jan2014	M05
M05Apr2014	M05
M05Jul2014	M05
M06Apr2007	M06
M06Jul2007	M06
M06Oct2007	M06
M06Jan2008	M06
M06Apr2008	M06
M06Jul2008	M06
M06Oct2008	M06
M06Jan2009	M06
M06Apr2009	M06
M06Jul2009	M06
M06Sept2009	M06
M06Jan2010	M06
M06Apr2010	M06
M06Jul2010	M06
M06Oct2010	M06
M06Jan2011	M06
M06Apr2011	M06
M06Jul2011	M06
M06Oct2011	M06
M06Jan2012	M06
M06Apr2012	M06
M06Jan2013	M06
M06Apr2013	M06
M06Jul2013	M06
M06Oct2013	M06
M06Jan2014	M06
M06Apr2014	M06
M07Apr2007	M07
M07Jul2007	M07
M07Jan2008	M07
M07Apr2008	M07
M07Jul2008	M07
M07Oct2008	M07
M07Jan2009	M07
M07Apr2009	M07
M07Jul2009	M07
M07Sept2009	M07
M07Jan2010	M07
M07Apr2010	M07
M07Jul2010	M07
M07Oct2010	M07
M07Jan2011	M07
M07Apr2011	M07
M07Jul2011	M07
M07Oct2011	M07
M07Jan2012	M07
M07Apr2012	M07
M07Oct2012	M07
M07Jan2013	M07
M07Apr2013	M07
M07Jul2013	M07
M07Oct2013	M07
M07Jan2014	M07
M07Apr2014	M07
M07Jul2014	M07
M08Apr2007	M08
M08Jul2007	M08
M08Oct2007	M08
M08Jan2008	M08
M08Apr2008	M08
M08Jul2008	M08
M08Oct2008	M08
M08Jan2009	M08
M08Apr2009	M08
M08Jul2009	M08
M08Sept2009	M08
M08Jan2010	M08
M08Apr2010	M08
M08Jul2010	M08
M08Oct2010	M08
M08Jan2011	M08
M08Apr2011	M08
M08Jul2011	M08
M08Apr2012	M08
M08Oct2012	M08
M08Jan2013	M08
M08Apr2013	M08
M08Jul2013	M08
M08Oct2013	M08
M08Jan2014	M08
M09Apr2007	M09
M09Jul2007	M09
M09Oct2007	M09
M09Jan2008	M09
M09Apr2008	M09
M09Jul2008	M09
M09Oct2008	M09
M09Jan2009	M09
M09Apr2009	M09
M09Jul2009	M09
M09Sept2009	M09
M09Jan2010	M09
M09Apr2010	M09
M09Jul2010	M09
M09Oct2010	M09
M09Jan2011	M09
M09Apr2011	M09
M09Jul2011	M09
M09Oct2011	M09
M09Jan2012	M09
M09Apr2012	M09
M09Oct2012	M09
M09Jan2013	M09
M09Apr2013	M09
M09Jul2013	M09
M09Oct2013	M09
M09Jan2014	M09
M09Apr2014	M09
M09Jul2014	M09
M10Apr2007	M10
M10Jul2007	M10
M10Oct2007	M10
M10Jan2008	M10
M10Apr2008	M10
M10Jul2008	M10
M10Oct2008	M10
M10Jan2009	M10
M10Apr2009	M10
M10Apr2010	M10
M10Jul2010	M10
M10Oct2010	M10
M10Jan2011	M10
M10Apr2011	M10
M10Jul2011	M10
M10Oct2011	M10
M10Jan2012	M10
M10Apr2012	M10
M10Jan2013	M10
M10Apr2013	M10
M10Jul2013	M10
M10Oct2013	M10
M11Apr2007	M11
M11Jul2007	M11
M11Oct2007	M11
M11Jan2008	M11
M11Apr2008	M11
M11Jul2008	M11
M11Oct2008	M11
M11Jan2009	M11
M11Apr2009	M11
M11Jul2009	M11
M11Oct2009	M11
M11Apr2010	M11
M11Jul2010	M11
M11Oct2010	M11
M11Jan2011	M11
M11Apr2011	M11
M11Jul2011	M11
M11Oct2011	M11
M11Jan2012	M11
M11Apr2012	M11
M11Jan2013	M11
M11Apr2013	M11
M11Jul2013	M11
M11Oct2013	M11
M11Jan2014	M11
M11Apr2014	M11
M11Jul2014	M11
M12Apr2007	M12
M12Jul2007	M12
M12Oct2007	M12
M12Apr2008	M12
M12Jul2008	M12
M12Oct2008	M12
M12Jan2009	M12
M12Apr2009	M12
M12Jul2009	M12
M12Jan2010	M12
M12Apr2010	M12
M12Jul2010	M12
M12Oct2010	M12
M12Jan2011	M12
M12Apr2011	M12
M12Jul2011	M12
M12Oct2011	M12
M12Jan2012	M12
M12Apr2012	M12
M12Jan2013	M12
M12Apr2013	M12
M12Jul2013	M12
M12Oct2013	M12
M12Jan2014	M12
M13Apr2007	M13
M13Jul2007	M13
M13Oct2007	M13
M13Jan2008	M13
M13Apr2008	M13
M13Jul2008	M13
M13Oct2008	M13
M13Jan2009	M13
M13Apr2009	M13
M13Jul2009	M13
M13Jan2010	M13
M13Apr2010	M13
M13Jul2010	M13
M13Oct2010	M13
M13Jan2011	M13
M13Apr2011	M13
M13Jul2011	M13
M13Oct2011	M13
M13Jan2012	M13
M13Apr2012	M13
M13Jan2013	M13
M13Apr2013	M13
M13Jul2013	M13
M13Oct2013	M13
M01Jul2007	M01
M01Oct2007	M01
M01Apr2008	M01
M01Jul2008	M01
M01Oct2008	M01
M01Apr2009	M01
M01Jul2009	M01
M01Jan2010	M01
M01Apr2010	M01
M01Jul2010	M01
M01Oct2010	M01
M01Jan2011	M01
M01Apr2011	M01
M01Jul2011	M01
M01Oct2011	M01
M01Jan2012	M01
M01Apr2012	M01
M01Jan2013	M01
M01Apr2013	M01
M01Jul2013	M01
M01Oct2013	M01
M01Jan2014	M01
M01Apr2014	M01
M01Jul2014	M01
M14Oct2007	M14
M14Jan2008	M14
M14Apr2008	M14
M14Jul2008	M14
M14Oct2008	M14
M14Jan2009	M14
M14Apr2009	M14
M14Jul2009	M14
M14Sept2009	M14
M14Jan2010	M14
M14Apr2010	M14
M14Jul2010	M14
M14Oct2010	M14
M14Jan2011	M14
M14Apr2011	M14
M14Jul2011	M14
M14Oct2011	M14
M14Jan2012	M14
M14Apr2012	M14
M14Jan2013	M14
M14Apr2013	M14
M14Jul2013	M14
M14Oct2013	M14
M14Jan2014	M14
M14Apr2014	M14
M14Jul2014	M14
M15Jul2008	M15
M15Jan2009	M15
M15Jul2009	M15
M15Apr2011	M15
M15Jan2012	M15
M15Jan2013	M15
M15Apr2013	M15
M15Jul2013	M15

Group M02
Average similarity: 26.45

Species	Av.Abund	Av.Sim	Sim/SD	Contrib%	Cum.%
Mytilopsis leucophaeata	    4.21	  9.41	  0.91	   35.57	35.57
Rangia cuneata	    2.40	  9.19	  0.77	   34.73	70.30
Mulinia lateralis	    4.29	  3.73	  0.38	   14.08	84.38
Tellina sp	    1.17	  2.68	  0.54	   10.13	94.51

Group M03
Average similarity: 45.67

Species	Av.Abund	Av.Sim	Sim/SD	Contrib%	Cum.%
Mulinia lateralis	    2.92	 42.88	  1.35	   93.91	93.91

Group M04
Average similarity: 38.57

Species	Av.Abund	Av.Sim	Sim/SD	Contrib%	Cum.%
Mulinia lateralis	    4.11	 37.20	  1.35	   96.45	96.45

Group M05
Average similarity: 38.46

Species	Av.Abund	Av.Sim	Sim/SD	Contrib%	Cum.%
Mulinia lateralis	    1.50	 36.05	  1.20	   93.73	93.73

Group M06
Average similarity: 28.99

Species	Av.Abund	Av.Sim	Sim/SD	Contrib%	Cum.%
Mulinia lateralis	    1.51	 13.67	  1.15	   47.14	47.14
Tagelus divisus	    0.83	  5.39	  0.72	   18.58	65.72
Abra aequalis	    0.34	  3.38	  0.46	   11.67	77.40
Tellina sp	    0.34	  2.21	  0.41	    7.61	85.01
Chione sp.	    0.18	  1.20	  0.29	    4.15	89.15
Macoma tenta	    0.29	  1.17	  0.27	    4.05	93.20

Group M07
Average similarity: 35.91

Species	Av.Abund	Av.Sim	Sim/SD	Contrib%	Cum.%
Tellina sp	    2.55	  7.69	  1.03	   21.41	21.41
Parvilucina multilineata	    1.77	  5.61	  0.95	   15.61	37.03
Abra aequalis	    1.30	  5.09	  2.30	   14.18	51.21
Macoma tenta	    1.55	  2.90	  0.63	    8.09	59.29
Nucula proxima	    0.87	  2.53	  0.86	    7.04	66.33
Chione sp.	    0.85	  2.18	  0.73	    6.07	72.39
Tagelus divisus	    0.82	  2.01	  0.75	    5.60	77.99
Lyonsia floridana	    0.65	  1.82	  0.78	    5.06	83.06
Mulinia lateralis	    0.68	  1.56	  0.63	    4.34	87.40
Anadara sp.	    0.40	  0.71	  0.44	    1.98	89.38
Lucina sp.	    0.45	  0.69	  0.33	    1.91	91.29

Group M08
Average similarity: 43.18

Species	Av.Abund	Av.Sim	Sim/SD	Contrib%	Cum.%
Abra aequalis	    2.05	  9.51	  2.29	   22.03	22.03
Tagelus divisus	    1.96	  6.58	  1.22	   15.23	37.26
Mulinia lateralis	    1.09	  4.11	  1.17	    9.52	46.78
Parvilucina multilineata	    1.08	  4.03	  1.13	    9.33	56.11
Chione sp.	    0.96	  3.52	  1.09	    8.15	64.25
Nucula proxima	    1.08	  3.50	  1.23	    8.10	72.35
Tellina sp	    1.03	  2.66	  0.69	    6.15	78.50
Macoma tenta	    0.97	  2.25	  0.68	    5.21	83.71
Corbula sp.	    0.69	  1.79	  0.67	    4.15	87.86
Caryocorbula sp.	    0.79	  1.55	  0.50	    3.59	91.44

Group M09
Average similarity: 39.76

Species	Av.Abund	Av.Sim	Sim/SD	Contrib%	Cum.%
Chione sp.	    1.71	  9.67	  1.47	   24.33	24.33
Nucula proxima	    0.97	  5.39	  1.36	   13.55	37.88
Abra aequalis	    0.87	  4.11	  1.07	   10.33	48.21
Tellina sp	    1.06	  4.09	  0.78	   10.30	58.50
Tagelus divisus	    1.05	  3.79	  0.78	    9.53	68.04
Macoma tenta	    0.70	  2.03	  0.58	    5.10	73.13
Corbula sp.	    0.51	  1.98	  0.67	    4.99	78.13
Parvilucina multilineata	    0.62	  1.82	  0.53	    4.57	82.70
Lyonsia floridana	    0.44	  1.54	  0.63	    3.88	86.58
Lucina sp.	    0.59	  1.47	  0.41	    3.69	90.27

Group M10
Average similarity: 35.20

Species	Av.Abund	Av.Sim	Sim/SD	Contrib%	Cum.%
Chione sp.	    1.52	 10.61	  1.31	   30.14	30.14
Macoma tenta	    1.04	  5.23	  0.78	   14.85	44.99
Mysella sp.	    1.26	  4.79	  0.55	   13.60	58.59
Mulinia lateralis	    0.79	  3.38	  0.64	    9.60	68.18
Nucula proxima	    0.71	  2.49	  0.66	    7.08	75.26
Caryocorbula sp.	    0.86	  2.48	  0.52	    7.03	82.29
Tagelus divisus	    0.82	  2.27	  0.57	    6.44	88.73
Abra aequalis	    0.35	  1.13	  0.48	    3.20	91.93

Group M11
Average similarity: 28.91

Species	Av.Abund	Av.Sim	Sim/SD	Contrib%	Cum.%
Chione sp.	    1.24	  6.74	  0.84	   23.31	23.31
Nucula proxima	    0.95	  4.82	  0.76	   16.65	39.97
Macoma tenta	    0.69	  3.44	  0.73	   11.88	51.85
Mulinia lateralis	    0.54	  2.59	  0.57	    8.96	60.81
Tagelus divisus	    0.86	  2.20	  0.44	    7.62	68.43
Tellina sp	    0.53	  2.02	  0.41	    6.99	75.42
Corbula sp.	    0.47	  1.96	  0.50	    6.79	82.22
Caryocorbula sp.	    0.39	  1.94	  0.39	    6.71	88.92
Abra aequalis	    0.49	  1.68	  0.45	    5.81	94.74

Group M12
Average similarity: 38.14

Species	Av.Abund	Av.Sim	Sim/SD	Contrib%	Cum.%
Nucula proxima	    2.94	 11.44	  1.62	   29.99	29.99
Parvilucina multilineata	    1.93	  4.39	  0.74	   11.51	41.51
Tellina sp	    1.71	  4.37	  0.58	   11.45	52.95
Abra aequalis	    1.38	  4.28	  1.24	   11.23	64.18
Chione sp.	    0.95	  3.79	  1.56	    9.93	74.11
Macoma tenta	    1.68	  3.64	  0.74	    9.55	83.66
Crassinella lunulata	    0.47	  1.19	  0.65	    3.11	86.78
Tagelus divisus	    0.89	  1.12	  0.48	    2.93	89.71
Lyonsia floridana	    0.48	  1.09	  0.52	    2.85	92.56

Group M13
Average similarity: 22.97

Species	Av.Abund	Av.Sim	Sim/SD	Contrib%	Cum.%
Chione sp.	    0.59	  4.47	  0.75	   19.46	19.46
Nucula proxima	    0.56	  4.07	  0.72	   17.72	37.18
Macoma tenta	    0.62	  2.72	  0.58	   11.86	49.03
Tellina sp	    0.57	  2.31	  0.51	   10.04	59.07
Abra aequalis	    0.42	  1.87	  0.53	    8.15	67.22
Anadara sp.	    0.32	  1.00	  0.37	    4.36	71.58
Parvilucina multilineata	    0.25	  0.99	  0.33	    4.33	75.90
Mulinia lateralis	    0.30	  0.86	  0.31	    3.75	79.66
Lyonsia floridana	    0.21	  0.66	  0.28	    2.88	82.53
Lucina sp.	    0.24	  0.65	  0.23	    2.85	85.38
Sphenia sp.	    0.25	  0.62	  0.28	    2.69	88.07
Caryocorbula sp.	    0.18	  0.62	  0.27	    2.69	90.76

Group M01
Average similarity: 31.33

Species	Av.Abund	Av.Sim	Sim/SD	Contrib%	Cum.%
Rangia cuneata	    1.59	 25.13	  0.93	   80.21	80.21
Mytilopsis leucophaeata	    0.71	  4.84	  0.45	   15.45	95.66

Group M14
Average similarity: 31.87

Species	Av.Abund	Av.Sim	Sim/SD	Contrib%	Cum.%
Mytilopsis leucophaeata	    2.46	 16.00	  0.87	   50.21	50.21
Mulinia lateralis	    4.89	  9.62	  0.60	   30.20	80.41
Rangia cuneata	    0.91	  3.34	  0.50	   10.48	90.89

Group M15
Average similarity: 30.40

Species	Av.Abund	Av.Sim	Sim/SD	Contrib%	Cum.%
Bivalvia	    0.77	 28.00	  0.93	   92.10	92.10

Groups M02  &  M03
Average dissimilarity = 85.32

	Group M02	Group M03	       	       	        	     
Species	 Av.Abund	 Av.Abund	Av.Diss	Diss/SD	Contrib%	Cum.%
Mulinia lateralis	     4.29	     2.92	  26.50	   1.35	   31.05	31.05
Mytilopsis leucophaeata	     4.21	     0.95	  20.15	   0.98	   23.61	54.67
Rangia cuneata	     2.40	     0.00	  15.87	   1.02	   18.60	73.27
Tellina sp	     1.17	     0.10	   6.29	   0.92	    7.37	80.64
Macoma tenta	     0.38	     0.05	   2.70	   0.59	    3.16	83.81
Mactridae	     0.28	     0.00	   1.85	   0.34	    2.17	85.97
Polymesoda sp.	     0.10	     0.00	   1.80	   0.19	    2.11	88.08
Polymesoda caroliniana	     0.32	     0.00	   1.39	   0.34	    1.63	89.71
Tagelus plebeius	     0.25	     0.00	   1.25	   0.52	    1.46	91.17

Groups M02  &  M04
Average dissimilarity = 88.77

	Group M02	Group M04	       	       	        	     
Species	 Av.Abund	 Av.Abund	Av.Diss	Diss/SD	Contrib%	Cum.%
Mulinia lateralis	     4.29	     4.11	  28.97	   1.33	   32.64	32.64
Mytilopsis leucophaeata	     4.21	     0.05	  19.12	   0.93	   21.54	54.18
Rangia cuneata	     2.40	     0.00	  15.83	   0.99	   17.83	72.01
Tellina sp	     1.17	     0.11	   6.21	   0.89	    7.00	79.01
Macoma tenta	     0.38	     0.15	   2.95	   0.65	    3.33	82.33
Mactridae	     0.28	     0.00	   1.85	   0.33	    2.09	84.42
Polymesoda sp.	     0.10	     0.00	   1.84	   0.19	    2.08	86.50
Tagelus divisus	     0.06	     0.18	   1.64	   0.44	    1.85	88.34
Polymesoda caroliniana	     0.32	     0.00	   1.38	   0.34	    1.55	89.89
Tagelus plebeius	     0.25	     0.00	   1.23	   0.51	    1.39	91.28

Groups M03  &  M04
Average dissimilarity = 58.73

	Group M03	Group M04	       	       	        	     
Species	 Av.Abund	 Av.Abund	Av.Diss	Diss/SD	Contrib%	Cum.%
Mulinia lateralis	     2.92	     4.11	  39.23	   1.63	   66.79	66.79
Mytilopsis leucophaeata	     0.95	     0.05	   9.12	   0.53	   15.53	82.33
Tellina sp	     0.10	     0.11	   2.93	   0.47	    4.99	87.31
Tagelus divisus	     0.00	     0.18	   2.74	   0.42	    4.66	91.97

Groups M02  &  M05
Average dissimilarity = 93.07

	Group M02	Group M05	       	       	        	     
Species	 Av.Abund	 Av.Abund	Av.Diss	Diss/SD	Contrib%	Cum.%
Mulinia lateralis	     4.29	     1.50	  23.39	   1.18	   25.13	25.13
Mytilopsis leucophaeata	     4.21	     0.05	  21.96	   0.99	   23.59	48.72
Rangia cuneata	     2.40	     0.00	  18.92	   1.02	   20.33	69.05
Tellina sp	     1.17	     0.00	   7.01	   0.85	    7.54	76.59
Macoma tenta	     0.38	     0.04	   3.07	   0.57	    3.30	79.89
Polymesoda sp.	     0.10	     0.00	   2.51	   0.20	    2.70	82.59
Tagelus divisus	     0.06	     0.21	   2.29	   0.48	    2.46	85.05
Mactridae	     0.28	     0.00	   2.27	   0.33	    2.44	87.50
Polymesoda caroliniana	     0.32	     0.00	   1.54	   0.34	    1.65	89.15
Tagelus plebeius	     0.25	     0.00	   1.40	   0.52	    1.50	90.65

Groups M03  &  M05
Average dissimilarity = 63.52

	Group M03	Group M05	       	       	        	     
Species	 Av.Abund	 Av.Abund	Av.Diss	Diss/SD	Contrib%	Cum.%
Mulinia lateralis	     2.92	     1.50	  40.90	   1.62	   64.39	64.39
Mytilopsis leucophaeata	     0.95	     0.05	  11.82	   0.58	   18.61	83.01
Tagelus divisus	     0.00	     0.21	   4.26	   0.49	    6.71	89.72
Tellina sp	     0.10	     0.00	   2.73	   0.35	    4.29	94.01

Groups M04  &  M05
Average dissimilarity = 63.81

	Group M04	Group M05	       	       	        	     
Species	 Av.Abund	 Av.Abund	Av.Diss	Diss/SD	Contrib%	Cum.%
Mulinia lateralis	     4.11	     1.50	  45.71	   1.75	   71.63	71.63
Tagelus divisus	     0.18	     0.21	   6.73	   0.64	   10.55	82.19
Mytilopsis leucophaeata	     0.05	     0.05	   2.40	   0.33	    3.77	85.95
Tellina sp	     0.11	     0.00	   2.17	   0.37	    3.40	89.36
Macoma tenta	     0.15	     0.04	   2.07	   0.50	    3.24	92.60

Groups M02  &  M06
Average dissimilarity = 91.07

	Group M02	Group M06	       	       	        	     
Species	 Av.Abund	 Av.Abund	Av.Diss	Diss/SD	Contrib%	Cum.%
Mulinia lateralis	     4.29	     1.51	  18.59	   1.08	   20.41	20.41
Mytilopsis leucophaeata	     4.21	     0.06	  18.14	   0.92	   19.92	40.33
Rangia cuneata	     2.40	     0.00	  14.62	   1.03	   16.05	56.39
Tellina sp	     1.17	     0.34	   6.18	   1.03	    6.79	63.17
Tagelus divisus	     0.06	     0.83	   4.86	   0.77	    5.33	68.51
Macoma tenta	     0.38	     0.29	   3.42	   0.73	    3.76	72.26
Abra aequalis	     0.00	     0.34	   2.58	   0.63	    2.83	75.10
Amygdalum papyrium	     0.28	     0.19	   1.93	   0.54	    2.11	77.21
Mactridae	     0.28	     0.00	   1.68	   0.34	    1.84	79.05
Polymesoda sp.	     0.10	     0.00	   1.53	   0.19	    1.67	80.73
Lyonsia floridana	     0.23	     0.11	   1.46	   0.50	    1.60	82.33
Chione sp.	     0.00	     0.18	   1.42	   0.51	    1.55	83.88
Polymesoda caroliniana	     0.32	     0.00	   1.33	   0.34	    1.46	85.34
Lucina sp.	     0.06	     0.13	   1.24	   0.45	    1.36	86.70
Tagelus plebeius	     0.25	     0.00	   1.18	   0.51	    1.30	88.00
Parvilucina multilineata	     0.02	     0.16	   1.11	   0.51	    1.22	89.22
Corbula sp.	     0.00	     0.17	   0.95	   0.36	    1.04	90.26

Groups M03  &  M06
Average dissimilarity = 76.69

	Group M03	Group M06	       	       	        	     
Species	 Av.Abund	 Av.Abund	Av.Diss	Diss/SD	Contrib%	Cum.%
Mulinia lateralis	     2.92	     1.51	  27.30	   1.49	   35.60	35.60
Mytilopsis leucophaeata	     0.95	     0.06	   8.34	   0.54	   10.87	46.47
Tagelus divisus	     0.00	     0.83	   8.16	   0.93	   10.64	57.12
Abra aequalis	     0.00	     0.34	   4.86	   0.67	    6.34	63.46
Tellina sp	     0.10	     0.34	   4.66	   0.77	    6.08	69.54
Macoma tenta	     0.05	     0.29	   3.56	   0.62	    4.64	74.18
Chione sp.	     0.00	     0.18	   2.59	   0.57	    3.38	77.56
Corbula sp.	     0.05	     0.17	   2.12	   0.43	    2.77	80.33
Parvilucina multilineata	     0.00	     0.16	   1.88	   0.56	    2.45	82.78
Amygdalum papyrium	     0.00	     0.19	   1.86	   0.42	    2.42	85.20
Caryocorbula sp.	     0.00	     0.14	   1.47	   0.45	    1.92	87.12
Lucina sp.	     0.00	     0.13	   1.44	   0.38	    1.87	88.99
Lucinoma filosa	     0.00	     0.10	   1.11	   0.25	    1.44	90.43

Groups M04  &  M06
Average dissimilarity = 75.30

	Group M04	Group M06	       	       	        	     
Species	 Av.Abund	 Av.Abund	Av.Diss	Diss/SD	Contrib%	Cum.%
Mulinia lateralis	     4.11	     1.51	  31.43	   1.39	   41.74	41.74
Tagelus divisus	     0.18	     0.83	   8.27	   0.94	   10.98	52.72
Abra aequalis	     0.00	     0.34	   4.96	   0.66	    6.59	59.31
Tellina sp	     0.11	     0.34	   4.64	   0.76	    6.17	65.48
Macoma tenta	     0.15	     0.29	   3.93	   0.67	    5.22	70.70
Chione sp.	     0.00	     0.18	   2.63	   0.56	    3.49	74.19
Parvilucina multilineata	     0.03	     0.16	   1.98	   0.57	    2.63	76.82
Amygdalum papyrium	     0.00	     0.19	   1.86	   0.42	    2.47	79.28
Corbula sp.	     0.00	     0.17	   1.55	   0.39	    2.06	81.34
Caryocorbula sp.	     0.00	     0.14	   1.48	   0.44	    1.96	83.30
Lucina sp.	     0.00	     0.13	   1.44	   0.38	    1.91	85.22
Lucinoma filosa	     0.00	     0.10	   1.13	   0.24	    1.50	86.71
Mytilopsis leucophaeata	     0.05	     0.06	   1.13	   0.30	    1.50	88.21
Corbicula sp.	     0.06	     0.00	   1.03	   0.20	    1.36	89.57
Lyonsia floridana	     0.00	     0.11	   0.92	   0.27	    1.22	90.79

Groups M05  &  M06
Average dissimilarity = 75.48

	Group M05	Group M06	       	       	        	     
Species	 Av.Abund	 Av.Abund	Av.Diss	Diss/SD	Contrib%	Cum.%
Mulinia lateralis	     1.50	     1.51	  21.39	   1.21	   28.35	28.35
Tagelus divisus	     0.21	     0.83	  10.47	   1.04	   13.87	42.21
Abra aequalis	     0.00	     0.34	   6.72	   0.68	    8.90	51.11
Tellina sp	     0.00	     0.34	   5.38	   0.74	    7.13	58.24
Macoma tenta	     0.04	     0.29	   4.38	   0.60	    5.80	64.04
Chione sp.	     0.00	     0.18	   3.45	   0.59	    4.58	68.62
Parvilucina multilineata	     0.03	     0.16	   2.74	   0.55	    3.63	72.24
Amygdalum papyrium	     0.00	     0.19	   2.29	   0.43	    3.04	75.28
Corbula sp.	     0.00	     0.17	   1.91	   0.40	    2.53	77.81
Mytilopsis leucophaeata	     0.05	     0.06	   1.87	   0.32	    2.48	80.29
Caryocorbula sp.	     0.00	     0.14	   1.85	   0.45	    2.45	82.74
Lucina sp.	     0.00	     0.13	   1.81	   0.39	    2.40	85.13
Lucinoma filosa	     0.00	     0.10	   1.51	   0.24	    2.00	87.13
Lyonsia floridana	     0.00	     0.11	   1.11	   0.28	    1.47	88.60
Tellinidae	     0.04	     0.02	   1.09	   0.26	    1.44	90.04

Groups M02  &  M07
Average dissimilarity = 90.74

	Group M02	Group M07	       	       	        	     
Species	 Av.Abund	 Av.Abund	Av.Diss	Diss/SD	Contrib%	Cum.%
Mytilopsis leucophaeata	     4.21	     0.07	  11.42	   0.80	   12.59	12.59
Mulinia lateralis	     4.29	     0.68	  10.54	   0.75	   11.61	24.20
Rangia cuneata	     2.40	     0.00	   8.24	   1.05	    9.08	33.28
Tellina sp	     1.17	     2.55	   7.50	   1.24	    8.26	41.54
Parvilucina multilineata	     0.02	     1.77	   6.28	   1.08	    6.92	48.46
Macoma tenta	     0.38	     1.55	   5.14	   0.82	    5.67	54.13
Abra aequalis	     0.00	     1.30	   4.40	   1.90	    4.84	58.97
Nucula proxima	     0.00	     0.87	   3.00	   1.11	    3.31	62.28
Tagelus divisus	     0.06	     0.82	   2.90	   0.75	    3.20	65.47
Chione sp.	     0.00	     0.85	   2.90	   0.94	    3.19	68.67
Lyonsia floridana	     0.23	     0.65	   2.18	   1.07	    2.41	71.07
Lucina sp.	     0.06	     0.45	   1.70	   0.66	    1.88	72.95
Divaricella quadrisulcata	     0.00	     0.46	   1.61	   0.50	    1.77	74.72
Amygdalum papyrium	     0.28	     0.33	   1.49	   0.65	    1.64	76.37
Anadara sp.	     0.00	     0.40	   1.40	   0.71	    1.54	77.91
Mactridae	     0.28	     0.16	   1.23	   0.53	    1.35	79.26
Bivalvia	     0.02	     0.28	   0.97	   0.45	    1.07	80.32
Caryocorbula sp.	     0.00	     0.32	   0.95	   0.64	    1.05	81.37
Crassinella lunulata	     0.00	     0.28	   0.90	   0.56	    0.99	82.36
Tellina versicolor	     0.00	     0.21	   0.88	   0.28	    0.97	83.33
Polymesoda caroliniana	     0.32	     0.00	   0.88	   0.35	    0.97	84.30
Asthenothaerus hemphilli	     0.00	     0.21	   0.83	   0.46	    0.91	85.21
Corbula sp.	     0.00	     0.23	   0.78	   0.60	    0.86	86.07
Tagelus plebeius	     0.25	     0.02	   0.77	   0.52	    0.85	86.92
Timoclea sp.	     0.00	     0.25	   0.76	   0.45	    0.84	87.76
Sphenia sp.	     0.00	     0.20	   0.73	   0.46	    0.81	88.57
Polymesoda sp.	     0.10	     0.02	   0.64	   0.22	    0.70	89.27
Crassinella sp.	     0.00	     0.21	   0.63	   0.44	    0.69	89.97
Lucinidae	     0.00	     0.16	   0.59	   0.27	    0.65	90.62

Groups M03  &  M07
Average dissimilarity = 93.50

	Group M03	Group M07	       	       	        	     
Species	 Av.Abund	 Av.Abund	Av.Diss	Diss/SD	Contrib%	Cum.%
Mulinia lateralis	     2.92	     0.68	  12.42	   1.39	   13.29	13.29
Tellina sp	     0.10	     2.55	  11.29	   1.46	   12.07	25.36
Parvilucina multilineata	     0.00	     1.77	   8.73	   1.17	    9.33	34.70
Macoma tenta	     0.05	     1.55	   6.85	   0.82	    7.33	42.03
Abra aequalis	     0.00	     1.30	   6.01	   2.43	    6.43	48.46
Mytilopsis leucophaeata	     0.95	     0.07	   4.13	   0.50	    4.42	52.88
Tagelus divisus	     0.00	     0.82	   4.13	   0.78	    4.42	57.29
Nucula proxima	     0.00	     0.87	   4.13	   1.21	    4.41	61.71
Chione sp.	     0.00	     0.85	   3.98	   1.00	    4.26	65.97
Lyonsia floridana	     0.00	     0.65	   2.92	   1.15	    3.12	69.09
Lucina sp.	     0.00	     0.45	   2.28	   0.63	    2.44	71.52
Divaricella quadrisulcata	     0.00	     0.46	   2.24	   0.51	    2.40	73.92
Anadara sp.	     0.00	     0.40	   1.93	   0.75	    2.06	75.98
Amygdalum papyrium	     0.00	     0.33	   1.37	   0.54	    1.47	77.45
Tellina versicolor	     0.00	     0.21	   1.27	   0.30	    1.36	78.81
Caryocorbula sp.	     0.00	     0.32	   1.26	   0.67	    1.35	80.15
Corbula sp.	     0.05	     0.23	   1.25	   0.63	    1.34	81.49
Crassinella lunulata	     0.00	     0.28	   1.21	   0.58	    1.29	82.79
Asthenothaerus hemphilli	     0.00	     0.21	   1.17	   0.48	    1.25	84.04
Bivalvia	     0.00	     0.28	   1.14	   0.42	    1.22	85.26
Sphenia sp.	     0.00	     0.20	   1.04	   0.48	    1.11	86.37
Timoclea sp.	     0.00	     0.25	   1.00	   0.46	    1.07	87.44
Lucinidae	     0.00	     0.16	   0.85	   0.27	    0.91	88.35
Crassinella sp.	     0.00	     0.21	   0.83	   0.45	    0.89	89.24
Parvilucina sp.	     0.00	     0.16	   0.65	   0.19	    0.70	89.93
Mactridae	     0.00	     0.16	   0.65	   0.44	    0.69	90.62

Groups M04  &  M07
Average dissimilarity = 92.31

	Group M04	Group M07	       	       	        	     
Species	 Av.Abund	 Av.Abund	Av.Diss	Diss/SD	Contrib%	Cum.%
Mulinia lateralis	     4.11	     0.68	  15.54	   1.08	   16.84	16.84
Tellina sp	     0.11	     2.55	  11.15	   1.42	   12.08	28.92
Parvilucina multilineata	     0.03	     1.77	   8.57	   1.15	    9.29	38.20
Macoma tenta	     0.15	     1.55	   6.72	   0.82	    7.28	45.48
Abra aequalis	     0.00	     1.30	   5.95	   2.32	    6.44	51.93
Nucula proxima	     0.00	     0.87	   4.08	   1.19	    4.42	56.35
Tagelus divisus	     0.18	     0.82	   3.98	   0.78	    4.32	60.66
Chione sp.	     0.00	     0.85	   3.94	   0.98	    4.27	64.93
Lyonsia floridana	     0.00	     0.65	   2.88	   1.13	    3.12	68.05
Lucina sp.	     0.00	     0.45	   2.25	   0.62	    2.44	70.50
Divaricella quadrisulcata	     0.00	     0.46	   2.22	   0.51	    2.40	72.90
Anadara sp.	     0.00	     0.40	   1.91	   0.74	    2.07	74.96
Amygdalum papyrium	     0.00	     0.33	   1.36	   0.53	    1.47	76.43
Tellina versicolor	     0.00	     0.21	   1.25	   0.30	    1.36	77.79
Caryocorbula sp.	     0.00	     0.32	   1.24	   0.66	    1.35	79.14
Crassinella lunulata	     0.00	     0.28	   1.19	   0.58	    1.29	80.43
Asthenothaerus hemphilli	     0.00	     0.21	   1.16	   0.48	    1.25	81.69
Bivalvia	     0.00	     0.28	   1.13	   0.42	    1.22	82.91
Corbula sp.	     0.00	     0.23	   1.05	   0.62	    1.13	84.04
Sphenia sp.	     0.00	     0.20	   1.03	   0.48	    1.11	85.16
Timoclea sp.	     0.00	     0.25	   0.99	   0.46	    1.07	86.23
Lucinidae	     0.00	     0.16	   0.84	   0.27	    0.91	87.14
Crassinella sp.	     0.00	     0.21	   0.82	   0.45	    0.89	88.03
Parvilucina sp.	     0.00	     0.16	   0.64	   0.19	    0.70	88.72
Mactridae	     0.00	     0.16	   0.64	   0.44	    0.69	89.42
Divaricella dentata	     0.00	     0.09	   0.62	   0.19	    0.67	90.09

Groups M05  &  M07
Average dissimilarity = 93.43

	Group M05	Group M07	       	       	        	     
Species	 Av.Abund	 Av.Abund	Av.Diss	Diss/SD	Contrib%	Cum.%
Tellina sp	     0.00	     2.55	  12.82	   1.48	   13.72	13.72
Parvilucina multilineata	     0.03	     1.77	   9.65	   1.18	   10.33	24.06
Macoma tenta	     0.04	     1.55	   7.59	   0.83	    8.13	32.18
Mulinia lateralis	     1.50	     0.68	   7.10	   1.01	    7.60	39.78
Abra aequalis	     0.00	     1.30	   6.68	   2.55	    7.15	46.93
Nucula proxima	     0.00	     0.87	   4.59	   1.23	    4.91	51.84
Tagelus divisus	     0.21	     0.82	   4.51	   0.79	    4.83	56.68
Chione sp.	     0.00	     0.85	   4.44	   0.99	    4.75	61.42
Lyonsia floridana	     0.00	     0.65	   3.22	   1.16	    3.45	64.87
Lucina sp.	     0.00	     0.45	   2.56	   0.63	    2.74	67.62
Divaricella quadrisulcata	     0.00	     0.46	   2.51	   0.51	    2.68	70.30
Anadara sp.	     0.00	     0.40	   2.15	   0.76	    2.30	72.60
Amygdalum papyrium	     0.00	     0.33	   1.52	   0.54	    1.62	74.23
Tellina versicolor	     0.00	     0.21	   1.44	   0.30	    1.54	75.77
Caryocorbula sp.	     0.00	     0.32	   1.38	   0.67	    1.47	77.24
Crassinella lunulata	     0.00	     0.28	   1.33	   0.58	    1.42	78.66
Asthenothaerus hemphilli	     0.00	     0.21	   1.32	   0.49	    1.41	80.07
Bivalvia	     0.00	     0.28	   1.25	   0.42	    1.33	81.40
Sphenia sp.	     0.00	     0.20	   1.17	   0.48	    1.25	82.66
Corbula sp.	     0.00	     0.23	   1.17	   0.62	    1.25	83.91
Timoclea sp.	     0.00	     0.25	   1.09	   0.46	    1.16	85.07
Lucinidae	     0.00	     0.16	   0.97	   0.27	    1.03	86.11
Crassinella sp.	     0.00	     0.21	   0.90	   0.46	    0.97	87.08
Divaricella dentata	     0.00	     0.09	   0.73	   0.19	    0.78	87.85
Mytilopsis leucophaeata	     0.05	     0.07	   0.72	   0.42	    0.77	88.62
Parvilucina sp.	     0.00	     0.16	   0.71	   0.19	    0.76	89.38
Mactridae	     0.00	     0.16	   0.71	   0.44	    0.76	90.13

Groups M06  &  M07
Average dissimilarity = 81.15

	Group M06	Group M07	       	       	        	     
Species	 Av.Abund	 Av.Abund	Av.Diss	Diss/SD	Contrib%	Cum.%
Tellina sp	     0.34	     2.55	  10.14	   1.43	   12.49	12.49
Parvilucina multilineata	     0.16	     1.77	   7.87	   1.16	    9.70	22.19
Macoma tenta	     0.29	     1.55	   6.46	   0.85	    7.96	30.16
Mulinia lateralis	     1.51	     0.68	   5.71	   0.91	    7.04	37.19
Tagelus divisus	     0.83	     0.82	   4.47	   0.87	    5.51	42.70
Abra aequalis	     0.34	     1.30	   4.39	   1.64	    5.41	48.11
Nucula proxima	     0.08	     0.87	   3.90	   1.21	    4.80	52.91
Chione sp.	     0.18	     0.85	   3.63	   1.07	    4.47	57.38
Lyonsia floridana	     0.11	     0.65	   2.81	   1.15	    3.47	60.85
Lucina sp.	     0.13	     0.45	   2.35	   0.72	    2.90	63.75
Divaricella quadrisulcata	     0.00	     0.46	   2.12	   0.52	    2.61	66.36
Anadara sp.	     0.05	     0.40	   1.88	   0.78	    2.32	68.68
Amygdalum papyrium	     0.19	     0.33	   1.81	   0.68	    2.24	70.92
Caryocorbula sp.	     0.14	     0.32	   1.53	   0.81	    1.88	72.80
Corbula sp.	     0.17	     0.23	   1.45	   0.70	    1.79	74.59
Bivalvia	     0.05	     0.28	   1.29	   0.49	    1.58	76.18
Tellina versicolor	     0.00	     0.21	   1.20	   0.30	    1.47	77.65
Crassinella lunulata	     0.02	     0.28	   1.19	   0.60	    1.46	79.11
Asthenothaerus hemphilli	     0.02	     0.21	   1.16	   0.51	    1.43	80.54
Sphenia sp.	     0.07	     0.20	   1.13	   0.56	    1.40	81.94
Timoclea sp.	     0.00	     0.25	   0.96	   0.46	    1.18	83.12
Crassinella sp.	     0.02	     0.21	   0.86	   0.49	    1.06	84.19
Lucinidae	     0.00	     0.16	   0.80	   0.27	    0.98	85.17
Parvilucina sp.	     0.03	     0.16	   0.72	   0.22	    0.89	86.06
Mytilopsis leucophaeata	     0.06	     0.07	   0.63	   0.35	    0.78	86.84
Mactridae	     0.00	     0.16	   0.62	   0.44	    0.77	87.60
Divaricella dentata	     0.00	     0.09	   0.59	   0.19	    0.72	88.32
Lucinoma filosa	     0.10	     0.06	   0.57	   0.39	    0.71	89.03
Tagelus sp.	     0.07	     0.04	   0.48	   0.24	    0.59	89.62
Timoclea grus	     0.00	     0.11	   0.47	   0.33	    0.58	90.20

Groups M02  &  M08
Average dissimilarity = 91.95

	Group M02	Group M08	       	       	        	     
Species	 Av.Abund	 Av.Abund	Av.Diss	Diss/SD	Contrib%	Cum.%
Mytilopsis leucophaeata	     4.21	     0.00	  11.89	   0.81	   12.93	12.93
Mulinia lateralis	     4.29	     1.09	  11.36	   0.85	   12.36	25.29
Rangia cuneata	     2.40	     0.00	   8.53	   1.05	    9.28	34.57
Abra aequalis	     0.00	     2.05	   7.55	   1.71	    8.21	42.78
Tagelus divisus	     0.06	     1.96	   6.79	   1.22	    7.38	50.16
Tellina sp	     1.17	     1.03	   4.52	   1.13	    4.92	55.08
Parvilucina multilineata	     0.02	     1.08	   3.86	   1.34	    4.20	59.28
Nucula proxima	     0.00	     1.08	   3.75	   1.19	    4.08	63.36
Macoma tenta	     0.38	     0.97	   3.56	   1.03	    3.87	67.23
Chione sp.	     0.00	     0.96	   3.48	   1.26	    3.79	71.02
Caryocorbula sp.	     0.00	     0.79	   2.71	   0.78	    2.95	73.97
Corbula sp.	     0.00	     0.69	   2.43	   0.97	    2.65	76.62
Lyonsia floridana	     0.23	     0.48	   1.92	   0.88	    2.09	78.70
Lucina sp.	     0.06	     0.34	   1.61	   0.55	    1.75	80.45
Lucinoma filosa	     0.00	     0.39	   1.53	   0.58	    1.67	82.12
Amygdalum papyrium	     0.28	     0.21	   1.23	   0.62	    1.34	83.46
Mactridae	     0.28	     0.09	   1.14	   0.49	    1.24	84.70
Polymesoda caroliniana	     0.32	     0.00	   0.91	   0.35	    0.98	85.69
Tellinidae	     0.12	     0.16	   0.91	   0.45	    0.98	86.67
Asthenothaerus hemphilli	     0.00	     0.25	   0.90	   0.46	    0.98	87.66
Bivalvia	     0.02	     0.18	   0.81	   0.50	    0.88	88.53
Tagelus plebeius	     0.25	     0.00	   0.77	   0.50	    0.84	89.38
Nuculana acuta	     0.00	     0.20	   0.68	   0.44	    0.74	90.11

Groups M03  &  M08
Average dissimilarity = 90.01

	Group M03	Group M08	       	       	        	     
Species	 Av.Abund	 Av.Abund	Av.Diss	Diss/SD	Contrib%	Cum.%
Mulinia lateralis	     2.92	     1.09	  11.76	   1.32	   13.07	13.07
Abra aequalis	     0.00	     2.05	  10.56	   2.02	   11.73	24.80
Tagelus divisus	     0.00	     1.96	   9.60	   1.40	   10.66	35.46
Parvilucina multilineata	     0.00	     1.08	   5.36	   1.52	    5.95	41.41
Nucula proxima	     0.00	     1.08	   5.13	   1.32	    5.70	47.11
Tellina sp	     0.10	     1.03	   5.04	   1.02	    5.59	52.70
Chione sp.	     0.00	     0.96	   4.86	   1.39	    5.40	58.11
Macoma tenta	     0.05	     0.97	   4.55	   1.00	    5.06	63.16
Mytilopsis leucophaeata	     0.95	     0.00	   4.15	   0.48	    4.61	67.77
Caryocorbula sp.	     0.00	     0.79	   3.71	   0.83	    4.12	71.89
Corbula sp.	     0.05	     0.69	   3.38	   1.05	    3.75	75.65
Lyonsia floridana	     0.00	     0.48	   2.44	   0.86	    2.71	78.36
Lucinoma filosa	     0.00	     0.39	   2.23	   0.60	    2.47	80.83
Lucina sp.	     0.00	     0.34	   2.21	   0.53	    2.46	83.29
Asthenothaerus hemphilli	     0.00	     0.25	   1.26	   0.48	    1.40	84.69
Bivalvia	     0.00	     0.18	   1.02	   0.47	    1.13	85.82
Amygdalum papyrium	     0.00	     0.21	   0.98	   0.52	    1.08	86.90
Nuculana acuta	     0.00	     0.20	   0.92	   0.46	    1.02	87.92
Corbula contracta	     0.00	     0.22	   0.85	   0.34	    0.95	88.87
Tellinidae	     0.00	     0.16	   0.78	   0.42	    0.87	89.74
Anadara floridana	     0.00	     0.12	   0.75	   0.41	    0.83	90.57

Groups M04  &  M08
Average dissimilarity = 88.53

	Group M04	Group M08	       	       	        	     
Species	 Av.Abund	 Av.Abund	Av.Diss	Diss/SD	Contrib%	Cum.%
Mulinia lateralis	     4.11	     1.09	  15.14	   1.05	   17.10	17.10
Abra aequalis	     0.00	     2.05	  10.45	   1.93	   11.80	28.90
Tagelus divisus	     0.18	     1.96	   8.96	   1.32	   10.12	39.02
Parvilucina multilineata	     0.03	     1.08	   5.24	   1.47	    5.92	44.94
Nucula proxima	     0.00	     1.08	   5.07	   1.29	    5.73	50.66
Tellina sp	     0.11	     1.03	   4.98	   1.00	    5.62	56.29
Chione sp.	     0.00	     0.96	   4.81	   1.35	    5.43	61.72
Macoma tenta	     0.15	     0.97	   4.45	   1.00	    5.03	66.75
Caryocorbula sp.	     0.00	     0.79	   3.67	   0.82	    4.14	70.89
Corbula sp.	     0.00	     0.69	   3.29	   1.04	    3.72	74.61
Lyonsia floridana	     0.00	     0.48	   2.41	   0.84	    2.73	77.34
Lucinoma filosa	     0.00	     0.39	   2.21	   0.58	    2.49	79.83
Lucina sp.	     0.00	     0.34	   2.19	   0.52	    2.48	82.31
Asthenothaerus hemphilli	     0.00	     0.25	   1.24	   0.47	    1.40	83.71
Bivalvia	     0.00	     0.18	   1.01	   0.47	    1.14	84.85
Amygdalum papyrium	     0.00	     0.21	   0.97	   0.51	    1.09	85.94
Nuculana acuta	     0.00	     0.20	   0.91	   0.46	    1.02	86.97
Corbula contracta	     0.00	     0.22	   0.84	   0.34	    0.95	87.92
Tellinidae	     0.00	     0.16	   0.77	   0.41	    0.87	88.80
Anadara floridana	     0.00	     0.12	   0.74	   0.41	    0.84	89.63
Anadara sp.	     0.00	     0.17	   0.73	   0.43	    0.83	90.46

Groups M05  &  M08
Average dissimilarity = 89.82

	Group M05	Group M08	       	       	        	     
Species	 Av.Abund	 Av.Abund	Av.Diss	Diss/SD	Contrib%	Cum.%
Abra aequalis	     0.00	     2.05	  11.85	   2.03	   13.19	13.19
Tagelus divisus	     0.21	     1.96	  10.00	   1.36	   11.14	24.33
Mulinia lateralis	     1.50	     1.09	   7.20	   1.06	    8.02	32.35
Parvilucina multilineata	     0.03	     1.08	   5.88	   1.54	    6.55	38.90
Tellina sp	     0.00	     1.03	   5.73	   1.00	    6.38	45.27
Nucula proxima	     0.00	     1.08	   5.69	   1.34	    6.33	51.61
Chione sp.	     0.00	     0.96	   5.45	   1.38	    6.07	57.67
Macoma tenta	     0.04	     0.97	   5.12	   1.02	    5.70	63.38
Caryocorbula sp.	     0.00	     0.79	   4.11	   0.83	    4.58	67.96
Corbula sp.	     0.00	     0.69	   3.69	   1.06	    4.11	72.07
Lyonsia floridana	     0.00	     0.48	   2.75	   0.85	    3.06	75.12
Lucinoma filosa	     0.00	     0.39	   2.56	   0.58	    2.85	77.97
Lucina sp.	     0.00	     0.34	   2.54	   0.53	    2.83	80.81
Asthenothaerus hemphilli	     0.00	     0.25	   1.41	   0.48	    1.56	82.37
Bivalvia	     0.00	     0.18	   1.15	   0.47	    1.28	83.66
Amygdalum papyrium	     0.00	     0.21	   1.10	   0.51	    1.23	84.88
Tellinidae	     0.04	     0.16	   1.04	   0.47	    1.16	86.04
Nuculana acuta	     0.00	     0.20	   1.01	   0.46	    1.13	87.17
Corbula contracta	     0.00	     0.22	   0.93	   0.34	    1.03	88.20
Anadara floridana	     0.00	     0.12	   0.86	   0.41	    0.95	89.16
Anadara sp.	     0.00	     0.17	   0.81	   0.43	    0.90	90.06

Groups M06  &  M08
Average dissimilarity = 76.14

	Group M06	Group M08	       	       	        	     
Species	 Av.Abund	 Av.Abund	Av.Diss	Diss/SD	Contrib%	Cum.%
Abra aequalis	     0.34	     2.05	   8.46	   1.73	   11.11	11.11
Tagelus divisus	     0.83	     1.96	   8.00	   1.29	   10.51	21.62
Mulinia lateralis	     1.51	     1.09	   5.72	   0.94	    7.52	29.14
Nucula proxima	     0.08	     1.08	   4.82	   1.30	    6.33	35.46
Parvilucina multilineata	     0.16	     1.08	   4.71	   1.47	    6.18	41.64
Tellina sp	     0.34	     1.03	   4.69	   1.07	    6.16	47.80
Macoma tenta	     0.29	     0.97	   4.35	   1.08	    5.71	53.51
Chione sp.	     0.18	     0.96	   4.12	   1.34	    5.41	58.92
Caryocorbula sp.	     0.14	     0.79	   3.64	   0.91	    4.78	63.69
Corbula sp.	     0.17	     0.69	   3.29	   1.10	    4.31	68.01
Lyonsia floridana	     0.11	     0.48	   2.43	   0.90	    3.19	71.20
Lucina sp.	     0.13	     0.34	   2.32	   0.61	    3.05	74.25
Lucinoma filosa	     0.10	     0.39	   2.22	   0.66	    2.92	77.17
Amygdalum papyrium	     0.19	     0.21	   1.50	   0.66	    1.97	79.14
Asthenothaerus hemphilli	     0.02	     0.25	   1.25	   0.51	    1.65	80.79
Bivalvia	     0.05	     0.18	   1.13	   0.54	    1.48	82.27
Nuculana acuta	     0.00	     0.20	   0.87	   0.46	    1.15	83.42
Anadara sp.	     0.05	     0.17	   0.87	   0.50	    1.15	84.56
Macoma sp.	     0.06	     0.13	   0.83	   0.34	    1.09	85.65
Tellinidae	     0.02	     0.16	   0.82	   0.45	    1.08	86.74
Corbula contracta	     0.00	     0.22	   0.82	   0.34	    1.08	87.82
Anadara floridana	     0.00	     0.12	   0.70	   0.41	    0.92	88.74
Corbula chittyana	     0.00	     0.12	   0.59	   0.20	    0.77	89.51
Sphenia sp.	     0.07	     0.09	   0.55	   0.50	    0.72	90.24

Groups M07  &  M08
Average dissimilarity = 64.58

	Group M07	Group M08	       	       	        	     
Species	 Av.Abund	 Av.Abund	Av.Diss	Diss/SD	Contrib%	Cum.%
Tellina sp	     2.55	     1.03	   6.36	   1.37	    9.85	 9.85
Tagelus divisus	     0.82	     1.96	   4.93	   1.22	    7.64	17.49
Macoma tenta	     1.55	     0.97	   4.71	   0.96	    7.30	24.79
Parvilucina multilineata	     1.77	     1.08	   4.31	   1.17	    6.67	31.46
Abra aequalis	     1.30	     2.05	   3.57	   1.20	    5.54	37.00
Mulinia lateralis	     0.68	     1.09	   2.83	   1.28	    4.39	41.38
Nucula proxima	     0.87	     1.08	   2.77	   1.21	    4.29	45.67
Chione sp.	     0.85	     0.96	   2.61	   1.22	    4.04	49.72
Caryocorbula sp.	     0.32	     0.79	   2.43	   0.95	    3.77	53.48
Corbula sp.	     0.23	     0.69	   2.03	   1.10	    3.14	56.62
Lucina sp.	     0.45	     0.34	   1.93	   0.82	    2.99	59.61
Lyonsia floridana	     0.65	     0.48	   1.87	   1.20	    2.90	62.52
Divaricella quadrisulcata	     0.46	     0.11	   1.55	   0.61	    2.40	64.91
Anadara sp.	     0.40	     0.17	   1.39	   0.84	    2.15	67.06
Lucinoma filosa	     0.06	     0.39	   1.30	   0.69	    2.01	69.07
Amygdalum papyrium	     0.33	     0.21	   1.23	   0.74	    1.91	70.98
Asthenothaerus hemphilli	     0.21	     0.25	   1.20	   0.69	    1.86	72.84
Bivalvia	     0.28	     0.18	   1.19	   0.62	    1.84	74.68
Tellina versicolor	     0.21	     0.06	   0.90	   0.36	    1.40	76.08
Crassinella lunulata	     0.28	     0.06	   0.87	   0.63	    1.34	77.43
Timoclea sp.	     0.25	     0.06	   0.80	   0.53	    1.23	78.66
Sphenia sp.	     0.20	     0.09	   0.77	   0.59	    1.19	79.84
Crassinella sp.	     0.21	     0.08	   0.73	   0.57	    1.13	80.97
Corbula contracta	     0.05	     0.22	   0.69	   0.40	    1.08	82.05
Mactridae	     0.16	     0.09	   0.63	   0.60	    0.97	83.02
Nuculana acuta	     0.02	     0.20	   0.61	   0.48	    0.95	83.96
Lucinidae	     0.16	     0.03	   0.58	   0.33	    0.90	84.87
Tellinidae	     0.04	     0.16	   0.56	   0.48	    0.87	85.74
Anadara floridana	     0.08	     0.12	   0.56	   0.52	    0.87	86.61
Parvilucina sp.	     0.16	     0.02	   0.50	   0.22	    0.78	87.39
Corbula chittyana	     0.00	     0.12	   0.38	   0.20	    0.59	87.98
Macoma sp.	     0.00	     0.13	   0.38	   0.29	    0.58	88.56
Sphenia antillensis	     0.09	     0.02	   0.36	   0.39	    0.56	89.12
Veneridae	     0.10	     0.03	   0.36	   0.44	    0.56	89.69
Divaricella dentata	     0.09	     0.00	   0.35	   0.19	    0.54	90.22

Groups M02  &  M09
Average dissimilarity = 93.21

	Group M02	Group M09	       	       	        	     
Species	 Av.Abund	 Av.Abund	Av.Diss	Diss/SD	Contrib%	Cum.%
Mytilopsis leucophaeata	     4.21	     0.00	  13.29	   0.84	   14.25	14.25
Mulinia lateralis	     4.29	     0.32	  11.46	   0.73	   12.30	26.55
Rangia cuneata	     2.40	     0.00	   9.71	   1.09	   10.42	36.97
Chione sp.	     0.00	     1.71	   7.50	   1.43	    8.05	45.02
Tellina sp	     1.17	     1.06	   5.25	   1.20	    5.63	50.66
Tagelus divisus	     0.06	     1.05	   4.46	   0.88	    4.78	55.44
Nucula proxima	     0.00	     0.97	   4.31	   1.33	    4.62	60.06
Abra aequalis	     0.00	     0.87	   3.76	   1.20	    4.03	64.09
Macoma tenta	     0.38	     0.70	   3.43	   0.94	    3.68	67.77
Lucina sp.	     0.06	     0.59	   2.67	   0.71	    2.87	70.63
Parvilucina multilineata	     0.02	     0.62	   2.65	   0.80	    2.85	73.48
Corbula sp.	     0.00	     0.51	   2.27	   0.91	    2.43	75.91
Lyonsia floridana	     0.23	     0.44	   2.12	   0.91	    2.28	78.19
Chione cancellata	     0.00	     0.38	   1.77	   0.39	    1.90	80.08
Caryocorbula sp.	     0.00	     0.40	   1.75	   0.68	    1.88	81.96
Anadara sp.	     0.00	     0.31	   1.44	   0.58	    1.54	83.51
Mactridae	     0.28	     0.02	   1.12	   0.41	    1.20	84.70
Polymesoda caroliniana	     0.32	     0.00	   1.01	   0.35	    1.08	85.79
Amygdalum papyrium	     0.28	     0.04	   0.90	   0.45	    0.96	86.75
Tagelus plebeius	     0.25	     0.00	   0.87	   0.51	    0.93	87.68
Polymesoda sp.	     0.10	     0.00	   0.72	   0.20	    0.77	88.45
Bivalvia	     0.02	     0.11	   0.58	   0.45	    0.62	89.07
Tellinidae	     0.12	     0.03	   0.55	   0.29	    0.59	89.66
Sphenia sp.	     0.00	     0.13	   0.55	   0.46	    0.59	90.24

Groups M03  &  M09
Average dissimilarity = 95.04

	Group M03	Group M09	       	       	        	     
Species	 Av.Abund	 Av.Abund	Av.Diss	Diss/SD	Contrib%	Cum.%
Mulinia lateralis	     2.92	     0.32	  16.25	   1.56	   17.10	17.10
Chione sp.	     0.00	     1.71	  10.94	   1.72	   11.51	28.61
Tagelus divisus	     0.00	     1.05	   6.56	   0.98	    6.90	35.51
Tellina sp	     0.10	     1.06	   6.47	   1.14	    6.81	42.32
Nucula proxima	     0.00	     0.97	   6.31	   1.55	    6.64	48.97
Abra aequalis	     0.00	     0.87	   5.45	   1.39	    5.74	54.70
Mytilopsis leucophaeata	     0.95	     0.00	   4.81	   0.49	    5.07	59.77
Macoma tenta	     0.05	     0.70	   4.28	   0.88	    4.51	64.28
Parvilucina multilineata	     0.00	     0.62	   3.83	   0.87	    4.03	68.31
Lucina sp.	     0.00	     0.59	   3.83	   0.73	    4.03	72.34
Corbula sp.	     0.05	     0.51	   3.41	   1.03	    3.59	75.93
Lyonsia floridana	     0.00	     0.44	   2.80	   0.94	    2.95	78.87
Chione cancellata	     0.00	     0.38	   2.62	   0.41	    2.76	81.63
Caryocorbula sp.	     0.00	     0.40	   2.56	   0.74	    2.69	84.32
Anadara sp.	     0.00	     0.31	   2.14	   0.62	    2.25	86.58
Sphenia sp.	     0.00	     0.13	   0.79	   0.49	    0.84	87.41
Asthenothaerus hemphilli	     0.00	     0.12	   0.76	   0.36	    0.80	88.21
Anadara floridana	     0.00	     0.12	   0.70	   0.33	    0.73	88.94
Divaricella quadrisulcata	     0.00	     0.11	   0.66	   0.45	    0.69	89.64
Lucinisca nassula	     0.00	     0.10	   0.64	   0.44	    0.67	90.30

Groups M04  &  M09
Average dissimilarity = 93.43

	Group M04	Group M09	       	       	        	     
Species	 Av.Abund	 Av.Abund	Av.Diss	Diss/SD	Contrib%	Cum.%
Mulinia lateralis	     4.11	     0.32	  19.58	   1.20	   20.96	20.96
Chione sp.	     0.00	     1.71	  10.82	   1.65	   11.58	32.55
Tellina sp	     0.11	     1.06	   6.42	   1.10	    6.87	39.41
Tagelus divisus	     0.18	     1.05	   6.26	   0.97	    6.70	46.11
Nucula proxima	     0.00	     0.97	   6.25	   1.50	    6.69	52.80
Abra aequalis	     0.00	     0.87	   5.39	   1.35	    5.77	58.58
Macoma tenta	     0.15	     0.70	   4.24	   0.89	    4.53	63.11
Lucina sp.	     0.00	     0.59	   3.79	   0.71	    4.06	67.17
Parvilucina multilineata	     0.03	     0.62	   3.79	   0.86	    4.06	71.23
Corbula sp.	     0.00	     0.51	   3.29	   0.98	    3.52	74.75
Lyonsia floridana	     0.00	     0.44	   2.77	   0.92	    2.97	77.72
Chione cancellata	     0.00	     0.38	   2.60	   0.41	    2.78	80.50
Caryocorbula sp.	     0.00	     0.40	   2.53	   0.73	    2.71	83.21
Anadara sp.	     0.00	     0.31	   2.12	   0.61	    2.27	85.48
Sphenia sp.	     0.00	     0.13	   0.79	   0.48	    0.84	86.32
Asthenothaerus hemphilli	     0.00	     0.12	   0.75	   0.36	    0.81	87.13
Anadara floridana	     0.00	     0.12	   0.69	   0.33	    0.74	87.86
Divaricella quadrisulcata	     0.00	     0.11	   0.65	   0.44	    0.69	88.56
Lucinisca nassula	     0.00	     0.10	   0.63	   0.44	    0.67	89.23
Bivalvia	     0.00	     0.11	   0.62	   0.43	    0.66	89.90
Timoclea sp.	     0.00	     0.09	   0.57	   0.37	    0.61	90.51

Groups M05  &  M09
Average dissimilarity = 94.22

	Group M05	Group M09	       	       	        	     
Species	 Av.Abund	 Av.Abund	Av.Diss	Diss/SD	Contrib%	Cum.%
Chione sp.	     0.00	     1.71	  12.49	   1.76	   13.26	13.26
Mulinia lateralis	     1.50	     0.32	   9.15	   1.07	    9.71	22.97
Tellina sp	     0.00	     1.06	   7.66	   1.15	    8.13	31.10
Nucula proxima	     0.00	     0.97	   7.23	   1.58	    7.68	38.77
Tagelus divisus	     0.21	     1.05	   7.17	   1.01	    7.61	46.39
Abra aequalis	     0.00	     0.87	   6.22	   1.42	    6.60	52.98
Macoma tenta	     0.04	     0.70	   4.91	   0.89	    5.21	58.20
Lucina sp.	     0.00	     0.59	   4.39	   0.73	    4.66	62.85
Parvilucina multilineata	     0.03	     0.62	   4.36	   0.90	    4.63	67.49
Corbula sp.	     0.00	     0.51	   3.81	   1.02	    4.05	71.53
Lyonsia floridana	     0.00	     0.44	   3.20	   0.96	    3.40	74.93
Chione cancellata	     0.00	     0.38	   3.02	   0.42	    3.21	78.14
Caryocorbula sp.	     0.00	     0.40	   2.93	   0.75	    3.11	81.24
Anadara sp.	     0.00	     0.31	   2.47	   0.62	    2.62	83.87
Sphenia sp.	     0.00	     0.13	   0.90	   0.49	    0.96	84.83
Asthenothaerus hemphilli	     0.00	     0.12	   0.87	   0.36	    0.92	85.75
Anadara floridana	     0.00	     0.12	   0.79	   0.33	    0.83	86.59
Divaricella quadrisulcata	     0.00	     0.11	   0.74	   0.45	    0.79	87.38
Lucinisca nassula	     0.00	     0.10	   0.73	   0.44	    0.77	88.15
Bivalvia	     0.00	     0.11	   0.70	   0.44	    0.75	88.89
Timoclea sp.	     0.00	     0.09	   0.66	   0.38	    0.70	89.60
Crassinella lunulata	     0.00	     0.09	   0.62	   0.38	    0.66	90.25

Groups M06  &  M09
Average dissimilarity = 79.73

	Group M06	Group M09	       	       	        	     
Species	 Av.Abund	 Av.Abund	Av.Diss	Diss/SD	Contrib%	Cum.%
Chione sp.	     0.18	     1.71	   9.44	   1.66	   11.84	11.84
Mulinia lateralis	     1.51	     0.32	   7.09	   0.97	    8.90	20.74
Tagelus divisus	     0.83	     1.05	   6.37	   1.06	    7.99	28.73
Nucula proxima	     0.08	     0.97	   5.79	   1.51	    7.26	36.00
Tellina sp	     0.34	     1.06	   5.75	   1.15	    7.21	43.20
Abra aequalis	     0.34	     0.87	   4.24	   1.30	    5.32	48.52
Macoma tenta	     0.29	     0.70	   4.23	   0.99	    5.31	53.83
Lucina sp.	     0.13	     0.59	   3.74	   0.79	    4.69	58.52
Parvilucina multilineata	     0.16	     0.62	   3.63	   0.96	    4.56	63.08
Corbula sp.	     0.17	     0.51	   3.27	   1.06	    4.10	67.18
Lyonsia floridana	     0.11	     0.44	   2.78	   0.97	    3.49	70.67
Caryocorbula sp.	     0.14	     0.40	   2.59	   0.84	    3.25	73.92
Chione cancellata	     0.00	     0.38	   2.45	   0.41	    3.07	76.99
Anadara sp.	     0.05	     0.31	   2.09	   0.65	    2.62	79.61
Amygdalum papyrium	     0.19	     0.04	   1.15	   0.50	    1.44	81.06
Sphenia sp.	     0.07	     0.13	   0.95	   0.59	    1.19	82.25
Bivalvia	     0.05	     0.11	   0.84	   0.51	    1.05	83.30
Asthenothaerus hemphilli	     0.02	     0.12	   0.81	   0.40	    1.01	84.32
Anadara floridana	     0.00	     0.12	   0.66	   0.33	    0.82	85.14
Divaricella quadrisulcata	     0.00	     0.11	   0.62	   0.45	    0.77	85.91
Lucinisca nassula	     0.00	     0.10	   0.60	   0.44	    0.75	86.66
Crassinella lunulata	     0.02	     0.09	   0.57	   0.42	    0.72	87.38
Timoclea sp.	     0.00	     0.09	   0.54	   0.38	    0.68	88.06
Tagelus sp.	     0.07	     0.02	   0.54	   0.23	    0.68	88.73
Lucinoma filosa	     0.10	     0.00	   0.50	   0.31	    0.63	89.37
Tellina versicolor	     0.00	     0.08	   0.48	   0.33	    0.60	89.97
Mysella sp.	     0.00	     0.07	   0.47	   0.32	    0.59	90.56

Groups M07  &  M09
Average dissimilarity = 66.56

	Group M07	Group M09	       	       	        	     
Species	 Av.Abund	 Av.Abund	Av.Diss	Diss/SD	Contrib%	Cum.%
Tellina sp	     2.55	     1.06	   6.98	   1.47	   10.48	10.48
Parvilucina multilineata	     1.77	     0.62	   5.35	   1.21	    8.04	18.53
Macoma tenta	     1.55	     0.70	   5.05	   0.92	    7.59	26.11
Chione sp.	     0.85	     1.71	   4.54	   1.28	    6.82	32.93
Tagelus divisus	     0.82	     1.05	   3.75	   1.04	    5.63	38.56
Abra aequalis	     1.30	     0.87	   2.81	   1.38	    4.22	42.79
Nucula proxima	     0.87	     0.97	   2.70	   1.30	    4.06	46.84
Lucina sp.	     0.45	     0.59	   2.61	   0.94	    3.92	50.76
Mulinia lateralis	     0.68	     0.32	   2.24	   0.98	    3.36	54.13
Lyonsia floridana	     0.65	     0.44	   2.07	   1.26	    3.11	57.24
Corbula sp.	     0.23	     0.51	   1.78	   1.04	    2.68	59.91
Anadara sp.	     0.40	     0.31	   1.76	   0.95	    2.65	62.56
Divaricella quadrisulcata	     0.46	     0.11	   1.73	   0.62	    2.60	65.17
Caryocorbula sp.	     0.32	     0.40	   1.73	   0.93	    2.59	67.76
Chione cancellata	     0.08	     0.38	   1.60	   0.49	    2.41	70.17
Bivalvia	     0.28	     0.11	   1.12	   0.56	    1.68	71.85
Amygdalum papyrium	     0.33	     0.04	   1.08	   0.59	    1.62	73.47
Tellina versicolor	     0.21	     0.08	   1.08	   0.38	    1.62	75.09
Asthenothaerus hemphilli	     0.21	     0.12	   1.06	   0.61	    1.59	76.67
Crassinella lunulata	     0.28	     0.09	   1.03	   0.69	    1.55	78.22
Timoclea sp.	     0.25	     0.09	   0.97	   0.59	    1.45	79.68
Sphenia sp.	     0.20	     0.13	   0.96	   0.68	    1.45	81.13
Crassinella sp.	     0.21	     0.04	   0.73	   0.53	    1.10	82.23
Anadara floridana	     0.08	     0.12	   0.59	   0.44	    0.88	83.11
Lucinisca nassula	     0.11	     0.10	   0.59	   0.58	    0.88	83.99
Lucinidae	     0.16	     0.00	   0.57	   0.29	    0.86	84.85
Mactridae	     0.16	     0.02	   0.54	   0.48	    0.81	85.66
Parvilucina sp.	     0.16	     0.00	   0.49	   0.19	    0.73	86.39
Timoclea grus	     0.11	     0.02	   0.41	   0.38	    0.62	87.01
Divaricella dentata	     0.09	     0.00	   0.40	   0.19	    0.60	87.61
Corbula contracta	     0.05	     0.07	   0.39	   0.35	    0.59	88.20
Mysella sp.	     0.03	     0.07	   0.36	   0.37	    0.55	88.74
Sphenia antillensis	     0.09	     0.00	   0.33	   0.34	    0.49	89.24
Veneridae	     0.10	     0.00	   0.33	   0.40	    0.49	89.73
Lucina nassula	     0.09	     0.00	   0.32	   0.34	    0.48	90.21

Groups M08  &  M09
Average dissimilarity = 62.75

	Group M08	Group M09	       	       	        	     
Species	 Av.Abund	 Av.Abund	Av.Diss	Diss/SD	Contrib%	Cum.%
Tagelus divisus	     1.96	     1.05	   5.56	   1.25	    8.87	 8.87
Abra aequalis	     2.05	     0.87	   4.87	   1.39	    7.76	16.62
Chione sp.	     0.96	     1.71	   4.26	   1.26	    6.79	23.42
Tellina sp	     1.03	     1.06	   4.09	   1.23	    6.52	29.93
Macoma tenta	     0.97	     0.70	   3.55	   1.17	    5.66	35.60
Mulinia lateralis	     1.09	     0.32	   3.29	   1.38	    5.24	40.83
Parvilucina multilineata	     1.08	     0.62	   3.27	   1.35	    5.21	46.04
Nucula proxima	     1.08	     0.97	   2.95	   1.18	    4.71	50.74
Caryocorbula sp.	     0.79	     0.40	   2.93	   1.06	    4.67	55.42
Lucina sp.	     0.34	     0.59	   2.64	   0.89	    4.20	59.62
Corbula sp.	     0.69	     0.51	   2.41	   1.25	    3.84	63.46
Lyonsia floridana	     0.48	     0.44	   1.96	   1.13	    3.13	66.59
Chione cancellata	     0.02	     0.38	   1.52	   0.43	    2.42	69.02
Lucinoma filosa	     0.39	     0.00	   1.46	   0.66	    2.32	71.34
Anadara sp.	     0.17	     0.31	   1.44	   0.74	    2.29	73.63
Asthenothaerus hemphilli	     0.25	     0.12	   1.15	   0.61	    1.83	75.47
Bivalvia	     0.18	     0.11	   0.93	   0.64	    1.48	76.94
Corbula contracta	     0.22	     0.07	   0.85	   0.42	    1.36	78.30
Anadara floridana	     0.12	     0.12	   0.81	   0.53	    1.29	79.59
Amygdalum papyrium	     0.21	     0.04	   0.76	   0.60	    1.21	80.80
Nuculana acuta	     0.20	     0.02	   0.70	   0.49	    1.12	81.92
Divaricella quadrisulcata	     0.11	     0.11	   0.66	   0.56	    1.06	82.98
Sphenia sp.	     0.09	     0.13	   0.63	   0.61	    1.01	83.98
Tellinidae	     0.16	     0.03	   0.61	   0.45	    0.98	84.96
Tellina versicolor	     0.06	     0.08	   0.49	   0.42	    0.78	85.74
Timoclea sp.	     0.06	     0.09	   0.48	   0.48	    0.76	86.50
Crassinella lunulata	     0.06	     0.09	   0.45	   0.47	    0.71	87.21
Corbula chittyana	     0.12	     0.00	   0.44	   0.20	    0.70	87.91
Macoma sp.	     0.13	     0.00	   0.43	   0.29	    0.68	88.60
Lucinisca nassula	     0.02	     0.10	   0.40	   0.48	    0.64	89.24
Crassinella sp.	     0.08	     0.04	   0.40	   0.45	    0.64	89.88
Mactridae	     0.09	     0.02	   0.38	   0.46	    0.60	90.48

Groups M02  &  M10
Average dissimilarity = 93.19

	Group M02	Group M10	       	       	        	     
Species	 Av.Abund	 Av.Abund	Av.Diss	Diss/SD	Contrib%	Cum.%
Mytilopsis leucophaeata	     4.21	     0.00	  14.49	   0.86	   15.55	15.55
Mulinia lateralis	     4.29	     0.79	  13.34	   0.85	   14.32	29.87
Rangia cuneata	     2.40	     0.00	  10.86	   1.06	   11.65	41.52
Chione sp.	     0.00	     1.52	   7.59	   1.26	    8.15	49.67
Mysella sp.	     0.02	     1.26	   6.35	   0.76	    6.81	56.48
Macoma tenta	     0.38	     1.04	   5.16	   0.90	    5.53	62.02
Tellina sp	     1.17	     0.43	   4.76	   1.06	    5.11	67.12
Caryocorbula sp.	     0.00	     0.86	   3.65	   0.81	    3.92	71.05
Tagelus divisus	     0.06	     0.82	   3.55	   0.77	    3.81	74.85
Nucula proxima	     0.00	     0.71	   3.22	   0.83	    3.45	78.30
Amygdalum papyrium	     0.28	     0.38	   2.40	   0.51	    2.58	80.88
Mysella planulata	     0.03	     0.44	   1.89	   0.43	    2.02	82.91
Abra aequalis	     0.00	     0.35	   1.61	   0.75	    1.73	84.64
Corbula sp.	     0.00	     0.30	   1.46	   0.57	    1.57	86.21
Mactridae	     0.28	     0.06	   1.36	   0.43	    1.46	87.67
Lyonsia floridana	     0.23	     0.10	   1.16	   0.56	    1.25	88.91
Bivalvia	     0.02	     0.18	   1.13	   0.30	    1.21	90.12

Groups M03  &  M10
Average dissimilarity = 89.32

	Group M03	Group M10	       	       	        	     
Species	 Av.Abund	 Av.Abund	Av.Diss	Diss/SD	Contrib%	Cum.%
Mulinia lateralis	     2.92	     0.79	  17.59	   1.46	   19.69	19.69
Chione sp.	     0.00	     1.52	  11.71	   1.45	   13.11	32.80
Mysella sp.	     0.00	     1.26	   9.80	   0.85	   10.97	43.78
Macoma tenta	     0.05	     1.04	   7.94	   0.93	    8.89	52.66
Mytilopsis leucophaeata	     0.95	     0.00	   5.53	   0.49	    6.19	58.85
Caryocorbula sp.	     0.00	     0.86	   5.31	   0.88	    5.94	64.80
Tagelus divisus	     0.00	     0.82	   5.19	   0.83	    5.81	70.60
Nucula proxima	     0.00	     0.71	   4.80	   0.93	    5.38	75.98
Tellina sp	     0.10	     0.43	   3.10	   0.84	    3.47	79.46
Amygdalum papyrium	     0.00	     0.38	   2.78	   0.42	    3.11	82.57
Mysella planulata	     0.00	     0.44	   2.55	   0.43	    2.85	85.42
Corbula sp.	     0.05	     0.30	   2.45	   0.65	    2.75	88.17
Abra aequalis	     0.00	     0.35	   2.42	   0.83	    2.71	90.87

Groups M04  &  M10
Average dissimilarity = 88.22

	Group M04	Group M10	       	       	        	     
Species	 Av.Abund	 Av.Abund	Av.Diss	Diss/SD	Contrib%	Cum.%
Mulinia lateralis	     4.11	     0.79	  21.41	   1.21	   24.27	24.27
Chione sp.	     0.00	     1.52	  11.65	   1.37	   13.20	37.47
Mysella sp.	     0.00	     1.26	   9.75	   0.83	   11.05	48.52
Macoma tenta	     0.15	     1.04	   7.73	   0.89	    8.76	57.28
Tagelus divisus	     0.18	     0.82	   5.34	   0.90	    6.05	63.34
Caryocorbula sp.	     0.00	     0.86	   5.25	   0.87	    5.96	69.29
Nucula proxima	     0.00	     0.71	   4.77	   0.91	    5.40	74.69
Tellina sp	     0.11	     0.43	   3.08	   0.82	    3.49	78.19
Amygdalum papyrium	     0.00	     0.38	   2.76	   0.42	    3.13	81.31
Mysella planulata	     0.00	     0.44	   2.52	   0.43	    2.86	84.17
Abra aequalis	     0.00	     0.35	   2.40	   0.81	    2.72	86.89
Corbula sp.	     0.00	     0.30	   2.19	   0.61	    2.48	89.37
Bivalvia	     0.00	     0.18	   1.47	   0.26	    1.67	91.04

Groups M05  &  M10
Average dissimilarity = 89.57

	Group M05	Group M10	       	       	        	     
Species	 Av.Abund	 Av.Abund	Av.Diss	Diss/SD	Contrib%	Cum.%
Chione sp.	     0.00	     1.52	  13.88	   1.43	   15.49	15.49
Mysella sp.	     0.00	     1.26	  11.62	   0.86	   12.97	28.47
Mulinia lateralis	     1.50	     0.79	  11.27	   1.12	   12.58	41.05
Macoma tenta	     0.04	     1.04	   9.47	   0.92	   10.57	51.62
Tagelus divisus	     0.21	     0.82	   6.27	   0.95	    7.00	58.62
Caryocorbula sp.	     0.00	     0.86	   6.06	   0.89	    6.77	65.39
Nucula proxima	     0.00	     0.71	   5.59	   0.95	    6.24	71.63
Tellina sp	     0.00	     0.43	   3.30	   0.76	    3.68	75.31
Amygdalum papyrium	     0.00	     0.38	   3.26	   0.43	    3.63	78.94
Mysella planulata	     0.00	     0.44	   2.90	   0.43	    3.23	82.17
Abra aequalis	     0.00	     0.35	   2.81	   0.84	    3.14	85.32
Corbula sp.	     0.00	     0.30	   2.58	   0.63	    2.88	88.19
Bivalvia	     0.00	     0.18	   1.75	   0.27	    1.96	90.15

Groups M06  &  M10
Average dissimilarity = 79.36

	Group M06	Group M10	       	       	        	     
Species	 Av.Abund	 Av.Abund	Av.Diss	Diss/SD	Contrib%	Cum.%
Chione sp.	     0.18	     1.52	   9.84	   1.43	   12.40	12.40
Mysella sp.	     0.00	     1.26	   9.04	   0.85	   11.40	23.80
Mulinia lateralis	     1.51	     0.79	   8.36	   1.02	   10.54	34.33
Macoma tenta	     0.29	     1.04	   7.02	   0.95	    8.84	43.18
Tagelus divisus	     0.83	     0.82	   6.44	   1.05	    8.11	51.29
Caryocorbula sp.	     0.14	     0.86	   5.13	   0.97	    6.47	57.76
Nucula proxima	     0.08	     0.71	   4.53	   0.95	    5.71	63.47
Tellina sp	     0.34	     0.43	   3.58	   0.97	    4.51	67.98
Amygdalum papyrium	     0.19	     0.38	   3.31	   0.55	    4.18	72.16
Abra aequalis	     0.34	     0.35	   2.91	   0.99	    3.66	75.82
Corbula sp.	     0.17	     0.30	   2.59	   0.74	    3.27	79.09
Mysella planulata	     0.00	     0.44	   2.40	   0.43	    3.02	82.11
Bivalvia	     0.05	     0.18	   1.67	   0.33	    2.10	84.21
Lyonsia floridana	     0.11	     0.10	   1.18	   0.47	    1.49	85.70
Lucina sp.	     0.13	     0.05	   1.13	   0.49	    1.43	87.12
Parvilucina multilineata	     0.16	     0.00	   1.11	   0.57	    1.39	88.52
Sphenia sp.	     0.07	     0.14	   0.90	   0.49	    1.13	89.65
Tagelus sp.	     0.07	     0.05	   0.87	   0.28	    1.09	90.74

Groups M07  &  M10
Average dissimilarity = 76.79

	Group M07	Group M10	       	       	        	     
Species	 Av.Abund	 Av.Abund	Av.Diss	Diss/SD	Contrib%	Cum.%
Tellina sp	     2.55	     0.43	   8.27	   1.39	   10.77	10.77
Parvilucina multilineata	     1.77	     0.00	   6.68	   1.20	    8.70	19.47
Macoma tenta	     1.55	     1.04	   5.65	   0.98	    7.36	26.83
Mysella sp.	     0.03	     1.26	   4.91	   0.84	    6.39	33.22
Chione sp.	     0.85	     1.52	   4.35	   1.25	    5.67	38.90
Tagelus divisus	     0.82	     0.82	   3.74	   0.97	    4.87	43.77
Abra aequalis	     1.30	     0.35	   3.70	   1.58	    4.81	48.58
Mulinia lateralis	     0.68	     0.79	   3.24	   1.08	    4.22	52.80
Nucula proxima	     0.87	     0.71	   3.19	   1.24	    4.16	56.96
Caryocorbula sp.	     0.32	     0.86	   3.09	   1.00	    4.03	60.98
Lyonsia floridana	     0.65	     0.10	   2.24	   1.18	    2.92	63.91
Amygdalum papyrium	     0.33	     0.38	   2.15	   0.63	    2.80	66.71
Lucina sp.	     0.45	     0.05	   1.78	   0.69	    2.32	69.03
Divaricella quadrisulcata	     0.46	     0.00	   1.70	   0.53	    2.22	71.25
Corbula sp.	     0.23	     0.30	   1.51	   0.83	    1.96	73.21
Bivalvia	     0.28	     0.18	   1.50	   0.48	    1.96	75.17
Anadara sp.	     0.40	     0.03	   1.50	   0.77	    1.95	77.12
Mysella planulata	     0.02	     0.44	   1.50	   0.44	    1.95	79.07
Tellina versicolor	     0.21	     0.07	   1.14	   0.35	    1.48	80.55
Asthenothaerus hemphilli	     0.21	     0.11	   1.10	   0.60	    1.44	81.99
Sphenia sp.	     0.20	     0.14	   1.05	   0.61	    1.37	83.35
Crassinella lunulata	     0.28	     0.00	   0.95	   0.58	    1.24	84.59
Timoclea sp.	     0.25	     0.00	   0.81	   0.46	    1.06	85.65
Crassinella sp.	     0.21	     0.00	   0.67	   0.45	    0.87	86.52
Mactridae	     0.16	     0.06	   0.67	   0.54	    0.87	87.38
Lucinidae	     0.16	     0.00	   0.63	   0.28	    0.82	88.20
Parvilucina sp.	     0.16	     0.00	   0.52	   0.19	    0.68	88.88
Chione cancellata	     0.08	     0.05	   0.48	   0.43	    0.62	89.51
Divaricella dentata	     0.09	     0.00	   0.44	   0.19	    0.58	90.09

Groups M08  &  M10
Average dissimilarity = 71.20

	Group M08	Group M10	       	       	        	     
Species	 Av.Abund	 Av.Abund	Av.Diss	Diss/SD	Contrib%	Cum.%
Abra aequalis	     2.05	     0.35	   6.85	   1.69	    9.61	 9.61
Tagelus divisus	     1.96	     0.82	   6.42	   1.27	    9.02	18.63
Mysella sp.	     0.02	     1.26	   5.10	   0.84	    7.17	25.80
Macoma tenta	     0.97	     1.04	   4.28	   1.15	    6.01	31.81
Parvilucina multilineata	     1.08	     0.00	   4.13	   1.52	    5.80	37.61
Chione sp.	     0.96	     1.52	   4.05	   1.22	    5.69	43.30
Caryocorbula sp.	     0.79	     0.86	   3.89	   1.11	    5.47	48.76
Tellina sp	     1.03	     0.43	   3.86	   1.08	    5.43	54.19
Mulinia lateralis	     1.09	     0.79	   3.63	   1.24	    5.10	59.29
Nucula proxima	     1.08	     0.71	   3.63	   1.19	    5.10	64.39
Corbula sp.	     0.69	     0.30	   2.64	   1.13	    3.71	68.10
Amygdalum papyrium	     0.21	     0.38	   1.93	   0.57	    2.72	70.82
Lyonsia floridana	     0.48	     0.10	   1.89	   0.93	    2.65	73.47
Lucina sp.	     0.34	     0.05	   1.68	   0.59	    2.35	75.82
Lucinoma filosa	     0.39	     0.00	   1.61	   0.64	    2.26	78.08
Mysella planulata	     0.02	     0.44	   1.56	   0.44	    2.20	80.28
Bivalvia	     0.18	     0.18	   1.37	   0.48	    1.92	82.20
Asthenothaerus hemphilli	     0.25	     0.11	   1.20	   0.61	    1.68	83.88
Nuculana acuta	     0.20	     0.05	   0.84	   0.54	    1.18	85.06
Corbula contracta	     0.22	     0.00	   0.69	   0.34	    0.97	86.03
Anadara sp.	     0.17	     0.03	   0.67	   0.47	    0.94	86.97
Sphenia sp.	     0.09	     0.14	   0.63	   0.49	    0.88	87.86
Tellinidae	     0.16	     0.00	   0.60	   0.41	    0.84	88.70
Macoma sp.	     0.13	     0.03	   0.55	   0.34	    0.77	89.47
Anadara floridana	     0.12	     0.00	   0.54	   0.41	    0.76	90.23

Groups M09  &  M10
Average dissimilarity = 69.22

	Group M09	Group M10	       	       	        	     
Species	 Av.Abund	 Av.Abund	Av.Diss	Diss/SD	Contrib%	Cum.%
Mysella sp.	     0.07	     1.26	   5.85	   0.88	    8.44	 8.44
Tagelus divisus	     1.05	     0.82	   5.07	   1.11	    7.33	15.77
Chione sp.	     1.71	     1.52	   4.80	   1.20	    6.94	22.71
Macoma tenta	     0.70	     1.04	   4.64	   1.13	    6.70	29.41
Tellina sp	     1.06	     0.43	   4.57	   1.14	    6.61	36.02
Caryocorbula sp.	     0.40	     0.86	   3.81	   1.13	    5.50	41.52
Nucula proxima	     0.97	     0.71	   3.81	   1.32	    5.50	47.02
Mulinia lateralis	     0.32	     0.79	   3.51	   1.02	    5.07	52.09
Abra aequalis	     0.87	     0.35	   3.38	   1.25	    4.89	56.98
Parvilucina multilineata	     0.62	     0.00	   2.81	   0.87	    4.06	61.04
Lucina sp.	     0.59	     0.05	   2.81	   0.76	    4.06	65.10
Corbula sp.	     0.51	     0.30	   2.53	   1.12	    3.65	68.75
Lyonsia floridana	     0.44	     0.10	   2.06	   0.95	    2.98	71.73
Chione cancellata	     0.38	     0.05	   2.01	   0.45	    2.90	74.63
Amygdalum papyrium	     0.04	     0.38	   1.81	   0.46	    2.62	77.24
Mysella planulata	     0.00	     0.44	   1.70	   0.43	    2.46	79.70
Anadara sp.	     0.31	     0.03	   1.55	   0.65	    2.25	81.95
Bivalvia	     0.11	     0.18	   1.24	   0.40	    1.79	83.74
Sphenia sp.	     0.13	     0.14	   0.92	   0.60	    1.33	85.07
Asthenothaerus hemphilli	     0.12	     0.11	   0.91	   0.53	    1.31	86.37
Tellina versicolor	     0.08	     0.07	   0.64	   0.38	    0.92	87.30
Anadara floridana	     0.12	     0.00	   0.52	   0.33	    0.75	88.04
Divaricella quadrisulcata	     0.11	     0.00	   0.48	   0.45	    0.70	88.74
Lucinisca nassula	     0.10	     0.00	   0.46	   0.44	    0.67	89.41
Timoclea sp.	     0.09	     0.00	   0.42	   0.38	    0.60	90.01

Groups M02  &  M11
Average dissimilarity = 93.35

	Group M02	Group M11	       	       	        	     
Species	 Av.Abund	 Av.Abund	Av.Diss	Diss/SD	Contrib%	Cum.%
Mytilopsis leucophaeata	     4.21	     0.02	  15.62	   0.88	   16.73	16.73
Mulinia lateralis	     4.29	     0.54	  13.66	   0.81	   14.64	31.37
Rangia cuneata	     2.40	     0.00	  12.01	   1.04	   12.87	44.24
Chione sp.	     0.00	     1.24	   6.44	   0.94	    6.90	51.14
Tellina sp	     1.17	     0.53	   5.63	   1.03	    6.03	57.17
Nucula proxima	     0.00	     0.95	   5.19	   0.73	    5.56	62.73
Tagelus divisus	     0.06	     0.86	   4.13	   0.63	    4.42	67.15
Macoma tenta	     0.38	     0.69	   4.00	   1.03	    4.28	71.43
Corbula sp.	     0.00	     0.47	   2.49	   0.75	    2.67	74.10
Abra aequalis	     0.00	     0.49	   2.47	   0.70	    2.65	76.75
Caryocorbula sp.	     0.00	     0.39	   2.39	   0.61	    2.56	79.31
Mysella sp.	     0.02	     0.35	   1.75	   0.49	    1.87	81.18
Lyonsia floridana	     0.23	     0.21	   1.63	   0.62	    1.74	82.92
Amygdalum papyrium	     0.28	     0.14	   1.58	   0.43	    1.69	84.61
Mactridae	     0.28	     0.04	   1.47	   0.40	    1.58	86.19
Polymesoda caroliniana	     0.32	     0.00	   1.17	   0.35	    1.25	87.44
Polymesoda sp.	     0.10	     0.00	   1.06	   0.19	    1.14	88.58
Tagelus plebeius	     0.25	     0.00	   1.02	   0.51	    1.09	89.67
Mysella planulata	     0.03	     0.14	   0.79	   0.40	    0.85	90.52

Groups M03  &  M11
Average dissimilarity = 90.57

	Group M03	Group M11	       	       	        	     
Species	 Av.Abund	 Av.Abund	Av.Diss	Diss/SD	Contrib%	Cum.%
Mulinia lateralis	     2.92	     0.54	  21.59	   1.38	   23.84	23.84
Chione sp.	     0.00	     1.24	  10.06	   1.08	   11.11	34.95
Nucula proxima	     0.00	     0.95	   8.19	   0.81	    9.04	43.99
Mytilopsis leucophaeata	     0.95	     0.02	   6.34	   0.50	    7.00	50.98
Tagelus divisus	     0.00	     0.86	   6.12	   0.66	    6.76	57.74
Macoma tenta	     0.05	     0.69	   5.38	   1.07	    5.94	63.68
Tellina sp	     0.10	     0.53	   5.13	   0.76	    5.67	69.35
Corbula sp.	     0.05	     0.47	   4.12	   0.84	    4.55	73.90
Caryocorbula sp.	     0.00	     0.39	   4.04	   0.64	    4.46	78.36
Abra aequalis	     0.00	     0.49	   3.79	   0.78	    4.18	82.54
Mysella sp.	     0.00	     0.35	   2.62	   0.53	    2.89	85.43
Lyonsia floridana	     0.00	     0.21	   1.50	   0.47	    1.66	87.09
Amygdalum papyrium	     0.00	     0.14	   1.29	   0.28	    1.43	88.52
Mysella planulata	     0.00	     0.14	   0.97	   0.38	    1.07	89.59
Chione cancellata	     0.00	     0.11	   0.95	   0.25	    1.05	90.64

Groups M04  &  M11
Average dissimilarity = 89.57

	Group M04	Group M11	       	       	        	     
Species	 Av.Abund	 Av.Abund	Av.Diss	Diss/SD	Contrib%	Cum.%
Mulinia lateralis	     4.11	     0.54	  25.13	   1.23	   28.05	28.05
Chione sp.	     0.00	     1.24	  10.01	   1.05	   11.18	39.23
Nucula proxima	     0.00	     0.95	   8.15	   0.78	    9.10	48.33
Tagelus divisus	     0.18	     0.86	   6.61	   0.74	    7.38	55.71
Macoma tenta	     0.15	     0.69	   5.31	   1.05	    5.93	61.64
Tellina sp	     0.11	     0.53	   5.15	   0.74	    5.75	67.39
Caryocorbula sp.	     0.00	     0.39	   4.06	   0.62	    4.53	71.92
Corbula sp.	     0.00	     0.47	   3.91	   0.81	    4.37	76.29
Abra aequalis	     0.00	     0.49	   3.76	   0.76	    4.20	80.49
Mysella sp.	     0.00	     0.35	   2.60	   0.52	    2.90	83.39
Lyonsia floridana	     0.00	     0.21	   1.49	   0.47	    1.66	85.06
Amygdalum papyrium	     0.00	     0.14	   1.29	   0.27	    1.44	86.50
Mysella planulata	     0.00	     0.14	   0.96	   0.37	    1.07	87.57
Chione cancellata	     0.00	     0.11	   0.94	   0.25	    1.06	88.63
Sphenia sp.	     0.00	     0.11	   0.85	   0.39	    0.95	89.58
Tellina versicolor	     0.00	     0.07	   0.84	   0.25	    0.93	90.51

Groups M05  &  M11
Average dissimilarity = 90.46

	Group M05	Group M11	       	       	        	     
Species	 Av.Abund	 Av.Abund	Av.Diss	Diss/SD	Contrib%	Cum.%
Mulinia lateralis	     1.50	     0.54	  13.35	   1.01	   14.76	14.76
Chione sp.	     0.00	     1.24	  12.03	   1.10	   13.30	28.06
Nucula proxima	     0.00	     0.95	   9.82	   0.81	   10.85	38.91
Tagelus divisus	     0.21	     0.86	   7.96	   0.78	    8.80	47.71
Macoma tenta	     0.04	     0.69	   6.40	   1.09	    7.08	54.79
Tellina sp	     0.00	     0.53	   6.14	   0.71	    6.79	61.58
Caryocorbula sp.	     0.00	     0.39	   5.12	   0.63	    5.66	67.24
Corbula sp.	     0.00	     0.47	   4.73	   0.84	    5.23	72.47
Abra aequalis	     0.00	     0.49	   4.45	   0.79	    4.92	77.40
Mysella sp.	     0.00	     0.35	   3.06	   0.54	    3.39	80.78
Lyonsia floridana	     0.00	     0.21	   1.75	   0.48	    1.93	82.71
Amygdalum papyrium	     0.00	     0.14	   1.56	   0.28	    1.72	84.43
Chione cancellata	     0.00	     0.11	   1.13	   0.26	    1.24	85.68
Mysella planulata	     0.00	     0.14	   1.12	   0.38	    1.24	86.92
Bivalvia	     0.00	     0.06	   1.08	   0.30	    1.19	88.11
Tellina versicolor	     0.00	     0.07	   1.07	   0.26	    1.18	89.30
Sphenia sp.	     0.00	     0.11	   1.01	   0.40	    1.12	90.41

Groups M06  &  M11
Average dissimilarity = 79.60

	Group M06	Group M11	       	       	        	     
Species	 Av.Abund	 Av.Abund	Av.Diss	Diss/SD	Contrib%	Cum.%
Mulinia lateralis	     1.51	     0.54	   9.27	   0.98	   11.65	11.65
Chione sp.	     0.18	     1.24	   8.78	   1.11	   11.02	22.67
Tagelus divisus	     0.83	     0.86	   8.08	   0.97	   10.15	32.83
Nucula proxima	     0.08	     0.95	   7.50	   0.82	    9.42	42.24
Tellina sp	     0.34	     0.53	   5.21	   0.90	    6.55	48.79
Macoma tenta	     0.29	     0.69	   5.18	   1.10	    6.51	55.30
Abra aequalis	     0.34	     0.49	   4.39	   0.98	    5.51	60.82
Corbula sp.	     0.17	     0.47	   3.96	   0.92	    4.97	65.79
Caryocorbula sp.	     0.14	     0.39	   3.81	   0.75	    4.78	70.57
Mysella sp.	     0.00	     0.35	   2.43	   0.52	    3.06	73.63
Amygdalum papyrium	     0.19	     0.14	   2.29	   0.47	    2.87	76.50
Lyonsia floridana	     0.11	     0.21	   1.89	   0.55	    2.37	78.87
Lucina sp.	     0.13	     0.09	   1.37	   0.52	    1.73	80.60
Parvilucina multilineata	     0.16	     0.00	   1.28	   0.55	    1.60	82.20
Sphenia sp.	     0.07	     0.11	   1.08	   0.51	    1.36	83.56
Bivalvia	     0.05	     0.06	   1.06	   0.40	    1.33	84.89
Mysella planulata	     0.00	     0.14	   0.90	   0.38	    1.13	86.03
Chione cancellata	     0.00	     0.11	   0.88	   0.25	    1.10	87.13
Nuculana acuta	     0.00	     0.12	   0.76	   0.33	    0.95	88.08
Tellina versicolor	     0.00	     0.07	   0.73	   0.26	    0.92	89.00
Lucinoma filosa	     0.10	     0.00	   0.70	   0.27	    0.88	89.87
Mytilopsis leucophaeata	     0.06	     0.02	   0.60	   0.25	    0.75	90.62

Groups M07  &  M11
Average dissimilarity = 76.37

	Group M07	Group M11	       	       	        	     
Species	 Av.Abund	 Av.Abund	Av.Diss	Diss/SD	Contrib%	Cum.%
Tellina sp	     2.55	     0.53	   8.71	   1.43	   11.40	11.40
Parvilucina multilineata	     1.77	     0.00	   7.19	   1.19	    9.42	20.82
Macoma tenta	     1.55	     0.69	   5.67	   0.90	    7.42	28.24
Chione sp.	     0.85	     1.24	   4.57	   1.10	    5.98	34.22
Tagelus divisus	     0.82	     0.86	   4.45	   0.91	    5.83	40.05
Abra aequalis	     1.30	     0.49	   4.03	   1.60	    5.28	45.33
Nucula proxima	     0.87	     0.95	   3.99	   1.01	    5.22	50.55
Mulinia lateralis	     0.68	     0.54	   2.90	   1.04	    3.80	54.35
Lyonsia floridana	     0.65	     0.21	   2.47	   1.17	    3.23	57.58
Corbula sp.	     0.23	     0.47	   2.03	   0.96	    2.65	60.23
Caryocorbula sp.	     0.32	     0.39	   2.01	   0.91	    2.63	62.87
Lucina sp.	     0.45	     0.09	   1.95	   0.70	    2.56	65.42
Divaricella quadrisulcata	     0.46	     0.03	   1.89	   0.54	    2.48	67.90
Amygdalum papyrium	     0.33	     0.14	   1.60	   0.58	    2.10	70.00
Anadara sp.	     0.40	     0.00	   1.59	   0.75	    2.08	72.08
Mysella sp.	     0.03	     0.35	   1.42	   0.53	    1.87	73.95
Tellina versicolor	     0.21	     0.07	   1.28	   0.37	    1.68	75.63
Bivalvia	     0.28	     0.06	   1.20	   0.52	    1.56	77.19
Sphenia sp.	     0.20	     0.11	   1.10	   0.62	    1.44	78.63
Crassinella lunulata	     0.28	     0.00	   1.02	   0.58	    1.33	79.96
Asthenothaerus hemphilli	     0.21	     0.02	   0.99	   0.51	    1.30	81.27
Timoclea sp.	     0.25	     0.00	   0.86	   0.46	    1.13	82.39
Chione cancellata	     0.08	     0.11	   0.76	   0.40	    1.00	83.39
Crassinella sp.	     0.21	     0.00	   0.71	   0.45	    0.93	84.32
Lucinidae	     0.16	     0.00	   0.68	   0.28	    0.89	85.22
Mactridae	     0.16	     0.04	   0.66	   0.52	    0.87	86.08
Mysella planulata	     0.02	     0.14	   0.56	   0.40	    0.74	86.82
Parvilucina sp.	     0.16	     0.00	   0.56	   0.19	    0.73	87.55
Nuculana acuta	     0.02	     0.12	   0.49	   0.36	    0.64	88.19
Divaricella dentata	     0.09	     0.00	   0.49	   0.19	    0.64	88.83
Corbula contracta	     0.05	     0.08	   0.48	   0.35	    0.62	89.45
Veneridae	     0.10	     0.02	   0.47	   0.43	    0.61	90.07

Groups M08  &  M11
Average dissimilarity = 70.75

	Group M08	Group M11	       	       	        	     
Species	 Av.Abund	 Av.Abund	Av.Diss	Diss/SD	Contrib%	Cum.%
Tagelus divisus	     1.96	     0.86	   7.34	   1.28	   10.38	10.38
Abra aequalis	     2.05	     0.49	   7.06	   1.52	    9.98	20.36
Chione sp.	     0.96	     1.24	   4.48	   1.13	    6.33	26.70
Parvilucina multilineata	     1.08	     0.00	   4.44	   1.51	    6.27	32.97
Nucula proxima	     1.08	     0.95	   4.38	   1.00	    6.19	39.15
Tellina sp	     1.03	     0.53	   4.35	   1.10	    6.15	45.30
Macoma tenta	     0.97	     0.69	   3.92	   1.20	    5.54	50.85
Mulinia lateralis	     1.09	     0.54	   3.76	   1.29	    5.31	56.16
Caryocorbula sp.	     0.79	     0.39	   3.41	   1.05	    4.82	60.97
Corbula sp.	     0.69	     0.47	   2.91	   1.18	    4.11	65.08
Lyonsia floridana	     0.48	     0.21	   2.18	   0.96	    3.09	68.16
Lucina sp.	     0.34	     0.09	   1.87	   0.60	    2.64	70.81
Lucinoma filosa	     0.39	     0.00	   1.76	   0.62	    2.48	73.29
Mysella sp.	     0.02	     0.35	   1.45	   0.52	    2.05	75.34
Amygdalum papyrium	     0.21	     0.14	   1.29	   0.51	    1.82	77.16
Nuculana acuta	     0.20	     0.12	   1.11	   0.57	    1.56	78.72
Asthenothaerus hemphilli	     0.25	     0.02	   1.08	   0.51	    1.53	80.25
Bivalvia	     0.18	     0.06	   1.01	   0.58	    1.43	81.68
Corbula contracta	     0.22	     0.08	   0.99	   0.42	    1.40	83.08
Sphenia sp.	     0.09	     0.11	   0.67	   0.53	    0.94	84.03
Tellinidae	     0.16	     0.00	   0.64	   0.41	    0.91	84.94
Anadara sp.	     0.17	     0.00	   0.63	   0.43	    0.89	85.83
Mysella planulata	     0.02	     0.14	   0.61	   0.41	    0.86	86.69
Anadara floridana	     0.12	     0.00	   0.59	   0.41	    0.84	87.53
Chione cancellata	     0.02	     0.11	   0.58	   0.30	    0.81	88.34
Tellina versicolor	     0.06	     0.07	   0.57	   0.38	    0.81	89.15
Divaricella quadrisulcata	     0.11	     0.03	   0.53	   0.39	    0.74	89.89
Corbula chittyana	     0.12	     0.00	   0.51	   0.20	    0.73	90.62

Groups M09  &  M11
Average dissimilarity = 68.58

	Group M09	Group M11	       	       	        	     
Species	 Av.Abund	 Av.Abund	Av.Diss	Diss/SD	Contrib%	Cum.%
Chione sp.	     1.71	     1.24	   6.57	   1.28	    9.58	 9.58
Tagelus divisus	     1.05	     0.86	   6.01	   1.05	    8.77	18.34
Tellina sp	     1.06	     0.53	   5.14	   1.19	    7.49	25.83
Nucula proxima	     0.97	     0.95	   4.69	   1.08	    6.84	32.68
Macoma tenta	     0.70	     0.69	   4.00	   1.17	    5.84	38.52
Abra aequalis	     0.87	     0.49	   3.91	   1.26	    5.70	44.21
Lucina sp.	     0.59	     0.09	   3.09	   0.77	    4.50	48.71
Parvilucina multilineata	     0.62	     0.00	   3.06	   0.86	    4.46	53.18
Corbula sp.	     0.51	     0.47	   2.86	   1.17	    4.18	57.35
Mulinia lateralis	     0.32	     0.54	   2.82	   1.02	    4.12	61.47
Caryocorbula sp.	     0.40	     0.39	   2.69	   1.00	    3.93	65.40
Lyonsia floridana	     0.44	     0.21	   2.44	   1.02	    3.56	68.96
Chione cancellata	     0.38	     0.11	   2.43	   0.48	    3.54	72.50
Mysella sp.	     0.07	     0.35	   1.83	   0.59	    2.66	75.17
Anadara sp.	     0.31	     0.00	   1.67	   0.62	    2.43	77.60
Sphenia sp.	     0.13	     0.11	   0.98	   0.63	    1.44	79.03
Amygdalum papyrium	     0.04	     0.14	   0.89	   0.33	    1.30	80.33
Bivalvia	     0.11	     0.06	   0.78	   0.54	    1.14	81.46
Tellina versicolor	     0.08	     0.07	   0.76	   0.43	    1.10	82.57
Asthenothaerus hemphilli	     0.12	     0.02	   0.68	   0.40	    0.99	83.55
Corbula contracta	     0.07	     0.08	   0.66	   0.35	    0.96	84.51
Divaricella quadrisulcata	     0.11	     0.03	   0.63	   0.49	    0.92	85.43
Mysella planulata	     0.00	     0.14	   0.61	   0.38	    0.90	86.33
Nuculana acuta	     0.02	     0.12	   0.59	   0.37	    0.86	87.18
Anadara floridana	     0.12	     0.00	   0.56	   0.33	    0.82	88.00
Lucinisca nassula	     0.10	     0.00	   0.50	   0.44	    0.73	88.73
Laevicardium sp.	     0.05	     0.06	   0.47	   0.32	    0.68	89.41
Timoclea sp.	     0.09	     0.00	   0.46	   0.38	    0.66	90.08

Groups M10  &  M11
Average dissimilarity = 68.88

	Group M10	Group M11	       	       	        	     
Species	 Av.Abund	 Av.Abund	Av.Diss	Diss/SD	Contrib%	Cum.%
Mysella sp.	     1.26	     0.35	   7.45	   0.91	   10.81	10.81
Chione sp.	     1.52	     1.24	   6.87	   1.18	    9.97	20.79
Tagelus divisus	     0.82	     0.86	   5.95	   0.96	    8.64	29.43
Nucula proxima	     0.71	     0.95	   5.51	   0.95	    8.00	37.43
Macoma tenta	     1.04	     0.69	   5.48	   1.01	    7.96	45.39
Caryocorbula sp.	     0.86	     0.39	   4.64	   1.11	    6.74	52.12
Mulinia lateralis	     0.79	     0.54	   4.62	   1.02	    6.71	58.84
Tellina sp	     0.43	     0.53	   3.87	   0.93	    5.62	64.45
Abra aequalis	     0.35	     0.49	   3.06	   1.03	    4.44	68.90
Corbula sp.	     0.30	     0.47	   3.02	   1.00	    4.38	73.28
Amygdalum papyrium	     0.38	     0.14	   2.71	   0.50	    3.94	77.22
Mysella planulata	     0.44	     0.14	   2.51	   0.53	    3.65	80.87
Bivalvia	     0.18	     0.06	   1.44	   0.35	    2.09	82.96
Lyonsia floridana	     0.10	     0.21	   1.43	   0.60	    2.07	85.03
Sphenia sp.	     0.14	     0.11	   1.02	   0.54	    1.48	86.50
Chione cancellata	     0.05	     0.11	   0.87	   0.32	    1.27	87.77
Tellina versicolor	     0.07	     0.07	   0.80	   0.35	    1.15	88.93
Nuculana acuta	     0.05	     0.12	   0.79	   0.43	    1.15	90.07

Groups M02  &  M12
Average dissimilarity = 93.44

	Group M02	Group M12	       	       	        	     
Species	 Av.Abund	 Av.Abund	Av.Diss	Diss/SD	Contrib%	Cum.%
Mytilopsis leucophaeata	     4.21	     0.02	  11.47	   0.80	   12.28	12.28
Nucula proxima	     0.00	     2.94	  10.40	   1.48	   11.14	23.41
Mulinia lateralis	     4.29	     0.17	   9.97	   0.68	   10.67	34.08
Rangia cuneata	     2.40	     0.00	   8.20	   1.06	    8.77	42.85
Tellina sp	     1.17	     1.71	   6.48	   0.96	    6.93	49.79
Parvilucina multilineata	     0.02	     1.93	   6.36	   0.93	    6.81	56.60
Macoma tenta	     0.38	     1.68	   5.31	   0.99	    5.68	62.28
Abra aequalis	     0.00	     1.38	   4.53	   1.34	    4.85	67.13
Chione sp.	     0.00	     0.95	   3.37	   1.45	    3.61	70.74
Tagelus divisus	     0.06	     0.89	   2.77	   0.62	    2.97	73.71
Lyonsia floridana	     0.23	     0.48	   1.89	   0.87	    2.02	75.73
Tellina versicolor	     0.00	     0.53	   1.67	   0.41	    1.78	77.52
Crassinella lunulata	     0.00	     0.47	   1.60	   0.94	    1.71	79.22
Divaricella quadrisulcata	     0.00	     0.37	   1.35	   0.51	    1.44	80.66
Caryocorbula sp.	     0.00	     0.33	   1.28	   0.72	    1.37	82.04
Lucina sp.	     0.06	     0.26	   1.12	   0.49	    1.20	83.23
Mactridae	     0.28	     0.06	   1.02	   0.45	    1.09	84.32
Anadara sp.	     0.00	     0.33	   0.93	   0.56	    0.99	85.31
Polymesoda caroliniana	     0.32	     0.00	   0.88	   0.35	    0.94	86.25
Amygdalum papyrium	     0.28	     0.07	   0.83	   0.48	    0.89	87.14
Tagelus plebeius	     0.25	     0.02	   0.81	   0.54	    0.86	88.01
Parvilucina sp.	     0.00	     0.19	   0.70	   0.24	    0.75	88.76
Corbula sp.	     0.00	     0.20	   0.66	   0.53	    0.71	89.47
Crassinella sp.	     0.00	     0.16	   0.61	   0.46	    0.65	90.12

Groups M03  &  M12
Average dissimilarity = 97.36

	Group M03	Group M12	       	       	        	     
Species	 Av.Abund	 Av.Abund	Av.Diss	Diss/SD	Contrib%	Cum.%
Nucula proxima	     0.00	     2.94	  14.38	   1.70	   14.77	14.77
Mulinia lateralis	     2.92	     0.17	  13.54	   1.45	   13.90	28.68
Tellina sp	     0.10	     1.71	   9.11	   0.93	    9.36	38.04
Parvilucina multilineata	     0.00	     1.93	   8.63	   1.00	    8.87	46.91
Macoma tenta	     0.05	     1.68	   7.17	   1.02	    7.36	54.27
Abra aequalis	     0.00	     1.38	   6.14	   1.53	    6.31	60.58
Chione sp.	     0.00	     0.95	   4.67	   1.68	    4.79	65.37
Mytilopsis leucophaeata	     0.95	     0.02	   4.00	   0.49	    4.11	69.48
Tagelus divisus	     0.00	     0.89	   3.65	   0.65	    3.75	73.22
Lyonsia floridana	     0.00	     0.48	   2.41	   0.85	    2.48	75.70
Tellina versicolor	     0.00	     0.53	   2.26	   0.43	    2.32	78.02
Crassinella lunulata	     0.00	     0.47	   2.17	   1.00	    2.23	80.26
Divaricella quadrisulcata	     0.00	     0.37	   1.86	   0.53	    1.91	82.17
Caryocorbula sp.	     0.00	     0.33	   1.81	   0.77	    1.86	84.03
Lucina sp.	     0.00	     0.26	   1.38	   0.44	    1.41	85.44
Anadara sp.	     0.00	     0.33	   1.20	   0.59	    1.24	86.68
Corbula sp.	     0.05	     0.20	   1.10	   0.56	    1.13	87.81
Parvilucina sp.	     0.00	     0.19	   0.98	   0.25	    1.01	88.82
Lucinisca nassula	     0.00	     0.14	   0.90	   0.45	    0.92	89.74
Crassinella sp.	     0.00	     0.16	   0.86	   0.48	    0.89	90.63

Groups M04  &  M12
Average dissimilarity = 96.22

	Group M04	Group M12	       	       	        	     
Species	 Av.Abund	 Av.Abund	Av.Diss	Diss/SD	Contrib%	Cum.%
Mulinia lateralis	     4.11	     0.17	  16.57	   1.14	   17.22	17.22
Nucula proxima	     0.00	     2.94	  14.22	   1.65	   14.78	32.00
Tellina sp	     0.11	     1.71	   9.02	   0.91	    9.37	41.38
Parvilucina multilineata	     0.03	     1.93	   8.50	   0.98	    8.83	50.21
Macoma tenta	     0.15	     1.68	   7.00	   1.01	    7.28	57.49
Abra aequalis	     0.00	     1.38	   6.07	   1.50	    6.31	63.80
Chione sp.	     0.00	     0.95	   4.61	   1.63	    4.79	68.59
Tagelus divisus	     0.18	     0.89	   3.81	   0.71	    3.96	72.56
Lyonsia floridana	     0.00	     0.48	   2.38	   0.83	    2.48	75.03
Tellina versicolor	     0.00	     0.53	   2.24	   0.43	    2.32	77.36
Crassinella lunulata	     0.00	     0.47	   2.15	   0.99	    2.23	79.59
Divaricella quadrisulcata	     0.00	     0.37	   1.84	   0.53	    1.91	81.51
Caryocorbula sp.	     0.00	     0.33	   1.79	   0.76	    1.86	83.37
Lucina sp.	     0.00	     0.26	   1.36	   0.44	    1.41	84.78
Anadara sp.	     0.00	     0.33	   1.19	   0.59	    1.24	86.02
Parvilucina sp.	     0.00	     0.19	   0.97	   0.25	    1.01	87.03
Lucinisca nassula	     0.00	     0.14	   0.89	   0.45	    0.93	87.96
Corbula sp.	     0.00	     0.20	   0.88	   0.55	    0.92	88.87
Crassinella sp.	     0.00	     0.16	   0.85	   0.48	    0.89	89.76
Nuculana acuta	     0.00	     0.17	   0.60	   0.29	    0.62	90.38

Groups M05  &  M12
Average dissimilarity = 97.13

	Group M05	Group M12	       	       	        	     
Species	 Av.Abund	 Av.Abund	Av.Diss	Diss/SD	Contrib%	Cum.%
Nucula proxima	     0.00	     2.94	  16.03	   1.73	   16.51	16.51
Tellina sp	     0.00	     1.71	  10.39	   0.91	   10.70	27.21
Parvilucina multilineata	     0.03	     1.93	   9.47	   1.01	    9.75	36.96
Macoma tenta	     0.04	     1.68	   7.90	   1.02	    8.13	45.09
Mulinia lateralis	     1.50	     0.17	   7.67	   1.03	    7.90	52.99
Abra aequalis	     0.00	     1.38	   6.78	   1.56	    6.99	59.98
Chione sp.	     0.00	     0.95	   5.20	   1.72	    5.36	65.33
Tagelus divisus	     0.21	     0.89	   4.29	   0.75	    4.42	69.75
Lyonsia floridana	     0.00	     0.48	   2.70	   0.84	    2.78	72.53
Tellina versicolor	     0.00	     0.53	   2.51	   0.44	    2.58	75.11
Crassinella lunulata	     0.00	     0.47	   2.41	   1.00	    2.48	77.59
Divaricella quadrisulcata	     0.00	     0.37	   2.07	   0.53	    2.13	79.72
Caryocorbula sp.	     0.00	     0.33	   2.05	   0.77	    2.11	81.83
Lucina sp.	     0.00	     0.26	   1.54	   0.44	    1.59	83.41
Anadara sp.	     0.00	     0.33	   1.31	   0.60	    1.34	84.76
Parvilucina sp.	     0.00	     0.19	   1.10	   0.25	    1.13	85.89
Lucinisca nassula	     0.00	     0.14	   1.04	   0.45	    1.07	86.96
Corbula sp.	     0.00	     0.20	   0.98	   0.56	    1.01	87.98
Crassinella sp.	     0.00	     0.16	   0.97	   0.49	    1.00	88.97
Nuculana acuta	     0.00	     0.17	   0.66	   0.30	    0.68	89.66
Bivalvia	     0.00	     0.09	   0.61	   0.34	    0.63	90.28

Groups M06  &  M12
Average dissimilarity = 85.63

	Group M06	Group M12	       	       	        	     
Species	 Av.Abund	 Av.Abund	Av.Diss	Diss/SD	Contrib%	Cum.%
Nucula proxima	     0.08	     2.94	  13.41	   1.65	   15.66	15.66
Tellina sp	     0.34	     1.71	   8.41	   0.98	    9.82	25.48
Parvilucina multilineata	     0.16	     1.93	   7.98	   1.00	    9.32	34.80
Macoma tenta	     0.29	     1.68	   6.69	   1.04	    7.82	42.62
Mulinia lateralis	     1.51	     0.17	   6.19	   0.96	    7.23	49.85
Abra aequalis	     0.34	     1.38	   5.00	   1.40	    5.84	55.69
Tagelus divisus	     0.83	     0.89	   4.88	   0.88	    5.70	61.39
Chione sp.	     0.18	     0.95	   3.75	   1.41	    4.38	65.77
Lyonsia floridana	     0.11	     0.48	   2.40	   0.89	    2.80	68.57
Tellina versicolor	     0.00	     0.53	   2.15	   0.43	    2.52	71.09
Crassinella lunulata	     0.02	     0.47	   2.07	   1.01	    2.41	73.50
Caryocorbula sp.	     0.14	     0.33	   1.85	   0.86	    2.16	75.66
Divaricella quadrisulcata	     0.00	     0.37	   1.77	   0.53	    2.06	77.73
Lucina sp.	     0.13	     0.26	   1.66	   0.57	    1.94	79.66
Corbula sp.	     0.17	     0.20	   1.33	   0.65	    1.56	81.22
Anadara sp.	     0.05	     0.33	   1.28	   0.64	    1.49	82.71
Parvilucina sp.	     0.03	     0.19	   1.02	   0.27	    1.19	83.90
Amygdalum papyrium	     0.19	     0.07	   0.99	   0.52	    1.16	85.06
Crassinella sp.	     0.02	     0.16	   0.87	   0.51	    1.01	86.07
Lucinisca nassula	     0.00	     0.14	   0.84	   0.46	    0.98	87.05
Bivalvia	     0.05	     0.09	   0.69	   0.43	    0.81	87.86
Lucinoma filosa	     0.10	     0.03	   0.59	   0.37	    0.69	88.55
Nuculana acuta	     0.00	     0.17	   0.58	   0.29	    0.68	89.23
Timoclea sp.	     0.00	     0.15	   0.51	   0.43	    0.59	89.82
Chione cancellata	     0.00	     0.10	   0.42	   0.29	    0.49	90.31

Groups M07  &  M12
Average dissimilarity = 65.81

	Group M07	Group M12	       	       	        	     
Species	 Av.Abund	 Av.Abund	Av.Diss	Diss/SD	Contrib%	Cum.%
Nucula proxima	     0.87	     2.94	   6.84	   1.31	   10.39	10.39
Tellina sp	     2.55	     1.71	   6.69	   1.40	   10.17	20.56
Parvilucina multilineata	     1.77	     1.93	   5.74	   1.23	    8.73	29.29
Macoma tenta	     1.55	     1.68	   5.59	   1.08	    8.49	37.78
Tagelus divisus	     0.82	     0.89	   3.26	   0.85	    4.96	42.74
Abra aequalis	     1.30	     1.38	   2.91	   1.32	    4.42	47.16
Chione sp.	     0.85	     0.95	   2.41	   1.21	    3.66	50.82
Divaricella quadrisulcata	     0.46	     0.37	   1.99	   0.75	    3.02	53.84
Mulinia lateralis	     0.68	     0.17	   1.98	   0.91	    3.01	56.85
Tellina versicolor	     0.21	     0.53	   1.95	   0.52	    2.96	59.81
Lyonsia floridana	     0.65	     0.48	   1.82	   1.17	    2.77	62.58
Lucina sp.	     0.45	     0.26	   1.74	   0.79	    2.64	65.21
Anadara sp.	     0.40	     0.33	   1.52	   0.93	    2.31	67.52
Crassinella lunulata	     0.28	     0.47	   1.51	   1.09	    2.29	69.81
Caryocorbula sp.	     0.32	     0.33	   1.34	   0.98	    2.04	71.85
Parvilucina sp.	     0.16	     0.19	   0.98	   0.32	    1.50	73.35
Amygdalum papyrium	     0.33	     0.07	   0.98	   0.62	    1.48	74.83
Bivalvia	     0.28	     0.09	   0.96	   0.53	    1.46	76.29
Corbula sp.	     0.23	     0.20	   0.95	   0.82	    1.44	77.74
Timoclea sp.	     0.25	     0.15	   0.91	   0.62	    1.38	79.12
Crassinella sp.	     0.21	     0.16	   0.91	   0.67	    1.38	80.50
Asthenothaerus hemphilli	     0.21	     0.08	   0.80	   0.58	    1.22	81.71
Lucinisca nassula	     0.11	     0.14	   0.66	   0.60	    1.00	82.71
Sphenia sp.	     0.20	     0.02	   0.65	   0.52	    0.99	83.70
Mactridae	     0.16	     0.06	   0.55	   0.52	    0.83	84.54
Chione cancellata	     0.08	     0.10	   0.50	   0.46	    0.76	85.29
Lucinidae	     0.16	     0.00	   0.49	   0.29	    0.74	86.03
Timoclea grus	     0.11	     0.07	   0.48	   0.44	    0.73	86.76
Nuculana acuta	     0.02	     0.17	   0.45	   0.31	    0.68	87.44
Sphenia antillensis	     0.09	     0.02	   0.33	   0.38	    0.51	87.94
Divaricella dentata	     0.09	     0.00	   0.33	   0.19	    0.51	88.45
Mysella sp.	     0.03	     0.07	   0.31	   0.35	    0.48	88.92
Anadara floridana	     0.08	     0.05	   0.31	   0.38	    0.48	89.40
Mytilopsis leucophaeata	     0.07	     0.02	   0.30	   0.38	    0.45	89.85
Divalinga quadrisculata	     0.06	     0.06	   0.28	   0.36	    0.43	90.28

Groups M08  &  M12
Average dissimilarity = 66.33

	Group M08	Group M12	       	       	        	     
Species	 Av.Abund	 Av.Abund	Av.Diss	Diss/SD	Contrib%	Cum.%
Nucula proxima	     1.08	     2.94	   6.79	   1.27	   10.23	10.23
Tagelus divisus	     1.96	     0.89	   5.48	   1.21	    8.26	18.49
Tellina sp	     1.03	     1.71	   5.42	   1.09	    8.18	26.67
Parvilucina multilineata	     1.08	     1.93	   5.07	   1.10	    7.65	34.32
Macoma tenta	     0.97	     1.68	   4.73	   1.15	    7.13	41.45
Abra aequalis	     2.05	     1.38	   3.98	   1.24	    6.01	47.46
Mulinia lateralis	     1.09	     0.17	   3.08	   1.39	    4.64	52.10
Caryocorbula sp.	     0.79	     0.33	   2.46	   1.06	    3.71	55.81
Chione sp.	     0.96	     0.95	   2.18	   1.20	    3.28	59.09
Corbula sp.	     0.69	     0.20	   2.04	   1.09	    3.07	62.16
Lyonsia floridana	     0.48	     0.48	   1.78	   1.11	    2.68	64.84
Lucina sp.	     0.34	     0.26	   1.67	   0.71	    2.51	67.35
Tellina versicolor	     0.06	     0.53	   1.60	   0.47	    2.41	69.77
Crassinella lunulata	     0.06	     0.47	   1.40	   1.02	    2.11	71.88
Divaricella quadrisulcata	     0.11	     0.37	   1.32	   0.62	    1.99	73.87
Lucinoma filosa	     0.39	     0.03	   1.28	   0.69	    1.93	75.80
Anadara sp.	     0.17	     0.33	   1.12	   0.72	    1.69	77.49
Nuculana acuta	     0.20	     0.17	   0.92	   0.52	    1.39	78.88
Asthenothaerus hemphilli	     0.25	     0.08	   0.88	   0.58	    1.32	80.20
Bivalvia	     0.18	     0.09	   0.78	   0.60	    1.18	81.38
Amygdalum papyrium	     0.21	     0.07	   0.69	   0.63	    1.04	82.42
Crassinella sp.	     0.08	     0.16	   0.67	   0.59	    1.00	83.43
Parvilucina sp.	     0.02	     0.19	   0.66	   0.28	    1.00	84.43
Corbula contracta	     0.22	     0.02	   0.62	   0.37	    0.94	85.37
Tellinidae	     0.16	     0.03	   0.56	   0.46	    0.84	86.21
Lucinisca nassula	     0.02	     0.14	   0.53	   0.50	    0.80	87.00
Anadara floridana	     0.12	     0.05	   0.52	   0.47	    0.78	87.78
Timoclea sp.	     0.06	     0.15	   0.50	   0.52	    0.75	88.53
Mactridae	     0.09	     0.06	   0.41	   0.49	    0.62	89.15
Tellina mera	     0.05	     0.09	   0.41	   0.28	    0.61	89.76
Corbula chittyana	     0.12	     0.00	   0.38	   0.20	    0.57	90.34

Groups M09  &  M12
Average dissimilarity = 67.41

	Group M09	Group M12	       	       	        	     
Species	 Av.Abund	 Av.Abund	Av.Diss	Diss/SD	Contrib%	Cum.%
Nucula proxima	     0.97	     2.94	   7.44	   1.38	   11.03	11.03
Tellina sp	     1.06	     1.71	   6.05	   1.18	    8.98	20.01
Parvilucina multilineata	     0.62	     1.93	   5.88	   1.06	    8.72	28.73
Macoma tenta	     0.70	     1.68	   5.15	   1.13	    7.63	36.37
Tagelus divisus	     1.05	     0.89	   4.24	   0.98	    6.30	42.66
Chione sp.	     1.71	     0.95	   4.12	   1.41	    6.11	48.77
Abra aequalis	     0.87	     1.38	   3.39	   1.29	    5.03	53.81
Lucina sp.	     0.59	     0.26	   2.42	   0.85	    3.59	57.40
Lyonsia floridana	     0.44	     0.48	   1.93	   1.12	    2.86	60.26
Tellina versicolor	     0.08	     0.53	   1.83	   0.50	    2.72	62.98
Corbula sp.	     0.51	     0.20	   1.80	   1.05	    2.67	65.64
Caryocorbula sp.	     0.40	     0.33	   1.75	   1.00	    2.59	68.23
Chione cancellata	     0.38	     0.10	   1.62	   0.48	    2.41	70.64
Anadara sp.	     0.31	     0.33	   1.61	   0.85	    2.39	73.03
Crassinella lunulata	     0.09	     0.47	   1.55	   1.06	    2.30	75.33
Divaricella quadrisulcata	     0.11	     0.37	   1.48	   0.65	    2.19	77.53
Mulinia lateralis	     0.32	     0.17	   1.39	   0.82	    2.06	79.59
Lucinisca nassula	     0.10	     0.14	   0.75	   0.63	    1.12	80.71
Parvilucina sp.	     0.00	     0.19	   0.68	   0.26	    1.02	81.72
Crassinella sp.	     0.04	     0.16	   0.68	   0.55	    1.01	82.73
Timoclea sp.	     0.09	     0.15	   0.65	   0.58	    0.96	83.69
Bivalvia	     0.11	     0.09	   0.63	   0.55	    0.93	84.62
Asthenothaerus hemphilli	     0.12	     0.08	   0.60	   0.49	    0.89	85.51
Anadara floridana	     0.12	     0.05	   0.52	   0.38	    0.77	86.27
Nuculana acuta	     0.02	     0.17	   0.51	   0.31	    0.75	87.03
Mysella sp.	     0.07	     0.07	   0.50	   0.43	    0.74	87.77
Sphenia sp.	     0.13	     0.02	   0.50	   0.53	    0.74	88.50
Amygdalum papyrium	     0.04	     0.07	   0.32	   0.45	    0.47	88.97
Tellina mera	     0.00	     0.09	   0.31	   0.21	    0.46	89.43
Corbula contracta	     0.07	     0.02	   0.30	   0.30	    0.45	89.87
Timoclea grus	     0.02	     0.07	   0.28	   0.33	    0.42	90.29

Groups M10  &  M12
Average dissimilarity = 76.77

	Group M10	Group M12	       	       	        	     
Species	 Av.Abund	 Av.Abund	Av.Diss	Diss/SD	Contrib%	Cum.%
Nucula proxima	     0.71	     2.94	   9.18	   1.42	   11.95	11.95
Parvilucina multilineata	     0.00	     1.93	   6.78	   0.99	    8.83	20.79
Tellina sp	     0.43	     1.71	   6.70	   0.99	    8.72	29.51
Macoma tenta	     1.04	     1.68	   5.60	   1.15	    7.30	36.81
Mysella sp.	     1.26	     0.07	   4.87	   0.85	    6.35	43.16
Abra aequalis	     0.35	     1.38	   4.16	   1.35	    5.41	48.58
Tagelus divisus	     0.82	     0.89	   4.08	   0.90	    5.32	53.89
Chione sp.	     1.52	     0.95	   3.79	   1.33	    4.94	58.83
Caryocorbula sp.	     0.86	     0.33	   3.12	   1.12	    4.07	62.90
Mulinia lateralis	     0.79	     0.17	   3.02	   0.95	    3.93	66.83
Tellina versicolor	     0.07	     0.53	   1.94	   0.47	    2.52	69.35
Lyonsia floridana	     0.10	     0.48	   1.87	   0.91	    2.44	71.79
Crassinella lunulata	     0.00	     0.47	   1.70	   1.02	    2.21	74.00
Amygdalum papyrium	     0.38	     0.07	   1.56	   0.46	    2.03	76.02
Mysella planulata	     0.44	     0.04	   1.54	   0.45	    2.01	78.03
Corbula sp.	     0.30	     0.20	   1.46	   0.80	    1.90	79.93
Divaricella quadrisulcata	     0.00	     0.37	   1.43	   0.54	    1.86	81.80
Lucina sp.	     0.05	     0.26	   1.14	   0.50	    1.49	83.29
Anadara sp.	     0.03	     0.33	   1.04	   0.62	    1.35	84.64
Bivalvia	     0.18	     0.09	   1.02	   0.38	    1.33	85.97
Parvilucina sp.	     0.00	     0.19	   0.75	   0.26	    0.97	86.95
Crassinella sp.	     0.00	     0.16	   0.65	   0.48	    0.85	87.79
Nuculana acuta	     0.05	     0.17	   0.64	   0.37	    0.84	88.63
Lucinisca nassula	     0.00	     0.14	   0.63	   0.47	    0.82	89.46
Asthenothaerus hemphilli	     0.11	     0.08	   0.58	   0.53	    0.75	90.21

Groups M11  &  M12
Average dissimilarity = 75.22

	Group M11	Group M12	       	       	        	     
Species	 Av.Abund	 Av.Abund	Av.Diss	Diss/SD	Contrib%	Cum.%
Nucula proxima	     0.95	     2.94	   9.41	   1.36	   12.51	12.51
Parvilucina multilineata	     0.00	     1.93	   7.25	   0.99	    9.64	22.15
Tellina sp	     0.53	     1.71	   7.19	   1.01	    9.56	31.71
Macoma tenta	     0.69	     1.68	   5.78	   1.11	    7.68	39.39
Tagelus divisus	     0.86	     0.89	   4.68	   0.86	    6.22	45.61
Abra aequalis	     0.49	     1.38	   4.47	   1.34	    5.95	51.56
Chione sp.	     1.24	     0.95	   4.16	   1.16	    5.53	57.09
Mulinia lateralis	     0.54	     0.17	   2.33	   0.91	    3.10	60.19
Lyonsia floridana	     0.21	     0.48	   2.15	   0.95	    2.86	63.05
Tellina versicolor	     0.07	     0.53	   2.12	   0.49	    2.82	65.86
Caryocorbula sp.	     0.39	     0.33	   2.03	   1.01	    2.70	68.56
Corbula sp.	     0.47	     0.20	   2.01	   0.95	    2.67	71.23
Crassinella lunulata	     0.00	     0.47	   1.82	   1.01	    2.41	73.64
Divaricella quadrisulcata	     0.03	     0.37	   1.59	   0.56	    2.12	75.76
Mysella sp.	     0.35	     0.07	   1.53	   0.57	    2.03	77.79
Lucina sp.	     0.09	     0.26	   1.28	   0.52	    1.70	79.49
Anadara sp.	     0.00	     0.33	   1.04	   0.59	    1.38	80.87
Nuculana acuta	     0.12	     0.17	   0.90	   0.43	    1.20	82.08
Parvilucina sp.	     0.00	     0.19	   0.81	   0.25	    1.07	83.15
Chione cancellata	     0.11	     0.10	   0.80	   0.38	    1.06	84.21
Amygdalum papyrium	     0.14	     0.07	   0.79	   0.35	    1.05	85.26
Crassinella sp.	     0.00	     0.16	   0.70	   0.48	    0.93	86.19
Lucinisca nassula	     0.00	     0.14	   0.70	   0.46	    0.93	87.12
Bivalvia	     0.06	     0.09	   0.63	   0.47	    0.84	87.96
Mysella planulata	     0.14	     0.04	   0.63	   0.43	    0.84	88.80
Sphenia sp.	     0.11	     0.02	   0.50	   0.44	    0.67	89.47
Timoclea sp.	     0.00	     0.15	   0.46	   0.43	    0.61	90.08

Groups M02  &  M13
Average dissimilarity = 94.04

	Group M02	Group M13	       	       	        	     
Species	 Av.Abund	 Av.Abund	Av.Diss	Diss/SD	Contrib%	Cum.%
Mytilopsis leucophaeata	     4.21	     0.00	  16.35	   0.90	   17.38	17.38
Mulinia lateralis	     4.29	     0.30	  13.68	   0.76	   14.54	31.93
Rangia cuneata	     2.40	     0.00	  12.63	   1.06	   13.43	45.35
Tellina sp	     1.17	     0.57	   5.79	   1.05	    6.15	51.51
Macoma tenta	     0.38	     0.62	   4.11	   0.82	    4.37	55.87
Chione sp.	     0.00	     0.59	   3.64	   0.84	    3.87	59.74
Nucula proxima	     0.00	     0.56	   3.37	   0.85	    3.58	63.33
Abra aequalis	     0.00	     0.42	   2.33	   0.71	    2.47	65.80
Amygdalum papyrium	     0.28	     0.23	   1.94	   0.62	    2.07	67.87
Tagelus divisus	     0.06	     0.33	   1.87	   0.51	    1.99	69.86
Lyonsia floridana	     0.23	     0.21	   1.81	   0.69	    1.92	71.78
Lucina sp.	     0.06	     0.24	   1.78	   0.49	    1.89	73.67
Anadara sp.	     0.00	     0.32	   1.71	   0.62	    1.82	75.49
Parvilucina multilineata	     0.02	     0.25	   1.53	   0.60	    1.62	77.11
Mactridae	     0.28	     0.02	   1.48	   0.38	    1.57	78.69
Sphenia sp.	     0.00	     0.25	   1.32	   0.54	    1.41	80.09
Polymesoda caroliniana	     0.32	     0.00	   1.21	   0.35	    1.29	81.38
Polymesoda sp.	     0.10	     0.00	   1.14	   0.20	    1.21	82.59
Chione cancellata	     0.00	     0.17	   1.08	   0.36	    1.15	83.74
Caryocorbula sp.	     0.00	     0.18	   1.07	   0.52	    1.14	84.88
Tagelus plebeius	     0.25	     0.00	   1.07	   0.51	    1.13	86.01
Mysella sp.	     0.02	     0.14	   0.82	   0.45	    0.87	86.88
Macoma cerina	     0.00	     0.14	   0.76	   0.23	    0.81	87.69
Tellinidae	     0.12	     0.02	   0.76	   0.30	    0.81	88.50
Brachidontes exustus	     0.10	     0.02	   0.69	   0.38	    0.74	89.24
Anadara floridana	     0.00	     0.12	   0.62	   0.27	    0.66	89.90
Bivalvia	     0.02	     0.06	   0.60	   0.31	    0.64	90.54

Groups M03  &  M13
Average dissimilarity = 93.88

	Group M03	Group M13	       	       	        	     
Species	 Av.Abund	 Av.Abund	Av.Diss	Diss/SD	Contrib%	Cum.%
Mulinia lateralis	     2.92	     0.30	  24.33	   1.56	   25.92	25.92
Mytilopsis leucophaeata	     0.95	     0.00	   6.65	   0.51	    7.08	33.00
Chione sp.	     0.00	     0.59	   6.08	   0.94	    6.47	39.48
Nucula proxima	     0.00	     0.56	   5.52	   0.96	    5.88	45.36
Macoma tenta	     0.05	     0.62	   5.34	   0.78	    5.69	51.05
Tellina sp	     0.10	     0.57	   5.11	   0.82	    5.45	56.50
Abra aequalis	     0.00	     0.42	   3.66	   0.79	    3.90	60.40
Anadara sp.	     0.00	     0.32	   2.64	   0.68	    2.82	63.22
Lucina sp.	     0.00	     0.24	   2.64	   0.47	    2.81	66.03
Tagelus divisus	     0.00	     0.33	   2.61	   0.50	    2.78	68.81
Parvilucina multilineata	     0.00	     0.25	   2.49	   0.66	    2.65	71.46
Sphenia sp.	     0.00	     0.25	   2.10	   0.59	    2.24	73.70
Amygdalum papyrium	     0.00	     0.23	   2.00	   0.53	    2.13	75.84
Lyonsia floridana	     0.00	     0.21	   2.00	   0.60	    2.13	77.96
Chione cancellata	     0.00	     0.17	   1.79	   0.40	    1.90	79.87
Caryocorbula sp.	     0.00	     0.18	   1.76	   0.57	    1.88	81.75
Mysella sp.	     0.00	     0.14	   1.27	   0.48	    1.35	83.10
Macoma cerina	     0.00	     0.14	   1.20	   0.25	    1.27	84.37
Corbula sp.	     0.05	     0.05	   1.06	   0.34	    1.13	85.50
Anadara floridana	     0.00	     0.12	   0.96	   0.29	    1.02	86.52
Timoclea sp.	     0.00	     0.07	   0.95	   0.24	    1.01	87.53
Laevicardium mortoni	     0.00	     0.08	   0.73	   0.35	    0.78	88.31
Carditamera floridana	     0.00	     0.07	   0.71	   0.29	    0.75	89.07
ostreidae	     0.00	     0.07	   0.64	   0.35	    0.68	89.75
Trachycardium sp.	     0.00	     0.06	   0.63	   0.27	    0.68	90.43

Groups M04  &  M13
Average dissimilarity = 92.83

	Group M04	Group M13	       	       	        	     
Species	 Av.Abund	 Av.Abund	Av.Diss	Diss/SD	Contrib%	Cum.%
Mulinia lateralis	     4.11	     0.30	  27.85	   1.34	   30.00	30.00
Chione sp.	     0.00	     0.59	   6.10	   0.90	    6.57	36.56
Nucula proxima	     0.00	     0.56	   5.52	   0.92	    5.95	42.51
Macoma tenta	     0.15	     0.62	   5.37	   0.78	    5.79	48.30
Tellina sp	     0.11	     0.57	   5.09	   0.79	    5.48	53.78
Tagelus divisus	     0.18	     0.33	   3.69	   0.67	    3.97	57.75
Abra aequalis	     0.00	     0.42	   3.64	   0.77	    3.93	61.68
Lucina sp.	     0.00	     0.24	   2.65	   0.46	    2.85	64.53
Anadara sp.	     0.00	     0.32	   2.63	   0.67	    2.83	67.35
Parvilucina multilineata	     0.03	     0.25	   2.54	   0.66	    2.74	70.09
Sphenia sp.	     0.00	     0.25	   2.10	   0.58	    2.26	72.35
Amygdalum papyrium	     0.00	     0.23	   1.99	   0.52	    2.15	74.50
Lyonsia floridana	     0.00	     0.21	   1.99	   0.58	    2.15	76.65
Chione cancellata	     0.00	     0.17	   1.79	   0.39	    1.92	78.57
Caryocorbula sp.	     0.00	     0.18	   1.77	   0.55	    1.90	80.47
Mysella sp.	     0.00	     0.14	   1.27	   0.47	    1.36	81.84
Macoma cerina	     0.00	     0.14	   1.19	   0.25	    1.28	83.12
Timoclea sp.	     0.00	     0.07	   0.96	   0.23	    1.03	84.15
Anadara floridana	     0.00	     0.12	   0.95	   0.29	    1.02	85.18
Corbicula sp.	     0.06	     0.00	   0.74	   0.20	    0.80	85.98
Laevicardium mortoni	     0.00	     0.08	   0.73	   0.35	    0.79	86.76
Carditamera floridana	     0.00	     0.07	   0.71	   0.28	    0.77	87.53
ostreidae	     0.00	     0.07	   0.64	   0.34	    0.69	88.22
Trachycardium sp.	     0.00	     0.06	   0.64	   0.26	    0.69	88.90
Anomalocardia auberiana	     0.00	     0.07	   0.61	   0.35	    0.65	89.56
Corbula sp.	     0.00	     0.05	   0.57	   0.28	    0.62	90.17

Groups M05  &  M13
Average dissimilarity = 93.78

	Group M05	Group M13	       	       	        	     
Species	 Av.Abund	 Av.Abund	Av.Diss	Diss/SD	Contrib%	Cum.%
Mulinia lateralis	     1.50	     0.30	  15.20	   1.14	   16.20	16.20
Chione sp.	     0.00	     0.59	   7.60	   0.94	    8.10	24.31
Nucula proxima	     0.00	     0.56	   6.81	   0.97	    7.26	31.57
Macoma tenta	     0.04	     0.62	   6.38	   0.78	    6.80	38.37
Tellina sp	     0.00	     0.57	   5.92	   0.76	    6.31	44.68
Tagelus divisus	     0.21	     0.33	   4.70	   0.73	    5.02	49.70
Abra aequalis	     0.00	     0.42	   4.38	   0.81	    4.67	54.37
Lucina sp.	     0.00	     0.24	   3.31	   0.47	    3.53	57.90
Parvilucina multilineata	     0.03	     0.25	   3.19	   0.70	    3.40	61.30
Anadara sp.	     0.00	     0.32	   3.12	   0.70	    3.33	64.63
Sphenia sp.	     0.00	     0.25	   2.55	   0.60	    2.72	67.35
Lyonsia floridana	     0.00	     0.21	   2.43	   0.61	    2.59	69.94
Amygdalum papyrium	     0.00	     0.23	   2.40	   0.54	    2.56	72.50
Chione cancellata	     0.00	     0.17	   2.20	   0.41	    2.35	74.85
Caryocorbula sp.	     0.00	     0.18	   2.18	   0.57	    2.33	77.18
Mysella sp.	     0.00	     0.14	   1.54	   0.48	    1.64	78.81
Macoma cerina	     0.00	     0.14	   1.43	   0.26	    1.52	80.34
Timoclea sp.	     0.00	     0.07	   1.25	   0.24	    1.33	81.66
Anadara floridana	     0.00	     0.12	   1.13	   0.29	    1.21	82.87
Tellinidae	     0.04	     0.02	   0.94	   0.29	    1.00	83.87
Carditamera floridana	     0.00	     0.07	   0.89	   0.29	    0.95	84.82
Laevicardium mortoni	     0.00	     0.08	   0.89	   0.36	    0.95	85.77
Trachycardium sp.	     0.00	     0.06	   0.80	   0.27	    0.85	86.62
ostreidae	     0.00	     0.07	   0.78	   0.34	    0.83	87.44
Mytilopsis leucophaeata	     0.05	     0.00	   0.75	   0.29	    0.80	88.24
Anomalocardia auberiana	     0.00	     0.07	   0.73	   0.36	    0.78	89.02
Corbula sp.	     0.00	     0.05	   0.72	   0.29	    0.77	89.79
Bivalvia	     0.00	     0.06	   0.64	   0.27	    0.68	90.47

Groups M06  &  M13
Average dissimilarity = 81.86

	Group M06	Group M13	       	       	        	     
Species	 Av.Abund	 Av.Abund	Av.Diss	Diss/SD	Contrib%	Cum.%
Mulinia lateralis	     1.51	     0.30	  10.59	   1.07	   12.94	12.94
Tagelus divisus	     0.83	     0.33	   6.63	   0.98	    8.10	21.04
Macoma tenta	     0.29	     0.62	   5.38	   0.86	    6.57	27.61
Tellina sp	     0.34	     0.57	   5.26	   0.93	    6.42	34.03
Nucula proxima	     0.08	     0.56	   5.07	   0.99	    6.19	40.22
Chione sp.	     0.18	     0.59	   4.94	   0.96	    6.03	46.25
Abra aequalis	     0.34	     0.42	   4.09	   0.95	    5.00	51.25
Lucina sp.	     0.13	     0.24	   2.93	   0.58	    3.58	54.83
Amygdalum papyrium	     0.19	     0.23	   2.75	   0.68	    3.36	58.19
Parvilucina multilineata	     0.16	     0.25	   2.72	   0.82	    3.32	61.51
Anadara sp.	     0.05	     0.32	   2.59	   0.72	    3.16	64.67
Lyonsia floridana	     0.11	     0.21	   2.28	   0.66	    2.79	67.46
Caryocorbula sp.	     0.14	     0.18	   2.17	   0.72	    2.66	70.11
Sphenia sp.	     0.07	     0.25	   2.12	   0.66	    2.59	72.70
Chione cancellata	     0.00	     0.17	   1.62	   0.40	    1.98	74.68
Corbula sp.	     0.17	     0.05	   1.55	   0.49	    1.89	76.57
Mysella sp.	     0.00	     0.14	   1.16	   0.48	    1.42	77.99
Macoma cerina	     0.00	     0.14	   1.10	   0.25	    1.34	79.33
Tagelus sp.	     0.07	     0.04	   0.93	   0.26	    1.13	80.46
Bivalvia	     0.05	     0.06	   0.91	   0.39	    1.11	81.57
Anadara floridana	     0.00	     0.12	   0.89	   0.29	    1.08	82.66
Lucinoma filosa	     0.10	     0.02	   0.85	   0.32	    1.04	83.69
Timoclea sp.	     0.00	     0.07	   0.83	   0.24	    1.01	84.71
Laevicardium mortoni	     0.00	     0.08	   0.67	   0.36	    0.82	85.53
Carditamera floridana	     0.00	     0.07	   0.64	   0.29	    0.78	86.30
ostreidae	     0.00	     0.07	   0.58	   0.35	    0.71	87.02
Trachycardium sp.	     0.00	     0.06	   0.57	   0.27	    0.70	87.71
Anomalocardia auberiana	     0.00	     0.07	   0.56	   0.36	    0.68	88.39
Brachidontes sp.	     0.03	     0.05	   0.55	   0.28	    0.67	89.07
Macoma sp.	     0.06	     0.00	   0.54	   0.19	    0.66	89.72
Tellinidae	     0.02	     0.02	   0.53	   0.27	    0.64	90.37

Groups M07  &  M13
Average dissimilarity = 76.88

	Group M07	Group M13	       	       	        	     
Species	 Av.Abund	 Av.Abund	Av.Diss	Diss/SD	Contrib%	Cum.%
Tellina sp	     2.55	     0.57	   9.04	   1.42	   11.76	11.76
Parvilucina multilineata	     1.77	     0.25	   6.95	   1.18	    9.04	20.79
Macoma tenta	     1.55	     0.62	   6.06	   0.90	    7.88	28.67
Abra aequalis	     1.30	     0.42	   4.10	   1.58	    5.33	34.00
Tagelus divisus	     0.82	     0.33	   3.71	   0.87	    4.83	38.83
Chione sp.	     0.85	     0.59	   3.30	   1.19	    4.29	43.12
Nucula proxima	     0.87	     0.56	   3.16	   1.24	    4.10	47.23
Mulinia lateralis	     0.68	     0.30	   2.91	   0.96	    3.79	51.01
Lyonsia floridana	     0.65	     0.21	   2.47	   1.22	    3.22	54.23
Lucina sp.	     0.45	     0.24	   2.37	   0.80	    3.09	57.32
Anadara sp.	     0.40	     0.32	   2.09	   0.96	    2.72	60.04
Divaricella quadrisulcata	     0.46	     0.05	   2.01	   0.56	    2.61	62.65
Amygdalum papyrium	     0.33	     0.23	   1.78	   0.75	    2.31	64.96
Sphenia sp.	     0.20	     0.25	   1.51	   0.76	    1.96	66.92
Caryocorbula sp.	     0.32	     0.18	   1.48	   0.90	    1.92	68.85
Tellina versicolor	     0.21	     0.04	   1.20	   0.34	    1.57	70.41
Bivalvia	     0.28	     0.06	   1.19	   0.49	    1.55	71.96
Crassinella lunulata	     0.28	     0.05	   1.15	   0.65	    1.50	73.46
Timoclea sp.	     0.25	     0.07	   1.15	   0.53	    1.49	74.96
Corbula sp.	     0.23	     0.05	   1.03	   0.69	    1.33	76.29
Chione cancellata	     0.08	     0.17	   1.02	   0.52	    1.33	77.62
Asthenothaerus hemphilli	     0.21	     0.00	   0.99	   0.49	    1.29	78.91
Crassinella sp.	     0.21	     0.02	   0.82	   0.50	    1.07	79.98
Lucinidae	     0.16	     0.00	   0.71	   0.28	    0.93	80.91
Anadara floridana	     0.08	     0.12	   0.71	   0.41	    0.92	81.83
Parvilucina sp.	     0.16	     0.03	   0.71	   0.23	    0.92	82.74
Mysella sp.	     0.03	     0.14	   0.69	   0.52	    0.90	83.64
Mactridae	     0.16	     0.02	   0.63	   0.48	    0.82	84.46
Macoma cerina	     0.00	     0.14	   0.58	   0.25	    0.75	85.22
Divaricella dentata	     0.09	     0.00	   0.51	   0.19	    0.67	85.88
Sphenia antillensis	     0.09	     0.02	   0.49	   0.38	    0.64	86.52
Laevicardium mortoni	     0.05	     0.08	   0.48	   0.41	    0.62	87.15
Veneridae	     0.10	     0.02	   0.47	   0.44	    0.62	87.76
Timoclea grus	     0.11	     0.00	   0.43	   0.34	    0.56	88.32
Lucina nassula	     0.09	     0.00	   0.39	   0.34	    0.50	88.83
Lucinisca nassula	     0.11	     0.00	   0.36	   0.37	    0.47	89.30
Mytilopsis leucophaeata	     0.07	     0.00	   0.35	   0.32	    0.45	89.75
Carditamera floridana	     0.00	     0.07	   0.31	   0.29	    0.40	90.15

Groups M08  &  M13
Average dissimilarity = 75.38

	Group M08	Group M13	       	       	        	     
Species	 Av.Abund	 Av.Abund	Av.Diss	Diss/SD	Contrib%	Cum.%
Tagelus divisus	     1.96	     0.33	   7.69	   1.32	   10.20	10.20
Abra aequalis	     2.05	     0.42	   7.52	   1.63	    9.98	20.18
Tellina sp	     1.03	     0.57	   4.48	   1.10	    5.94	26.12
Mulinia lateralis	     1.09	     0.30	   4.29	   1.35	    5.69	31.80
Macoma tenta	     0.97	     0.62	   4.24	   1.10	    5.63	37.43
Parvilucina multilineata	     1.08	     0.25	   4.12	   1.47	    5.47	42.90
Nucula proxima	     1.08	     0.56	   3.65	   1.13	    4.84	47.74
Caryocorbula sp.	     0.79	     0.18	   3.33	   0.96	    4.42	52.16
Chione sp.	     0.96	     0.59	   3.26	   1.26	    4.33	56.49
Corbula sp.	     0.69	     0.05	   2.87	   1.08	    3.81	60.30
Lucina sp.	     0.34	     0.24	   2.35	   0.71	    3.12	63.42
Lyonsia floridana	     0.48	     0.21	   2.18	   1.00	    2.90	66.32
Lucinoma filosa	     0.39	     0.02	   1.87	   0.64	    2.48	68.80
Anadara sp.	     0.17	     0.32	   1.67	   0.78	    2.22	71.02
Amygdalum papyrium	     0.21	     0.23	   1.49	   0.74	    1.97	72.99
Sphenia sp.	     0.09	     0.25	   1.19	   0.66	    1.58	74.57
Asthenothaerus hemphilli	     0.25	     0.00	   1.08	   0.49	    1.43	76.00
Anadara floridana	     0.12	     0.12	   1.03	   0.51	    1.36	77.36
Bivalvia	     0.18	     0.06	   1.02	   0.55	    1.35	78.71
Chione cancellata	     0.02	     0.17	   0.89	   0.43	    1.18	79.89
Macoma cerina	     0.08	     0.14	   0.88	   0.34	    1.17	81.06
Nuculana acuta	     0.20	     0.00	   0.80	   0.46	    1.06	82.12
Tellinidae	     0.16	     0.02	   0.77	   0.46	    1.02	83.13
Corbula contracta	     0.22	     0.00	   0.76	   0.34	    1.01	84.14
Mysella sp.	     0.02	     0.14	   0.68	   0.52	    0.90	85.04
Divaricella quadrisulcata	     0.11	     0.05	   0.61	   0.44	    0.81	85.85
Timoclea sp.	     0.06	     0.07	   0.57	   0.36	    0.76	86.61
Corbula chittyana	     0.12	     0.00	   0.54	   0.20	    0.71	87.32
Macoma sp.	     0.13	     0.00	   0.51	   0.29	    0.68	88.00
Laevicardium mortoni	     0.03	     0.08	   0.46	   0.41	    0.61	88.62
Mactridae	     0.09	     0.02	   0.45	   0.46	    0.59	89.21
Crassinella sp.	     0.08	     0.02	   0.43	   0.41	    0.58	89.79
Tellina versicolor	     0.06	     0.04	   0.43	   0.34	    0.57	90.36

Groups M09  &  M13
Average dissimilarity = 72.66

	Group M09	Group M13	       	       	        	     
Species	 Av.Abund	 Av.Abund	Av.Diss	Diss/SD	Contrib%	Cum.%
Chione sp.	     1.71	     0.59	   6.96	   1.49	    9.58	 9.58
Tagelus divisus	     1.05	     0.33	   5.50	   1.03	    7.57	17.15
Tellina sp	     1.06	     0.57	   5.33	   1.18	    7.34	24.49
Macoma tenta	     0.70	     0.62	   4.31	   1.03	    5.94	30.43
Abra aequalis	     0.87	     0.42	   3.95	   1.27	    5.44	35.87
Nucula proxima	     0.97	     0.56	   3.79	   1.23	    5.21	41.08
Lucina sp.	     0.59	     0.24	   3.53	   0.87	    4.86	45.94
Parvilucina multilineata	     0.62	     0.25	   3.32	   1.04	    4.57	50.51
Chione cancellata	     0.38	     0.17	   2.78	   0.55	    3.83	54.34
Corbula sp.	     0.51	     0.05	   2.71	   1.04	    3.72	58.06
Anadara sp.	     0.31	     0.32	   2.42	   0.88	    3.33	61.39
Mulinia lateralis	     0.32	     0.30	   2.41	   0.84	    3.32	64.71
Lyonsia floridana	     0.44	     0.21	   2.37	   1.00	    3.27	67.97
Caryocorbula sp.	     0.40	     0.18	   2.30	   0.89	    3.17	71.14
Sphenia sp.	     0.13	     0.25	   1.51	   0.75	    2.08	73.23
Amygdalum papyrium	     0.04	     0.23	   1.27	   0.60	    1.75	74.97
Anadara floridana	     0.12	     0.12	   1.08	   0.45	    1.48	76.46
Mysella sp.	     0.07	     0.14	   0.99	   0.59	    1.37	77.82
Timoclea sp.	     0.09	     0.07	   0.84	   0.44	    1.16	78.98
Bivalvia	     0.11	     0.06	   0.76	   0.52	    1.05	80.03
Divaricella quadrisulcata	     0.11	     0.05	   0.71	   0.53	    0.98	81.01
Macoma cerina	     0.00	     0.14	   0.70	   0.25	    0.96	81.97
Asthenothaerus hemphilli	     0.12	     0.00	   0.64	   0.36	    0.87	82.84
Crassinella lunulata	     0.09	     0.05	   0.63	   0.49	    0.87	83.71
Tellina versicolor	     0.08	     0.04	   0.61	   0.39	    0.83	84.55
Lucinisca nassula	     0.10	     0.00	   0.53	   0.45	    0.73	85.28
Laevicardium mortoni	     0.02	     0.08	   0.49	   0.41	    0.68	85.95
Nucula sp.	     0.04	     0.03	   0.41	   0.28	    0.57	86.52
Trachycardium sp.	     0.02	     0.06	   0.41	   0.33	    0.57	87.09
Thracia	     0.04	     0.05	   0.41	   0.32	    0.56	87.65
Laevicardium sp.	     0.05	     0.02	   0.38	   0.32	    0.52	88.17
Carditamera floridana	     0.00	     0.07	   0.37	   0.30	    0.52	88.69
Crassinella sp.	     0.04	     0.02	   0.37	   0.33	    0.51	89.19
ostreidae	     0.00	     0.07	   0.36	   0.37	    0.50	89.69
Anomalocardia auberiana	     0.00	     0.07	   0.35	   0.37	    0.49	90.18

Groups M10  &  M13
Average dissimilarity = 76.91

	Group M10	Group M13	       	       	        	     
Species	 Av.Abund	 Av.Abund	Av.Diss	Diss/SD	Contrib%	Cum.%
Mysella sp.	     1.26	     0.14	   7.69	   0.89	   10.00	10.00
Chione sp.	     1.52	     0.59	   6.96	   1.39	    9.05	19.05
Macoma tenta	     1.04	     0.62	   6.14	   1.01	    7.98	27.03
Mulinia lateralis	     0.79	     0.30	   4.88	   0.95	    6.34	33.37
Tagelus divisus	     0.82	     0.33	   4.81	   0.94	    6.25	39.62
Caryocorbula sp.	     0.86	     0.18	   4.58	   1.04	    5.96	45.58
Nucula proxima	     0.71	     0.56	   4.19	   1.12	    5.45	51.03
Tellina sp	     0.43	     0.57	   3.92	   0.96	    5.10	56.13
Amygdalum papyrium	     0.38	     0.23	   3.07	   0.60	    3.99	60.12
Abra aequalis	     0.35	     0.42	   2.84	   0.99	    3.69	63.81
Mysella planulata	     0.44	     0.06	   2.32	   0.47	    3.02	66.82
Corbula sp.	     0.30	     0.05	   1.92	   0.68	    2.49	69.31
Anadara sp.	     0.03	     0.32	   1.84	   0.71	    2.40	71.71
Lucina sp.	     0.05	     0.24	   1.75	   0.55	    2.27	73.98
Sphenia sp.	     0.14	     0.25	   1.71	   0.70	    2.22	76.20
Lyonsia floridana	     0.10	     0.21	   1.58	   0.69	    2.05	78.25
Parvilucina multilineata	     0.00	     0.25	   1.56	   0.67	    2.02	80.27
Bivalvia	     0.18	     0.06	   1.44	   0.33	    1.87	82.14
Chione cancellata	     0.05	     0.17	   1.31	   0.46	    1.71	83.85
Macoma cerina	     0.00	     0.14	   0.79	   0.25	    1.03	84.88
Anadara floridana	     0.00	     0.12	   0.65	   0.29	    0.84	85.72
Tellina versicolor	     0.07	     0.04	   0.59	   0.29	    0.76	86.48
Asthenothaerus hemphilli	     0.11	     0.00	   0.59	   0.39	    0.76	87.25
Laevicardium mortoni	     0.03	     0.08	   0.54	   0.41	    0.71	87.95
Tagelus sp.	     0.05	     0.04	   0.54	   0.29	    0.70	88.66
Timoclea sp.	     0.00	     0.07	   0.52	   0.26	    0.68	89.34
Carditamera floridana	     0.00	     0.07	   0.43	   0.29	    0.56	89.90
Brachidontes sp.	     0.03	     0.05	   0.43	   0.30	    0.55	90.46

Groups M11  &  M13
Average dissimilarity = 76.69

	Group M11	Group M13	       	       	        	     
Species	 Av.Abund	 Av.Abund	Av.Diss	Diss/SD	Contrib%	Cum.%
Chione sp.	     1.24	     0.59	   7.03	   1.16	    9.16	 9.16
Nucula proxima	     0.95	     0.56	   5.96	   0.87	    7.77	16.93
Tagelus divisus	     0.86	     0.33	   5.73	   0.79	    7.47	24.40
Tellina sp	     0.53	     0.57	   5.11	   0.96	    6.67	31.07
Macoma tenta	     0.69	     0.62	   5.06	   1.03	    6.60	37.67
Mulinia lateralis	     0.54	     0.30	   4.12	   0.90	    5.37	43.04
Abra aequalis	     0.49	     0.42	   3.91	   1.02	    5.09	48.13
Caryocorbula sp.	     0.39	     0.18	   3.13	   0.87	    4.09	52.22
Corbula sp.	     0.47	     0.05	   3.09	   0.89	    4.03	56.25
Mysella sp.	     0.35	     0.14	   2.61	   0.66	    3.41	59.66
Amygdalum papyrium	     0.14	     0.23	   2.23	   0.55	    2.91	62.57
Lyonsia floridana	     0.21	     0.21	   2.19	   0.76	    2.85	65.42
Lucina sp.	     0.09	     0.24	   2.05	   0.55	    2.68	68.10
Anadara sp.	     0.00	     0.32	   1.99	   0.68	    2.59	70.69
Sphenia sp.	     0.11	     0.25	   1.90	   0.70	    2.47	73.16
Chione cancellata	     0.11	     0.17	   1.86	   0.47	    2.43	75.59
Parvilucina multilineata	     0.00	     0.25	   1.77	   0.66	    2.30	77.89
Mysella planulata	     0.14	     0.06	   1.03	   0.43	    1.35	79.24
Macoma cerina	     0.00	     0.14	   0.89	   0.25	    1.16	80.40
Bivalvia	     0.06	     0.06	   0.86	   0.42	    1.12	81.52
Tellina versicolor	     0.07	     0.04	   0.81	   0.34	    1.05	82.57
Anadara floridana	     0.00	     0.12	   0.72	   0.29	    0.94	83.51
Nuculana acuta	     0.12	     0.00	   0.67	   0.33	    0.87	84.38
Timoclea sp.	     0.00	     0.07	   0.61	   0.24	    0.80	85.18
Laevicardium mortoni	     0.02	     0.08	   0.61	   0.40	    0.79	85.97
Carditamera floridana	     0.00	     0.07	   0.49	   0.29	    0.64	86.61
Divaricella quadrisulcata	     0.03	     0.05	   0.47	   0.35	    0.61	87.23
ostreidae	     0.00	     0.07	   0.46	   0.35	    0.61	87.83
Corbula contracta	     0.08	     0.00	   0.46	   0.25	    0.60	88.43
Periploma sp.	     0.08	     0.00	   0.46	   0.28	    0.60	89.03
Anomalocardia auberiana	     0.00	     0.07	   0.45	   0.36	    0.59	89.61
Trachycardium sp.	     0.00	     0.06	   0.44	   0.27	    0.57	90.19

Groups M12  &  M13
Average dissimilarity = 77.57

	Group M12	Group M13	       	       	        	     
Species	 Av.Abund	 Av.Abund	Av.Diss	Diss/SD	Contrib%	Cum.%
Nucula proxima	     2.94	     0.56	  10.36	   1.47	   13.35	13.35
Tellina sp	     1.71	     0.57	   7.53	   1.01	    9.71	23.06
Parvilucina multilineata	     1.93	     0.25	   7.20	   1.01	    9.28	32.33
Macoma tenta	     1.68	     0.62	   6.19	   1.09	    7.98	40.31
Abra aequalis	     1.38	     0.42	   4.61	   1.36	    5.95	46.26
Tagelus divisus	     0.89	     0.33	   3.70	   0.75	    4.76	51.03
Chione sp.	     0.95	     0.59	   2.86	   1.24	    3.69	54.71
Lyonsia floridana	     0.48	     0.21	   2.15	   0.98	    2.77	57.49
Tellina versicolor	     0.53	     0.04	   2.08	   0.46	    2.69	60.17
Crassinella lunulata	     0.47	     0.05	   1.88	   1.03	    2.42	62.60
Lucina sp.	     0.26	     0.24	   1.87	   0.66	    2.41	65.01
Anadara sp.	     0.33	     0.32	   1.87	   0.89	    2.41	67.41
Mulinia lateralis	     0.17	     0.30	   1.72	   0.66	    2.22	69.63
Divaricella quadrisulcata	     0.37	     0.05	   1.69	   0.58	    2.18	71.81
Caryocorbula sp.	     0.33	     0.18	   1.64	   0.91	    2.11	73.93
Amygdalum papyrium	     0.07	     0.23	   1.09	   0.61	    1.40	75.33
Chione cancellata	     0.10	     0.17	   1.07	   0.50	    1.38	76.70
Sphenia sp.	     0.02	     0.25	   1.04	   0.61	    1.35	78.05
Parvilucina sp.	     0.19	     0.03	   0.96	   0.29	    1.23	79.28
Corbula sp.	     0.20	     0.05	   0.91	   0.63	    1.17	80.46
Mysella sp.	     0.07	     0.14	   0.83	   0.56	    1.07	81.52
Crassinella sp.	     0.16	     0.02	   0.80	   0.52	    1.03	82.56
Timoclea sp.	     0.15	     0.07	   0.76	   0.47	    0.98	83.53
Lucinisca nassula	     0.14	     0.00	   0.73	   0.47	    0.94	84.48
Bivalvia	     0.09	     0.06	   0.63	   0.44	    0.81	85.28
Anadara floridana	     0.05	     0.12	   0.61	   0.35	    0.78	86.07
Macoma cerina	     0.00	     0.14	   0.57	   0.25	    0.74	86.81
Nuculana acuta	     0.17	     0.00	   0.54	   0.29	    0.69	87.50
Laevicardium mortoni	     0.02	     0.08	   0.42	   0.41	    0.54	88.05
Tellina mera	     0.09	     0.00	   0.37	   0.21	    0.47	88.52
Mysella planulata	     0.04	     0.06	   0.34	   0.29	    0.44	88.96
Mactridae	     0.06	     0.02	   0.31	   0.33	    0.40	89.37
Carditamera floridana	     0.00	     0.07	   0.31	   0.29	    0.40	89.76
Macoma brevifrons	     0.07	     0.00	   0.30	   0.21	    0.39	90.15

Groups M02  &  M01
Average dissimilarity = 79.89

	Group M02	Group M01	       	       	        	     
Species	 Av.Abund	 Av.Abund	Av.Diss	Diss/SD	Contrib%	Cum.%
Mytilopsis leucophaeata	     4.21	     0.71	  19.97	   0.96	   24.99	24.99
Rangia cuneata	     2.40	     1.59	  16.87	   1.14	   21.12	46.11
Mulinia lateralis	     4.29	     0.22	  16.23	   0.77	   20.31	66.42
Tellina sp	     1.17	     0.02	   6.62	   0.87	    8.29	74.71
Mactridae	     0.28	     0.10	   2.87	   0.40	    3.59	78.30
Macoma tenta	     0.38	     0.00	   2.74	   0.54	    3.43	81.73
Polymesoda sp.	     0.10	     0.00	   2.15	   0.19	    2.69	84.42
Polymesoda caroliniana	     0.32	     0.10	   2.04	   0.42	    2.56	86.98
Rangia sp.	     0.06	     0.07	   1.53	   0.29	    1.92	88.89
Tagelus plebeius	     0.25	     0.00	   1.33	   0.52	    1.66	90.55

Groups M03  &  M01
Average dissimilarity = 90.84

	Group M03	Group M01	       	       	        	     
Species	 Av.Abund	 Av.Abund	Av.Diss	Diss/SD	Contrib%	Cum.%
Mulinia lateralis	     2.92	     0.22	  42.04	   1.67	   46.29	46.29
Rangia cuneata	     0.00	     1.59	  22.96	   1.21	   25.28	71.56
Mytilopsis leucophaeata	     0.95	     0.71	  15.33	   0.84	   16.88	88.44
Tellina sp	     0.10	     0.02	   2.54	   0.39	    2.79	91.23

Groups M04  &  M01
Average dissimilarity = 94.00

	Group M04	Group M01	       	       	        	     
Species	 Av.Abund	 Av.Abund	Av.Diss	Diss/SD	Contrib%	Cum.%
Mulinia lateralis	     4.11	     0.22	  45.11	   1.67	   47.99	47.99
Rangia cuneata	     0.00	     1.59	  23.44	   1.18	   24.93	72.92
Mytilopsis leucophaeata	     0.05	     0.71	   9.34	   0.77	    9.94	82.86
Tagelus divisus	     0.18	     0.00	   3.27	   0.44	    3.48	86.34
Mactridae	     0.00	     0.10	   2.53	   0.25	    2.70	89.04
Tellina sp	     0.11	     0.02	   2.22	   0.41	    2.36	91.40

Groups M05  &  M01
Average dissimilarity = 92.74

	Group M05	Group M01	       	       	        	     
Species	 Av.Abund	 Av.Abund	Av.Diss	Diss/SD	Contrib%	Cum.%
Rangia cuneata	     0.00	     1.59	  31.74	   1.39	   34.23	34.23
Mulinia lateralis	     1.50	     0.22	  30.83	   1.32	   33.24	67.47
Mytilopsis leucophaeata	     0.05	     0.71	  12.71	   0.85	   13.70	81.17
Tagelus divisus	     0.21	     0.00	   5.19	   0.52	    5.59	86.77
Mactridae	     0.00	     0.10	   3.77	   0.28	    4.06	90.83

Groups M06  &  M01
Average dissimilarity = 95.42

	Group M06	Group M01	       	       	        	     
Species	 Av.Abund	 Av.Abund	Av.Diss	Diss/SD	Contrib%	Cum.%
Rangia cuneata	     0.00	     1.59	  19.57	   1.21	   20.51	20.51
Mulinia lateralis	     1.51	     0.22	  17.38	   1.20	   18.21	38.72
Tagelus divisus	     0.83	     0.00	   9.22	   0.97	    9.66	48.38
Mytilopsis leucophaeata	     0.06	     0.71	   8.18	   0.78	    8.57	56.96
Abra aequalis	     0.34	     0.00	   5.73	   0.69	    6.01	62.96
Tellina sp	     0.34	     0.02	   4.93	   0.75	    5.16	68.12
Macoma tenta	     0.29	     0.00	   3.76	   0.57	    3.94	72.07
Chione sp.	     0.18	     0.00	   3.02	   0.58	    3.16	75.23
Parvilucina multilineata	     0.16	     0.10	   2.86	   0.64	    3.00	78.23
Amygdalum papyrium	     0.19	     0.00	   2.08	   0.43	    2.18	80.41
Mactridae	     0.00	     0.10	   1.92	   0.26	    2.01	82.42
Corbula sp.	     0.17	     0.00	   1.73	   0.40	    1.81	84.23
Caryocorbula sp.	     0.14	     0.00	   1.66	   0.45	    1.74	85.98
Lucina sp.	     0.13	     0.00	   1.62	   0.39	    1.70	87.68
Lucinoma filosa	     0.10	     0.00	   1.30	   0.25	    1.36	89.04
Rangia sp.	     0.00	     0.07	   1.20	   0.27	    1.26	90.30

Groups M07  &  M01
Average dissimilarity = 97.70

	Group M07	Group M01	       	       	        	     
Species	 Av.Abund	 Av.Abund	Av.Diss	Diss/SD	Contrib%	Cum.%
Tellina sp	     2.55	     0.02	  12.14	   1.46	   12.43	12.43
Parvilucina multilineata	     1.77	     0.10	   9.02	   1.16	    9.23	21.66
Rangia cuneata	     0.00	     1.59	   7.96	   1.07	    8.14	29.80
Macoma tenta	     1.55	     0.00	   7.27	   0.82	    7.44	37.24
Abra aequalis	     1.30	     0.00	   6.36	   2.48	    6.51	43.75
Tagelus divisus	     0.82	     0.00	   4.40	   0.77	    4.50	48.25
Nucula proxima	     0.87	     0.00	   4.37	   1.22	    4.47	52.72
Chione sp.	     0.85	     0.00	   4.22	   0.99	    4.32	57.03
Mulinia lateralis	     0.68	     0.22	   3.68	   0.92	    3.77	60.80
Mytilopsis leucophaeata	     0.07	     0.71	   3.52	   0.78	    3.61	64.41
Lyonsia floridana	     0.65	     0.00	   3.08	   1.15	    3.15	67.56
Lucina sp.	     0.45	     0.00	   2.43	   0.63	    2.48	70.04
Divaricella quadrisulcata	     0.46	     0.00	   2.38	   0.51	    2.43	72.47
Anadara sp.	     0.40	     0.00	   2.04	   0.76	    2.09	74.56
Amygdalum papyrium	     0.33	     0.00	   1.45	   0.54	    1.48	76.05
Tellina versicolor	     0.21	     0.00	   1.36	   0.30	    1.39	77.43
Caryocorbula sp.	     0.32	     0.00	   1.32	   0.67	    1.35	78.78
Crassinella lunulata	     0.28	     0.00	   1.27	   0.58	    1.30	80.09
Asthenothaerus hemphilli	     0.21	     0.00	   1.25	   0.48	    1.27	81.36
Bivalvia	     0.28	     0.00	   1.20	   0.42	    1.22	82.58
Mactridae	     0.16	     0.10	   1.16	   0.47	    1.19	83.77
Corbula sp.	     0.23	     0.00	   1.12	   0.62	    1.14	84.92
Sphenia sp.	     0.20	     0.00	   1.11	   0.48	    1.13	86.05
Timoclea sp.	     0.25	     0.00	   1.05	   0.46	    1.07	87.12
Lucinidae	     0.16	     0.00	   0.91	   0.27	    0.93	88.05
Crassinella sp.	     0.21	     0.00	   0.87	   0.45	    0.89	88.94
Parvilucina sp.	     0.16	     0.00	   0.68	   0.19	    0.70	89.64
Divaricella dentata	     0.09	     0.00	   0.68	   0.19	    0.69	90.33

Groups M08  &  M01
Average dissimilarity = 97.61

	Group M08	Group M01	       	       	        	     
Species	 Av.Abund	 Av.Abund	Av.Diss	Diss/SD	Contrib%	Cum.%
Abra aequalis	     2.05	     0.00	  11.23	   2.02	   11.50	11.50
Tagelus divisus	     1.96	     0.00	  10.18	   1.40	   10.43	21.93
Rangia cuneata	     0.00	     1.59	   8.36	   1.07	    8.57	30.50
Mulinia lateralis	     1.09	     0.22	   5.72	   1.37	    5.86	36.36
Parvilucina multilineata	     1.08	     0.10	   5.46	   1.48	    5.60	41.95
Nucula proxima	     1.08	     0.00	   5.42	   1.32	    5.55	47.51
Tellina sp	     1.03	     0.02	   5.42	   1.00	    5.55	53.05
Chione sp.	     0.96	     0.00	   5.17	   1.38	    5.29	58.35
Macoma tenta	     0.97	     0.00	   4.88	   1.00	    5.00	63.35
Caryocorbula sp.	     0.79	     0.00	   3.92	   0.83	    4.01	67.36
Mytilopsis leucophaeata	     0.00	     0.71	   3.60	   0.73	    3.69	71.05
Corbula sp.	     0.69	     0.00	   3.52	   1.06	    3.60	74.65
Lyonsia floridana	     0.48	     0.00	   2.60	   0.85	    2.66	77.32
Lucinoma filosa	     0.39	     0.00	   2.40	   0.59	    2.46	79.77
Lucina sp.	     0.34	     0.00	   2.38	   0.53	    2.44	82.21
Asthenothaerus hemphilli	     0.25	     0.00	   1.33	   0.48	    1.37	83.58
Bivalvia	     0.18	     0.00	   1.09	   0.47	    1.12	84.70
Amygdalum papyrium	     0.21	     0.00	   1.04	   0.51	    1.07	85.76
Mactridae	     0.09	     0.10	   1.02	   0.42	    1.05	86.81
Nuculana acuta	     0.20	     0.00	   0.97	   0.46	    0.99	87.80
Corbula contracta	     0.22	     0.00	   0.89	   0.34	    0.91	88.71
Tellinidae	     0.16	     0.00	   0.83	   0.42	    0.85	89.56
Anadara floridana	     0.12	     0.00	   0.80	   0.41	    0.82	90.39

Groups M09  &  M01
Average dissimilarity = 98.46

	Group M09	Group M01	       	       	        	     
Species	 Av.Abund	 Av.Abund	Av.Diss	Diss/SD	Contrib%	Cum.%
Chione sp.	     1.71	     0.00	  11.74	   1.73	   11.92	11.92
Rangia cuneata	     0.00	     1.59	   9.89	   1.16	   10.04	21.96
Tellina sp	     1.06	     0.02	   7.14	   1.14	    7.25	29.21
Tagelus divisus	     1.05	     0.00	   7.03	   0.98	    7.14	36.35
Nucula proxima	     0.97	     0.00	   6.79	   1.56	    6.89	43.25
Abra aequalis	     0.87	     0.00	   5.85	   1.40	    5.94	49.18
Macoma tenta	     0.70	     0.00	   4.62	   0.87	    4.69	53.87
Mytilopsis leucophaeata	     0.00	     0.71	   4.24	   0.77	    4.31	58.18
Parvilucina multilineata	     0.62	     0.10	   4.17	   0.92	    4.24	62.42
Lucina sp.	     0.59	     0.00	   4.12	   0.73	    4.18	66.60
Corbula sp.	     0.51	     0.00	   3.58	   1.01	    3.63	70.23
Lyonsia floridana	     0.44	     0.00	   3.01	   0.95	    3.05	73.29
Mulinia lateralis	     0.32	     0.22	   2.95	   0.75	    2.99	76.28
Chione cancellata	     0.38	     0.00	   2.83	   0.41	    2.87	79.15
Caryocorbula sp.	     0.40	     0.00	   2.75	   0.74	    2.79	81.94
Anadara sp.	     0.31	     0.00	   2.31	   0.62	    2.35	84.29
Mactridae	     0.02	     0.10	   0.91	   0.33	    0.92	85.21
Sphenia sp.	     0.13	     0.00	   0.85	   0.49	    0.86	86.07
Asthenothaerus hemphilli	     0.12	     0.00	   0.82	   0.36	    0.83	86.91
Anadara floridana	     0.12	     0.00	   0.74	   0.33	    0.75	87.66
Divaricella quadrisulcata	     0.11	     0.00	   0.70	   0.45	    0.71	88.37
Lucinisca nassula	     0.10	     0.00	   0.68	   0.44	    0.69	89.06
Bivalvia	     0.11	     0.00	   0.67	   0.44	    0.68	89.74
Timoclea sp.	     0.09	     0.00	   0.62	   0.38	    0.63	90.37

Groups M10  &  M01
Average dissimilarity = 98.24

	Group M10	Group M01	       	       	        	     
Species	 Av.Abund	 Av.Abund	Av.Diss	Diss/SD	Contrib%	Cum.%
Chione sp.	     1.52	     0.00	  12.82	   1.43	   13.05	13.05
Rangia cuneata	     0.00	     1.59	  11.81	   1.15	   12.03	25.08
Mysella sp.	     1.26	     0.00	  10.73	   0.86	   10.92	36.00
Macoma tenta	     1.04	     0.00	   8.82	   0.92	    8.98	44.97
Mulinia lateralis	     0.79	     0.22	   6.93	   0.89	    7.05	52.03
Caryocorbula sp.	     0.86	     0.00	   5.70	   0.89	    5.80	57.82
Tagelus divisus	     0.82	     0.00	   5.58	   0.84	    5.68	63.51
Nucula proxima	     0.71	     0.00	   5.21	   0.94	    5.30	68.81
Mytilopsis leucophaeata	     0.00	     0.71	   5.01	   0.75	    5.10	73.90
Tellina sp	     0.43	     0.02	   3.15	   0.77	    3.21	77.12
Amygdalum papyrium	     0.38	     0.00	   3.02	   0.43	    3.08	80.19
Mysella planulata	     0.44	     0.00	   2.73	   0.43	    2.78	82.97
Abra aequalis	     0.35	     0.00	   2.62	   0.83	    2.67	85.64
Corbula sp.	     0.30	     0.00	   2.40	   0.63	    2.44	88.08
Bivalvia	     0.18	     0.00	   1.62	   0.27	    1.65	89.73
Mactridae	     0.06	     0.10	   1.27	   0.36	    1.29	91.02

Groups M11  &  M01
Average dissimilarity = 98.08

	Group M11	Group M01	       	       	        	     
Species	 Av.Abund	 Av.Abund	Av.Diss	Diss/SD	Contrib%	Cum.%
Rangia cuneata	     0.00	     1.59	  14.06	   1.12	   14.34	14.34
Chione sp.	     1.24	     0.00	  11.06	   1.09	   11.27	25.61
Nucula proxima	     0.95	     0.00	   9.02	   0.81	    9.20	34.81
Tagelus divisus	     0.86	     0.00	   6.63	   0.67	    6.76	41.57
Mulinia lateralis	     0.54	     0.22	   5.95	   0.84	    6.07	47.64
Macoma tenta	     0.69	     0.00	   5.93	   1.07	    6.05	53.69
Mytilopsis leucophaeata	     0.02	     0.71	   5.90	   0.75	    6.01	59.70
Tellina sp	     0.53	     0.02	   5.61	   0.72	    5.72	65.42
Caryocorbula sp.	     0.39	     0.00	   4.57	   0.64	    4.66	70.08
Corbula sp.	     0.47	     0.00	   4.33	   0.83	    4.42	74.50
Abra aequalis	     0.49	     0.00	   4.13	   0.78	    4.21	78.71
Mysella sp.	     0.35	     0.00	   2.85	   0.53	    2.90	81.62
Lyonsia floridana	     0.21	     0.00	   1.63	   0.47	    1.66	83.28
Mactridae	     0.04	     0.10	   1.49	   0.31	    1.52	84.79
Amygdalum papyrium	     0.14	     0.00	   1.43	   0.28	    1.46	86.25
Mysella planulata	     0.14	     0.00	   1.05	   0.38	    1.07	87.32
Chione cancellata	     0.11	     0.00	   1.04	   0.25	    1.06	88.38
Tellina versicolor	     0.07	     0.00	   0.95	   0.26	    0.97	89.35
Bivalvia	     0.06	     0.00	   0.94	   0.30	    0.96	90.31

Groups M12  &  M01
Average dissimilarity = 98.72

	Group M12	Group M01	       	       	        	     
Species	 Av.Abund	 Av.Abund	Av.Diss	Diss/SD	Contrib%	Cum.%
Nucula proxima	     2.94	     0.00	  15.24	   1.71	   15.43	15.43
Tellina sp	     1.71	     0.02	   9.80	   0.91	    9.92	25.36
Parvilucina multilineata	     1.93	     0.10	   8.95	   1.00	    9.07	34.42
Rangia cuneata	     0.00	     1.59	   7.89	   1.07	    7.99	42.42
Macoma tenta	     1.68	     0.00	   7.58	   1.01	    7.68	50.10
Abra aequalis	     1.38	     0.00	   6.48	   1.54	    6.56	56.66
Chione sp.	     0.95	     0.00	   4.94	   1.70	    5.01	61.67
Tagelus divisus	     0.89	     0.00	   3.83	   0.66	    3.88	65.54
Mytilopsis leucophaeata	     0.02	     0.71	   3.43	   0.75	    3.47	69.01
Lyonsia floridana	     0.48	     0.00	   2.56	   0.84	    2.59	71.61
Tellina versicolor	     0.53	     0.00	   2.39	   0.43	    2.42	74.02
Crassinella lunulata	     0.47	     0.00	   2.29	   1.00	    2.32	76.35
Divaricella quadrisulcata	     0.37	     0.00	   1.97	   0.53	    2.00	78.34
Caryocorbula sp.	     0.33	     0.00	   1.93	   0.77	    1.96	80.30
Mulinia lateralis	     0.17	     0.22	   1.86	   0.54	    1.88	82.19
Lucina sp.	     0.26	     0.00	   1.46	   0.44	    1.48	83.67
Anadara sp.	     0.33	     0.00	   1.26	   0.59	    1.27	84.94
Parvilucina sp.	     0.19	     0.00	   1.04	   0.25	    1.06	86.00
Lucinisca nassula	     0.14	     0.00	   0.97	   0.45	    0.98	86.98
Corbula sp.	     0.20	     0.00	   0.94	   0.55	    0.95	87.93
Crassinella sp.	     0.16	     0.00	   0.92	   0.49	    0.93	88.86
Mactridae	     0.06	     0.10	   0.84	   0.36	    0.85	89.71
Nuculana acuta	     0.17	     0.00	   0.64	   0.29	    0.64	90.35

Groups M13  &  M01
Average dissimilarity = 98.33

	Group M13	Group M01	       	       	        	     
Species	 Av.Abund	 Av.Abund	Av.Diss	Diss/SD	Contrib%	Cum.%
Rangia cuneata	     0.00	     1.59	  14.94	   1.21	   15.20	15.20
Chione sp.	     0.59	     0.00	   6.84	   0.94	    6.96	22.16
Mytilopsis leucophaeata	     0.00	     0.71	   6.23	   0.77	    6.34	28.50
Nucula proxima	     0.56	     0.00	   6.17	   0.96	    6.28	34.78
Macoma tenta	     0.62	     0.00	   5.85	   0.77	    5.95	40.73
Tellina sp	     0.57	     0.02	   5.49	   0.77	    5.58	46.31
Mulinia lateralis	     0.30	     0.22	   4.72	   0.59	    4.80	51.11
Abra aequalis	     0.42	     0.00	   4.03	   0.80	    4.10	55.21
Parvilucina multilineata	     0.25	     0.10	   3.19	   0.73	    3.24	58.45
Lucina sp.	     0.24	     0.00	   2.98	   0.47	    3.03	61.48
Anadara sp.	     0.32	     0.00	   2.89	   0.69	    2.94	64.42
Tagelus divisus	     0.33	     0.00	   2.85	   0.50	    2.89	67.31
Sphenia sp.	     0.25	     0.00	   2.33	   0.59	    2.37	69.68
Lyonsia floridana	     0.21	     0.00	   2.22	   0.60	    2.26	71.94
Amygdalum papyrium	     0.23	     0.00	   2.20	   0.53	    2.24	74.18
Chione cancellata	     0.17	     0.00	   2.00	   0.40	    2.03	76.21
Caryocorbula sp.	     0.18	     0.00	   1.98	   0.57	    2.01	78.22
Mactridae	     0.02	     0.10	   1.44	   0.30	    1.46	79.68
Mysella sp.	     0.14	     0.00	   1.41	   0.48	    1.43	81.11
Macoma cerina	     0.14	     0.00	   1.31	   0.26	    1.34	82.45
Timoclea sp.	     0.07	     0.00	   1.10	   0.24	    1.11	83.56
Anadara floridana	     0.12	     0.00	   1.05	   0.29	    1.06	84.63
Rangia sp.	     0.00	     0.07	   0.84	   0.28	    0.86	85.48
Polymesoda caroliniana	     0.00	     0.10	   0.82	   0.28	    0.84	86.32
Laevicardium mortoni	     0.08	     0.00	   0.81	   0.35	    0.83	87.15
Carditamera floridana	     0.07	     0.00	   0.80	   0.29	    0.81	87.96
Trachycardium sp.	     0.06	     0.00	   0.72	   0.27	    0.73	88.69
ostreidae	     0.07	     0.00	   0.71	   0.34	    0.72	89.41
Anomalocardia auberiana	     0.07	     0.00	   0.67	   0.35	    0.68	90.09

Groups M02  &  M14
Average dissimilarity = 73.50

	Group M02	Group M14	       	       	        	     
Species	 Av.Abund	 Av.Abund	Av.Diss	Diss/SD	Contrib%	Cum.%
Mulinia lateralis	     4.29	     4.89	  22.79	   1.07	   31.00	31.00
Mytilopsis leucophaeata	     4.21	     2.46	  16.16	   0.95	   21.99	52.99
Rangia cuneata	     2.40	     0.91	  11.16	   1.08	   15.19	68.18
Tellina sp	     1.17	     0.60	   5.67	   0.89	    7.72	75.90
Corbicula fluminea	     0.00	     0.61	   3.00	   0.32	    4.08	79.98
Macoma tenta	     0.38	     0.36	   2.60	   0.63	    3.54	83.52
Polymesoda caroliniana	     0.32	     0.25	   2.13	   0.58	    2.90	86.42
Mactridae	     0.28	     0.10	   1.67	   0.40	    2.27	88.69
Polymesoda sp.	     0.10	     0.00	   1.05	   0.18	    1.43	90.13

Groups M03  &  M14
Average dissimilarity = 76.11

	Group M03	Group M14	       	       	        	     
Species	 Av.Abund	 Av.Abund	Av.Diss	Diss/SD	Contrib%	Cum.%
Mulinia lateralis	     2.92	     4.89	  31.12	   1.52	   40.89	40.89
Mytilopsis leucophaeata	     0.95	     2.46	  20.83	   1.20	   27.37	68.26
Rangia cuneata	     0.00	     0.91	   7.30	   0.77	    9.60	77.86
Tellina sp	     0.10	     0.60	   5.68	   0.61	    7.46	85.32
Corbicula fluminea	     0.00	     0.61	   4.56	   0.34	    5.99	91.31

Groups M04  &  M14
Average dissimilarity = 81.03

	Group M04	Group M14	       	       	        	     
Species	 Av.Abund	 Av.Abund	Av.Diss	Diss/SD	Contrib%	Cum.%
Mulinia lateralis	     4.11	     4.89	  33.75	   1.47	   41.65	41.65
Mytilopsis leucophaeata	     0.05	     2.46	  20.48	   1.12	   25.27	66.92
Rangia cuneata	     0.00	     0.91	   7.30	   0.75	    9.01	75.93
Tellina sp	     0.11	     0.60	   5.74	   0.57	    7.08	83.01
Corbicula fluminea	     0.00	     0.61	   4.52	   0.33	    5.58	88.59
Macoma tenta	     0.15	     0.36	   2.12	   0.53	    2.62	91.21

Groups M05  &  M14
Average dissimilarity = 86.54

	Group M05	Group M14	       	       	        	     
Species	 Av.Abund	 Av.Abund	Av.Diss	Diss/SD	Contrib%	Cum.%
Mulinia lateralis	     1.50	     4.89	  30.11	   1.26	   34.80	34.80
Mytilopsis leucophaeata	     0.05	     2.46	  24.99	   1.18	   28.87	63.67
Rangia cuneata	     0.00	     0.91	   8.90	   0.78	   10.29	73.95
Tellina sp	     0.00	     0.60	   7.02	   0.53	    8.11	82.06
Corbicula fluminea	     0.00	     0.61	   5.34	   0.34	    6.17	88.23
Tagelus divisus	     0.21	     0.02	   2.38	   0.50	    2.75	90.98

Groups M06  &  M14
Average dissimilarity = 87.48

	Group M06	Group M14	       	       	        	     
Species	 Av.Abund	 Av.Abund	Av.Diss	Diss/SD	Contrib%	Cum.%
Mulinia lateralis	     1.51	     4.89	  23.98	   1.12	   27.41	27.41
Mytilopsis leucophaeata	     0.06	     2.46	  18.67	   1.18	   21.34	48.75
Rangia cuneata	     0.00	     0.91	   6.66	   0.77	    7.61	56.36
Tellina sp	     0.34	     0.60	   5.62	   0.73	    6.42	62.78
Tagelus divisus	     0.83	     0.02	   5.53	   0.83	    6.33	69.11
Corbicula fluminea	     0.00	     0.61	   4.23	   0.34	    4.83	73.94
Macoma tenta	     0.29	     0.36	   3.11	   0.66	    3.55	77.49
Abra aequalis	     0.34	     0.00	   2.93	   0.72	    3.35	80.84
Polymesoda caroliniana	     0.00	     0.25	   1.70	   0.52	    1.94	82.78
Chione sp.	     0.18	     0.00	   1.62	   0.56	    1.85	84.63
Amygdalum papyrium	     0.19	     0.02	   1.34	   0.43	    1.53	86.16
Parvilucina multilineata	     0.16	     0.00	   1.25	   0.54	    1.43	87.59
Bivalvia	     0.05	     0.08	   1.18	   0.37	    1.35	88.94
Lucina sp.	     0.13	     0.02	   1.09	   0.41	    1.24	90.18

Groups M07  &  M14
Average dissimilarity = 92.42

	Group M07	Group M14	       	       	        	     
Species	 Av.Abund	 Av.Abund	Av.Diss	Diss/SD	Contrib%	Cum.%
Mulinia lateralis	     0.68	     4.89	  13.95	   0.87	   15.10	15.10
Mytilopsis leucophaeata	     0.07	     2.46	   9.38	   1.23	   10.15	25.25
Tellina sp	     2.55	     0.60	   8.45	   1.35	    9.14	34.39
Parvilucina multilineata	     1.77	     0.00	   6.99	   1.14	    7.56	41.95
Macoma tenta	     1.55	     0.36	   5.85	   0.83	    6.33	48.28
Abra aequalis	     1.30	     0.00	   4.86	   2.12	    5.26	53.54
Rangia cuneata	     0.00	     0.91	   3.46	   0.72	    3.75	57.29
Nucula proxima	     0.87	     0.00	   3.32	   1.16	    3.60	60.88
Tagelus divisus	     0.82	     0.02	   3.25	   0.77	    3.51	64.39
Chione sp.	     0.85	     0.00	   3.21	   0.98	    3.47	67.86
Lyonsia floridana	     0.65	     0.02	   2.36	   1.12	    2.55	70.42
Corbicula fluminea	     0.00	     0.61	   2.36	   0.34	    2.55	72.97
Lucina sp.	     0.45	     0.02	   1.83	   0.64	    1.98	74.95
Divaricella quadrisulcata	     0.46	     0.00	   1.78	   0.51	    1.93	76.88
Anadara sp.	     0.40	     0.00	   1.55	   0.74	    1.67	78.55
Bivalvia	     0.28	     0.08	   1.22	   0.50	    1.32	79.87
Amygdalum papyrium	     0.33	     0.02	   1.16	   0.55	    1.25	81.12
Caryocorbula sp.	     0.32	     0.00	   1.04	   0.65	    1.13	82.25
Tellina versicolor	     0.21	     0.00	   0.99	   0.29	    1.07	83.32
Crassinella lunulata	     0.28	     0.00	   0.99	   0.57	    1.07	84.39
Asthenothaerus hemphilli	     0.21	     0.00	   0.92	   0.47	    1.00	85.39
Polymesoda caroliniana	     0.00	     0.25	   0.92	   0.51	    0.99	86.38
Corbula sp.	     0.23	     0.00	   0.86	   0.61	    0.93	87.31
Mactridae	     0.16	     0.10	   0.85	   0.45	    0.92	88.23
Timoclea sp.	     0.25	     0.00	   0.84	   0.46	    0.90	89.14
Sphenia sp.	     0.20	     0.00	   0.82	   0.48	    0.89	90.02

Groups M08  &  M14
Average dissimilarity = 92.10

	Group M08	Group M14	       	       	        	     
Species	 Av.Abund	 Av.Abund	Av.Diss	Diss/SD	Contrib%	Cum.%
Mulinia lateralis	     1.09	     4.89	  14.33	   0.91	   15.56	15.56
Mytilopsis leucophaeata	     0.00	     2.46	   9.98	   1.23	   10.84	26.39
Abra aequalis	     2.05	     0.00	   8.39	   1.88	    9.11	35.51
Tagelus divisus	     1.96	     0.02	   7.64	   1.30	    8.29	43.80
Tellina sp	     1.03	     0.60	   4.33	   1.05	    4.70	48.50
Parvilucina multilineata	     1.08	     0.00	   4.31	   1.44	    4.68	53.17
Nucula proxima	     1.08	     0.00	   4.15	   1.25	    4.50	57.68
Macoma tenta	     0.97	     0.36	   4.02	   1.00	    4.36	62.04
Chione sp.	     0.96	     0.00	   3.87	   1.34	    4.20	66.24
Rangia cuneata	     0.00	     0.91	   3.60	   0.72	    3.91	70.15
Caryocorbula sp.	     0.79	     0.00	   3.00	   0.81	    3.26	73.41
Corbula sp.	     0.69	     0.00	   2.69	   1.01	    2.93	76.33
Corbicula fluminea	     0.00	     0.61	   2.44	   0.34	    2.65	78.99
Lyonsia floridana	     0.48	     0.02	   1.93	   0.85	    2.10	81.09
Lucina sp.	     0.34	     0.02	   1.73	   0.54	    1.88	82.96
Lucinoma filosa	     0.39	     0.00	   1.71	   0.61	    1.86	84.82
Bivalvia	     0.18	     0.08	   1.05	   0.53	    1.14	85.96
Asthenothaerus hemphilli	     0.25	     0.00	   1.00	   0.48	    1.09	87.05
Polymesoda caroliniana	     0.00	     0.25	   0.95	   0.51	    1.04	88.09
Amygdalum papyrium	     0.21	     0.02	   0.81	   0.54	    0.88	88.96
Nuculana acuta	     0.20	     0.00	   0.75	   0.45	    0.81	89.77
Corbula contracta	     0.22	     0.00	   0.71	   0.33	    0.77	90.55

Groups M09  &  M14
Average dissimilarity = 94.41

	Group M09	Group M14	       	       	        	     
Species	 Av.Abund	 Av.Abund	Av.Diss	Diss/SD	Contrib%	Cum.%
Mulinia lateralis	     0.32	     4.89	  16.14	   0.88	   17.09	17.09
Mytilopsis leucophaeata	     0.00	     2.46	  11.55	   1.31	   12.23	29.33
Chione sp.	     1.71	     0.00	   8.42	   1.57	    8.92	38.25
Tellina sp	     1.06	     0.60	   5.12	   1.15	    5.42	43.67
Tagelus divisus	     1.05	     0.02	   5.05	   0.93	    5.35	49.02
Nucula proxima	     0.97	     0.00	   4.84	   1.45	    5.13	54.14
Abra aequalis	     0.87	     0.00	   4.21	   1.29	    4.46	58.61
Rangia cuneata	     0.00	     0.91	   4.15	   0.75	    4.40	63.00
Macoma tenta	     0.70	     0.36	   3.76	   0.88	    3.99	66.99
Parvilucina multilineata	     0.62	     0.00	   2.97	   0.84	    3.15	70.14
Lucina sp.	     0.59	     0.02	   2.96	   0.71	    3.13	73.27
Corbicula fluminea	     0.00	     0.61	   2.80	   0.35	    2.97	76.24
Corbula sp.	     0.51	     0.00	   2.55	   0.97	    2.70	78.94
Lyonsia floridana	     0.44	     0.02	   2.15	   0.89	    2.28	81.22
Chione cancellata	     0.38	     0.00	   1.99	   0.40	    2.11	83.33
Caryocorbula sp.	     0.40	     0.00	   1.97	   0.71	    2.08	85.41
Anadara sp.	     0.31	     0.00	   1.62	   0.60	    1.72	87.13
Polymesoda caroliniana	     0.00	     0.25	   1.09	   0.52	    1.16	88.29
Bivalvia	     0.11	     0.08	   0.87	   0.48	    0.92	89.21
Sphenia sp.	     0.13	     0.00	   0.61	   0.48	    0.65	89.86
Asthenothaerus hemphilli	     0.12	     0.00	   0.59	   0.35	    0.62	90.48

Groups M10  &  M14
Average dissimilarity = 92.96

	Group M10	Group M14	       	       	        	     
Species	 Av.Abund	 Av.Abund	Av.Diss	Diss/SD	Contrib%	Cum.%
Mulinia lateralis	     0.79	     4.89	  17.89	   0.95	   19.24	19.24
Mytilopsis leucophaeata	     0.00	     2.46	  13.21	   1.25	   14.21	33.46
Chione sp.	     1.52	     0.00	   8.59	   1.41	    9.24	42.69
Mysella sp.	     1.26	     0.00	   7.18	   0.81	    7.73	50.42
Macoma tenta	     1.04	     0.36	   6.16	   0.98	    6.62	57.05
Rangia cuneata	     0.00	     0.91	   4.72	   0.75	    5.08	62.13
Caryocorbula sp.	     0.86	     0.00	   4.10	   0.85	    4.41	66.54
Tellina sp	     0.43	     0.60	   4.00	   0.86	    4.30	70.84
Tagelus divisus	     0.82	     0.02	   3.97	   0.80	    4.27	75.11
Nucula proxima	     0.71	     0.00	   3.62	   0.88	    3.89	79.00
Corbicula fluminea	     0.00	     0.61	   3.15	   0.34	    3.39	82.38
Amygdalum papyrium	     0.38	     0.02	   2.11	   0.42	    2.27	84.65
Mysella planulata	     0.44	     0.00	   1.98	   0.42	    2.13	86.78
Abra aequalis	     0.35	     0.00	   1.82	   0.79	    1.96	88.74
Corbula sp.	     0.30	     0.00	   1.65	   0.60	    1.78	90.52

Groups M11  &  M14
Average dissimilarity = 93.04

	Group M11	Group M14	       	       	        	     
Species	 Av.Abund	 Av.Abund	Av.Diss	Diss/SD	Contrib%	Cum.%
Mulinia lateralis	     0.54	     4.89	  19.19	   0.93	   20.63	20.63
Mytilopsis leucophaeata	     0.02	     2.46	  14.81	   1.19	   15.92	36.55
Chione sp.	     1.24	     0.00	   7.30	   1.02	    7.84	44.39
Nucula proxima	     0.95	     0.00	   5.89	   0.79	    6.33	50.73
Rangia cuneata	     0.00	     0.91	   5.31	   0.75	    5.70	56.43
Tellina sp	     0.53	     0.60	   5.07	   0.85	    5.45	61.88
Tagelus divisus	     0.86	     0.02	   4.61	   0.64	    4.95	66.83
Macoma tenta	     0.69	     0.36	   4.41	   1.03	    4.74	71.58
Corbicula fluminea	     0.00	     0.61	   3.48	   0.34	    3.74	75.32
Corbula sp.	     0.47	     0.00	   2.82	   0.81	    3.03	78.35
Abra aequalis	     0.49	     0.00	   2.80	   0.74	    3.01	81.35
Caryocorbula sp.	     0.39	     0.00	   2.72	   0.67	    2.92	84.28
Mysella sp.	     0.35	     0.00	   1.95	   0.50	    2.10	86.38
Polymesoda caroliniana	     0.00	     0.25	   1.38	   0.52	    1.48	87.86
Lyonsia floridana	     0.21	     0.02	   1.17	   0.48	    1.26	89.12
Bivalvia	     0.06	     0.08	   1.03	   0.41	    1.11	90.22

Groups M12  &  M14
Average dissimilarity = 95.48

	Group M12	Group M14	       	       	        	     
Species	 Av.Abund	 Av.Abund	Av.Diss	Diss/SD	Contrib%	Cum.%
Mulinia lateralis	     0.17	     4.89	  14.01	   0.84	   14.67	14.67
Nucula proxima	     2.94	     0.00	  11.54	   1.59	   12.08	26.75
Mytilopsis leucophaeata	     0.02	     2.46	   9.46	   1.24	    9.90	36.66
Parvilucina multilineata	     1.93	     0.00	   7.04	   0.96	    7.37	44.03
Tellina sp	     1.71	     0.60	   6.99	   0.98	    7.32	51.35
Macoma tenta	     1.68	     0.36	   6.13	   1.03	    6.43	57.78
Abra aequalis	     1.38	     0.00	   5.00	   1.43	    5.24	63.02
Chione sp.	     0.95	     0.00	   3.74	   1.56	    3.92	66.94
Rangia cuneata	     0.00	     0.91	   3.44	   0.72	    3.61	70.54
Tagelus divisus	     0.89	     0.02	   3.02	   0.63	    3.16	73.70
Corbicula fluminea	     0.00	     0.61	   2.34	   0.34	    2.46	76.16
Lyonsia floridana	     0.48	     0.02	   1.92	   0.84	    2.01	78.17
Tellina versicolor	     0.53	     0.00	   1.84	   0.42	    1.92	80.10
Crassinella lunulata	     0.47	     0.00	   1.76	   0.98	    1.85	81.94
Divaricella quadrisulcata	     0.37	     0.00	   1.49	   0.53	    1.56	83.51
Caryocorbula sp.	     0.33	     0.00	   1.43	   0.75	    1.50	85.00
Lucina sp.	     0.26	     0.02	   1.14	   0.46	    1.19	86.19
Anadara sp.	     0.33	     0.00	   1.01	   0.58	    1.06	87.25
Polymesoda caroliniana	     0.00	     0.25	   0.91	   0.51	    0.96	88.21
Parvilucina sp.	     0.19	     0.00	   0.78	   0.25	    0.82	89.03
Corbula sp.	     0.20	     0.00	   0.73	   0.54	    0.76	89.79
Bivalvia	     0.09	     0.08	   0.70	   0.44	    0.74	90.53

Groups M13  &  M14
Average dissimilarity = 94.69

	Group M13	Group M14	       	       	        	     
Species	 Av.Abund	 Av.Abund	Av.Diss	Diss/SD	Contrib%	Cum.%
Mulinia lateralis	     0.30	     4.89	  20.17	   0.92	   21.30	21.30
Mytilopsis leucophaeata	     0.00	     2.46	  15.80	   1.24	   16.68	37.98
Rangia cuneata	     0.00	     0.91	   5.61	   0.77	    5.93	43.91
Tellina sp	     0.57	     0.60	   5.29	   0.87	    5.58	49.49
Macoma tenta	     0.62	     0.36	   4.38	   0.79	    4.62	54.12
Chione sp.	     0.59	     0.00	   4.15	   0.94	    4.38	58.50
Nucula proxima	     0.56	     0.00	   3.84	   0.93	    4.05	62.55
Corbicula fluminea	     0.00	     0.61	   3.67	   0.34	    3.88	66.43
Abra aequalis	     0.42	     0.00	   2.64	   0.75	    2.79	69.22
Tagelus divisus	     0.33	     0.02	   1.97	   0.49	    2.08	71.30
Anadara sp.	     0.32	     0.00	   1.94	   0.66	    2.05	73.34
Lucina sp.	     0.24	     0.02	   1.85	   0.48	    1.96	75.30
Parvilucina multilineata	     0.25	     0.00	   1.73	   0.63	    1.82	77.12
Sphenia sp.	     0.25	     0.00	   1.50	   0.57	    1.59	78.71
Amygdalum papyrium	     0.23	     0.02	   1.48	   0.53	    1.57	80.28
Polymesoda caroliniana	     0.00	     0.25	   1.45	   0.52	    1.53	81.81
Lyonsia floridana	     0.21	     0.02	   1.43	   0.58	    1.51	83.32
Chione cancellata	     0.17	     0.00	   1.24	   0.39	    1.30	84.63
Caryocorbula sp.	     0.18	     0.00	   1.22	   0.56	    1.29	85.92
Bivalvia	     0.06	     0.08	   0.96	   0.37	    1.02	86.94
Mysella sp.	     0.14	     0.00	   0.90	   0.47	    0.95	87.89
Macoma cerina	     0.14	     0.00	   0.86	   0.24	    0.91	88.80
Anadara floridana	     0.12	     0.00	   0.70	   0.28	    0.74	89.54
Mactridae	     0.02	     0.10	   0.68	   0.27	    0.72	90.26

Groups M01  &  M14
Average dissimilarity = 80.80

	Group M01	Group M14	       	       	        	     
Species	 Av.Abund	 Av.Abund	Av.Diss	Diss/SD	Contrib%	Cum.%
Mulinia lateralis	     0.22	     4.89	  25.81	   0.97	   31.95	31.95
Mytilopsis leucophaeata	     0.71	     2.46	  20.78	   1.10	   25.72	57.67
Rangia cuneata	     1.59	     0.91	  13.36	   1.13	   16.54	74.21
Tellina sp	     0.02	     0.60	   6.29	   0.55	    7.79	81.99
Corbicula fluminea	     0.00	     0.61	   4.96	   0.34	    6.13	88.13
Polymesoda caroliniana	     0.10	     0.25	   2.55	   0.58	    3.16	91.29

Groups M02  &  M15
Average dissimilarity = 93.28

	Group M02	Group M15	       	       	        	     
Species	 Av.Abund	 Av.Abund	Av.Diss	Diss/SD	Contrib%	Cum.%
Mytilopsis leucophaeata	     4.21	     0.49	  22.17	   0.99	   23.77	23.77
Rangia cuneata	     2.40	     0.00	  19.02	   1.04	   20.39	44.15
Mulinia lateralis	     4.29	     0.14	  16.75	   0.77	   17.95	62.11
Bivalvia	     0.02	     0.77	   7.81	   0.89	    8.37	70.48
Tellina sp	     1.17	     0.00	   7.07	   0.85	    7.58	78.06
Macoma tenta	     0.38	     0.00	   2.98	   0.55	    3.19	81.25
Mactridae	     0.28	     0.07	   2.81	   0.42	    3.01	84.26
Polymesoda sp.	     0.10	     0.00	   2.50	   0.20	    2.68	86.93
Polymesoda caroliniana	     0.32	     0.07	   2.25	   0.45	    2.41	89.35
Tagelus plebeius	     0.25	     0.00	   1.41	   0.52	    1.51	90.86

Groups M03  &  M15
Average dissimilarity = 92.44

	Group M03	Group M15	       	       	        	     
Species	 Av.Abund	 Av.Abund	Av.Diss	Diss/SD	Contrib%	Cum.%
Mulinia lateralis	     2.92	     0.14	  48.01	   1.89	   51.93	51.93
Bivalvia	     0.00	     0.77	  17.67	   1.02	   19.12	71.05
Mytilopsis leucophaeata	     0.95	     0.49	  16.81	   0.73	   18.18	89.23
Tellina sp	     0.10	     0.00	   2.64	   0.37	    2.86	92.09

Groups M04  &  M15
Average dissimilarity = 94.21

	Group M04	Group M15	       	       	        	     
Species	 Av.Abund	 Av.Abund	Av.Diss	Diss/SD	Contrib%	Cum.%
Mulinia lateralis	     4.11	     0.14	  50.44	   1.84	   53.54	53.54
Bivalvia	     0.00	     0.77	  18.38	   1.01	   19.51	73.05
Mytilopsis leucophaeata	     0.05	     0.49	   9.19	   0.50	    9.75	82.80
Corbicula sp.	     0.06	     0.07	   3.97	   0.37	    4.21	87.01
Tagelus divisus	     0.18	     0.00	   3.82	   0.45	    4.05	91.06

Groups M05  &  M15
Average dissimilarity = 92.88

	Group M05	Group M15	       	       	        	     
Species	 Av.Abund	 Av.Abund	Av.Diss	Diss/SD	Contrib%	Cum.%
Mulinia lateralis	     1.50	     0.14	  35.04	   1.44	   37.73	37.73
Bivalvia	     0.00	     0.77	  26.71	   1.19	   28.75	66.48
Mytilopsis leucophaeata	     0.05	     0.49	  13.42	   0.58	   14.45	80.93
Tagelus divisus	     0.21	     0.00	   6.13	   0.55	    6.60	87.53
Corbicula sp.	     0.00	     0.07	   3.91	   0.34	    4.21	91.74

Groups M06  &  M15
Average dissimilarity = 94.63

	Group M06	Group M15	       	       	        	     
Species	 Av.Abund	 Av.Abund	Av.Diss	Diss/SD	Contrib%	Cum.%
Mulinia lateralis	     1.51	     0.14	  18.77	   1.24	   19.83	19.83
Bivalvia	     0.05	     0.77	  13.83	   1.05	   14.61	34.45
Tagelus divisus	     0.83	     0.00	  10.32	   1.02	   10.90	45.35
Mytilopsis leucophaeata	     0.06	     0.49	   7.69	   0.51	    8.12	53.47
Abra aequalis	     0.34	     0.00	   6.63	   0.72	    7.01	60.48
Tellina sp	     0.34	     0.00	   5.42	   0.75	    5.72	66.20
Macoma tenta	     0.29	     0.00	   4.24	   0.59	    4.48	70.68
Chione sp.	     0.18	     0.00	   3.45	   0.60	    3.65	74.33
Parvilucina multilineata	     0.16	     0.00	   2.39	   0.58	    2.53	76.86
Amygdalum papyrium	     0.19	     0.00	   2.31	   0.43	    2.44	79.30
Corbula sp.	     0.17	     0.00	   1.92	   0.40	    2.03	81.33
Caryocorbula sp.	     0.14	     0.00	   1.86	   0.46	    1.97	83.30
Lucina sp.	     0.13	     0.00	   1.82	   0.39	    1.92	85.22
Corbicula sp.	     0.00	     0.07	   1.72	   0.31	    1.82	87.04
Lucinoma filosa	     0.10	     0.00	   1.49	   0.25	    1.58	88.62
Polymesoda caroliniana	     0.00	     0.07	   1.37	   0.33	    1.45	90.06

Groups M07  &  M15
Average dissimilarity = 97.60

	Group M07	Group M15	       	       	        	     
Species	 Av.Abund	 Av.Abund	Av.Diss	Diss/SD	Contrib%	Cum.%
Tellina sp	     2.55	     0.00	  12.93	   1.48	   13.24	13.24
Parvilucina multilineata	     1.77	     0.00	   9.82	   1.18	   10.07	23.31
Macoma tenta	     1.55	     0.00	   7.68	   0.83	    7.87	31.18
Abra aequalis	     1.30	     0.00	   6.73	   2.59	    6.90	38.08
Bivalvia	     0.28	     0.77	   4.77	   1.21	    4.88	42.96
Tagelus divisus	     0.82	     0.00	   4.69	   0.77	    4.80	47.76
Nucula proxima	     0.87	     0.00	   4.63	   1.23	    4.74	52.51
Chione sp.	     0.85	     0.00	   4.47	   1.00	    4.58	57.09
Mulinia lateralis	     0.68	     0.14	   3.52	   0.95	    3.60	60.69
Lyonsia floridana	     0.65	     0.00	   3.25	   1.16	    3.33	64.02
Mytilopsis leucophaeata	     0.07	     0.49	   2.99	   0.53	    3.06	67.08
Lucina sp.	     0.45	     0.00	   2.59	   0.63	    2.65	69.73
Divaricella quadrisulcata	     0.46	     0.00	   2.53	   0.51	    2.59	72.33
Anadara sp.	     0.40	     0.00	   2.17	   0.76	    2.22	74.55
Amygdalum papyrium	     0.33	     0.00	   1.53	   0.54	    1.57	76.11
Tellina versicolor	     0.21	     0.00	   1.45	   0.30	    1.49	77.60
Caryocorbula sp.	     0.32	     0.00	   1.39	   0.67	    1.42	79.02
Crassinella lunulata	     0.28	     0.00	   1.34	   0.59	    1.37	80.40
Asthenothaerus hemphilli	     0.21	     0.00	   1.33	   0.49	    1.36	81.76
Sphenia sp.	     0.20	     0.00	   1.18	   0.49	    1.21	82.97
Corbula sp.	     0.23	     0.00	   1.18	   0.62	    1.21	84.18
Timoclea sp.	     0.25	     0.00	   1.10	   0.46	    1.12	85.30
Mactridae	     0.16	     0.07	   1.02	   0.57	    1.04	86.34
Lucinidae	     0.16	     0.00	   0.98	   0.27	    1.00	87.34
Crassinella sp.	     0.21	     0.00	   0.91	   0.46	    0.93	88.27
Divaricella dentata	     0.09	     0.00	   0.73	   0.19	    0.75	89.02
Parvilucina sp.	     0.16	     0.00	   0.71	   0.19	    0.73	89.76
Timoclea grus	     0.11	     0.00	   0.55	   0.33	    0.56	90.32

Groups M08  &  M15
Average dissimilarity = 96.87

	Group M08	Group M15	       	       	        	     
Species	 Av.Abund	 Av.Abund	Av.Diss	Diss/SD	Contrib%	Cum.%
Abra aequalis	     2.05	     0.00	  11.95	   2.06	   12.34	12.34
Tagelus divisus	     1.96	     0.00	  10.81	   1.43	   11.16	23.50
Parvilucina multilineata	     1.08	     0.00	   6.01	   1.55	    6.20	29.70
Tellina sp	     1.03	     0.00	   5.78	   1.00	    5.96	35.66
Mulinia lateralis	     1.09	     0.14	   5.75	   1.39	    5.93	41.59
Nucula proxima	     1.08	     0.00	   5.73	   1.34	    5.92	47.51
Chione sp.	     0.96	     0.00	   5.50	   1.39	    5.67	53.19
Macoma tenta	     0.97	     0.00	   5.18	   1.02	    5.35	58.54
Bivalvia	     0.18	     0.77	   4.51	   1.09	    4.65	63.19
Caryocorbula sp.	     0.79	     0.00	   4.15	   0.83	    4.28	67.47
Corbula sp.	     0.69	     0.00	   3.72	   1.07	    3.84	71.31
Mytilopsis leucophaeata	     0.00	     0.49	   2.89	   0.47	    2.98	74.30
Lyonsia floridana	     0.48	     0.00	   2.77	   0.85	    2.86	77.15
Lucinoma filosa	     0.39	     0.00	   2.58	   0.59	    2.67	79.82
Lucina sp.	     0.34	     0.00	   2.57	   0.53	    2.65	82.47
Asthenothaerus hemphilli	     0.25	     0.00	   1.42	   0.48	    1.46	83.93
Amygdalum papyrium	     0.21	     0.00	   1.11	   0.51	    1.14	85.08
Nuculana acuta	     0.20	     0.00	   1.02	   0.46	    1.05	86.13
Corbula contracta	     0.22	     0.00	   0.94	   0.34	    0.97	87.10
Tellinidae	     0.16	     0.00	   0.88	   0.42	    0.91	88.01
Anadara floridana	     0.12	     0.00	   0.86	   0.42	    0.89	88.90
Mactridae	     0.09	     0.07	   0.82	   0.53	    0.85	89.75
Anadara sp.	     0.17	     0.00	   0.82	   0.43	    0.84	90.59

Groups M09  &  M15
Average dissimilarity = 98.08

	Group M09	Group M15	       	       	        	     
Species	 Av.Abund	 Av.Abund	Av.Diss	Diss/SD	Contrib%	Cum.%
Chione sp.	     1.71	     0.00	  12.61	   1.79	   12.86	12.86
Tellina sp	     1.06	     0.00	   7.73	   1.15	    7.88	20.74
Tagelus divisus	     1.05	     0.00	   7.55	   0.99	    7.69	28.44
Nucula proxima	     0.97	     0.00	   7.30	   1.60	    7.45	35.88
Abra aequalis	     0.87	     0.00	   6.27	   1.44	    6.40	42.28
Bivalvia	     0.11	     0.77	   5.62	   1.24	    5.73	48.01
Macoma tenta	     0.70	     0.00	   4.95	   0.88	    5.05	53.05
Lucina sp.	     0.59	     0.00	   4.43	   0.74	    4.52	57.57
Parvilucina multilineata	     0.62	     0.00	   4.40	   0.89	    4.48	62.05
Corbula sp.	     0.51	     0.00	   3.85	   1.02	    3.93	65.98
Mytilopsis leucophaeata	     0.00	     0.49	   3.45	   0.50	    3.52	69.50
Lyonsia floridana	     0.44	     0.00	   3.23	   0.96	    3.29	72.79
Chione cancellata	     0.38	     0.00	   3.05	   0.42	    3.11	75.91
Caryocorbula sp.	     0.40	     0.00	   2.95	   0.75	    3.01	78.92
Mulinia lateralis	     0.32	     0.14	   2.58	   0.88	    2.63	81.55
Anadara sp.	     0.31	     0.00	   2.50	   0.62	    2.55	84.10
Sphenia sp.	     0.13	     0.00	   0.91	   0.49	    0.93	85.03
Asthenothaerus hemphilli	     0.12	     0.00	   0.88	   0.37	    0.90	85.92
Anadara floridana	     0.12	     0.00	   0.79	   0.33	    0.81	86.73
Divaricella quadrisulcata	     0.11	     0.00	   0.75	   0.45	    0.77	87.50
Lucinisca nassula	     0.10	     0.00	   0.73	   0.45	    0.75	88.25
Mactridae	     0.02	     0.07	   0.68	   0.41	    0.69	88.94
Timoclea sp.	     0.09	     0.00	   0.67	   0.38	    0.68	89.62
Crassinella lunulata	     0.09	     0.00	   0.62	   0.39	    0.64	90.26

Groups M10  &  M15
Average dissimilarity = 97.05

	Group M10	Group M15	       	       	        	     
Species	 Av.Abund	 Av.Abund	Av.Diss	Diss/SD	Contrib%	Cum.%
Chione sp.	     1.52	     0.00	  14.00	   1.46	   14.43	14.43
Mysella sp.	     1.26	     0.00	  11.72	   0.87	   12.08	26.51
Macoma tenta	     1.04	     0.00	   9.63	   0.93	    9.92	36.43
Bivalvia	     0.18	     0.77	   7.88	   1.13	    8.12	44.55
Mulinia lateralis	     0.79	     0.14	   7.06	   0.89	    7.28	51.83
Caryocorbula sp.	     0.86	     0.00	   6.12	   0.90	    6.30	58.13
Tagelus divisus	     0.82	     0.00	   6.02	   0.85	    6.20	64.33
Nucula proxima	     0.71	     0.00	   5.64	   0.95	    5.81	70.14
Mytilopsis leucophaeata	     0.00	     0.49	   4.20	   0.48	    4.33	74.47
Tellina sp	     0.43	     0.00	   3.33	   0.76	    3.43	77.90
Amygdalum papyrium	     0.38	     0.00	   3.29	   0.43	    3.39	81.29
Mysella planulata	     0.44	     0.00	   2.92	   0.44	    3.01	84.30
Abra aequalis	     0.35	     0.00	   2.84	   0.84	    2.93	87.22
Corbula sp.	     0.30	     0.00	   2.60	   0.63	    2.68	89.90
Mactridae	     0.06	     0.07	   0.98	   0.47	    1.01	90.91

Groups M11  &  M15
Average dissimilarity = 96.59

	Group M11	Group M15	       	       	        	     
Species	 Av.Abund	 Av.Abund	Av.Diss	Diss/SD	Contrib%	Cum.%
Chione sp.	     1.24	     0.00	  12.12	   1.12	   12.55	12.55
Nucula proxima	     0.95	     0.00	   9.91	   0.82	   10.26	22.80
Bivalvia	     0.06	     0.77	   8.84	   1.06	    9.15	31.96
Tagelus divisus	     0.86	     0.00	   7.19	   0.67	    7.44	39.40
Macoma tenta	     0.69	     0.00	   6.47	   1.10	    6.70	46.10
Tellina sp	     0.53	     0.00	   6.17	   0.72	    6.39	52.49
Mulinia lateralis	     0.54	     0.14	   5.81	   0.89	    6.01	58.50
Mytilopsis leucophaeata	     0.02	     0.49	   5.21	   0.48	    5.39	63.89
Caryocorbula sp.	     0.39	     0.00	   5.13	   0.65	    5.31	69.21
Corbula sp.	     0.47	     0.00	   4.77	   0.85	    4.94	74.14
Abra aequalis	     0.49	     0.00	   4.50	   0.79	    4.66	78.80
Mysella sp.	     0.35	     0.00	   3.09	   0.54	    3.20	82.00
Lyonsia floridana	     0.21	     0.00	   1.76	   0.48	    1.82	83.82
Amygdalum papyrium	     0.14	     0.00	   1.57	   0.28	    1.63	85.45
Chione cancellata	     0.11	     0.00	   1.14	   0.26	    1.18	86.63
Mysella planulata	     0.14	     0.00	   1.13	   0.38	    1.17	87.80
Mactridae	     0.04	     0.07	   1.07	   0.42	    1.11	88.91
Tellina versicolor	     0.07	     0.00	   1.07	   0.27	    1.11	90.02

Groups M12  &  M15
Average dissimilarity = 98.65

	Group M12	Group M15	       	       	        	     
Species	 Av.Abund	 Av.Abund	Av.Diss	Diss/SD	Contrib%	Cum.%
Nucula proxima	     2.94	     0.00	  16.17	   1.74	   16.39	16.39
Tellina sp	     1.71	     0.00	  10.49	   0.91	   10.63	27.02
Parvilucina multilineata	     1.93	     0.00	   9.60	   1.01	    9.73	36.75
Macoma tenta	     1.68	     0.00	   7.99	   1.02	    8.10	44.85
Abra aequalis	     1.38	     0.00	   6.84	   1.57	    6.93	51.78
Chione sp.	     0.95	     0.00	   5.25	   1.74	    5.32	57.10
Bivalvia	     0.09	     0.77	   4.33	   1.20	    4.39	61.49
Tagelus divisus	     0.89	     0.00	   4.02	   0.66	    4.08	65.57
Mytilopsis leucophaeata	     0.02	     0.49	   2.79	   0.50	    2.83	68.40
Lyonsia floridana	     0.48	     0.00	   2.72	   0.85	    2.76	71.16
Tellina versicolor	     0.53	     0.00	   2.53	   0.44	    2.56	73.72
Crassinella lunulata	     0.47	     0.00	   2.43	   1.01	    2.46	76.18
Divaricella quadrisulcata	     0.37	     0.00	   2.09	   0.53	    2.12	78.30
Caryocorbula sp.	     0.33	     0.00	   2.06	   0.77	    2.09	80.39
Lucina sp.	     0.26	     0.00	   1.56	   0.44	    1.58	81.97
Mulinia lateralis	     0.17	     0.14	   1.55	   0.61	    1.58	83.55
Anadara sp.	     0.33	     0.00	   1.32	   0.60	    1.33	84.88
Parvilucina sp.	     0.19	     0.00	   1.11	   0.25	    1.13	86.00
Lucinisca nassula	     0.14	     0.00	   1.05	   0.45	    1.07	87.07
Corbula sp.	     0.20	     0.00	   0.99	   0.56	    1.00	88.07
Crassinella sp.	     0.16	     0.00	   0.98	   0.49	    0.99	89.07
Mactridae	     0.06	     0.07	   0.67	   0.44	    0.68	89.75
Nuculana acuta	     0.17	     0.00	   0.67	   0.30	    0.68	90.42

Groups M13  &  M15
Average dissimilarity = 97.97

	Group M13	Group M15	       	       	        	     
Species	 Av.Abund	 Av.Abund	Av.Diss	Diss/SD	Contrib%	Cum.%
Bivalvia	     0.06	     0.77	   9.66	   1.16	    9.86	 9.86
Chione sp.	     0.59	     0.00	   7.65	   0.96	    7.81	17.67
Nucula proxima	     0.56	     0.00	   6.86	   0.98	    7.00	24.66
Macoma tenta	     0.62	     0.00	   6.41	   0.78	    6.55	31.21
Tellina sp	     0.57	     0.00	   5.97	   0.77	    6.10	37.31
Mytilopsis leucophaeata	     0.00	     0.49	   5.43	   0.49	    5.54	42.85
Abra aequalis	     0.42	     0.00	   4.42	   0.81	    4.51	47.36
Mulinia lateralis	     0.30	     0.14	   4.41	   0.61	    4.50	51.87
Lucina sp.	     0.24	     0.00	   3.33	   0.48	    3.40	55.27
Anadara sp.	     0.32	     0.00	   3.15	   0.70	    3.22	58.49
Tagelus divisus	     0.33	     0.00	   3.10	   0.51	    3.17	61.65
Parvilucina multilineata	     0.25	     0.00	   3.09	   0.68	    3.16	64.81
Sphenia sp.	     0.25	     0.00	   2.57	   0.60	    2.62	67.43
Lyonsia floridana	     0.21	     0.00	   2.45	   0.61	    2.51	69.94
Amygdalum papyrium	     0.23	     0.00	   2.42	   0.54	    2.47	72.41
Chione cancellata	     0.17	     0.00	   2.22	   0.41	    2.27	74.68
Caryocorbula sp.	     0.18	     0.00	   2.20	   0.58	    2.24	76.92
Mysella sp.	     0.14	     0.00	   1.55	   0.49	    1.58	78.50
Macoma cerina	     0.14	     0.00	   1.44	   0.26	    1.47	79.97
Timoclea sp.	     0.07	     0.00	   1.25	   0.25	    1.27	81.25
Anadara floridana	     0.12	     0.00	   1.14	   0.30	    1.17	82.41
Corbicula sp.	     0.00	     0.07	   1.08	   0.35	    1.10	83.51
Mactridae	     0.02	     0.07	   1.00	   0.40	    1.02	84.54
Polymesoda caroliniana	     0.00	     0.07	   0.95	   0.35	    0.97	85.50
Laevicardium mortoni	     0.08	     0.00	   0.90	   0.36	    0.91	86.42
Carditamera floridana	     0.07	     0.00	   0.89	   0.29	    0.91	87.33
Trachycardium sp.	     0.06	     0.00	   0.80	   0.27	    0.82	88.15
ostreidae	     0.07	     0.00	   0.78	   0.35	    0.80	88.95
Anomalocardia auberiana	     0.07	     0.00	   0.73	   0.36	    0.75	89.70
Corbula sp.	     0.05	     0.00	   0.73	   0.29	    0.74	90.44

Groups M01  &  M15
Average dissimilarity = 94.19

	Group M01	Group M15	       	       	        	     
Species	 Av.Abund	 Av.Abund	Av.Diss	Diss/SD	Contrib%	Cum.%
Rangia cuneata	     1.59	     0.00	  31.34	   1.46	   33.27	33.27
Bivalvia	     0.00	     0.77	  21.83	   1.13	   23.17	56.44
Mytilopsis leucophaeata	     0.71	     0.49	  17.95	   0.89	   19.06	75.50
Mulinia lateralis	     0.22	     0.14	   7.09	   0.59	    7.53	83.03
Mactridae	     0.10	     0.07	   5.02	   0.39	    5.33	88.36
Polymesoda caroliniana	     0.10	     0.07	   3.45	   0.45	    3.67	92.02

Groups M14  &  M15
Average dissimilarity = 89.79

	Group M14	Group M15	       	       	        	     
Species	 Av.Abund	 Av.Abund	Av.Diss	Diss/SD	Contrib%	Cum.%
Mulinia lateralis	     4.89	     0.14	  27.33	   0.98	   30.44	30.44
Mytilopsis leucophaeata	     2.46	     0.49	  24.08	   1.16	   26.82	57.26
Rangia cuneata	     0.91	     0.00	   8.97	   0.79	    9.98	67.24
Bivalvia	     0.08	     0.77	   8.93	   1.00	    9.95	77.19
Tellina sp	     0.60	     0.00	   7.01	   0.55	    7.81	85.00
Corbicula fluminea	     0.61	     0.00	   5.39	   0.34	    6.00	91.00
